# Cyclobutanone Inhibitors of Diaminopimelate Desuccinylase (DapE) as Potential New Antibiotics

**DOI:** 10.3390/ijms25021339

**Published:** 2024-01-22

**Authors:** Thahani S. Habeeb Mohammad, Emma H. Kelley, Cory T. Reidl, Katherine Konczak, Megan Beulke, Janielle Javier, Kenneth W. Olsen, Daniel P. Becker

**Affiliations:** Department of Chemistry and Biochemistry, Loyola University Chicago, 1032 West Sheridan Road, Chicago, IL 60660, USA; thahani.habeebmohammad@abbvie.com (T.S.H.M.); ekelley2@luc.edu (E.H.K.); kkonczak1@luc.edu (K.K.); mbeulke@luc.edu (M.B.); jjavier1@luc.edu (J.J.); kolsen@luc.edu (K.W.O.)

**Keywords:** cyclobutanone, peptidomimetic, antibiotic, enzyme inhibitor, diaminopimelate desuccinylase, DapE, docking, molecular modeling

## Abstract

Based on our previous success in using cyclobutanone derivatives as enzyme inhibitors, we have designed and prepared a 37-member library of α-aminocyclobutanone amides and sulfonamides, screened for inhibition of the bacterial enzyme diaminopimelate desuccinylase (DapE), which is a promising antibiotic target, and identified several inhibitors with micromolar inhibitory potency. Molecular docking suggests binding of the deprotonated hydrate of the strained cyclobutanone, and thermal shift analysis with the most potent inhibitor (**3y**, IC_50_ = 23.1 µM) enabled determination of a K_i_ value of 10.2 +/− 0.26 µM and observed two separate T_m_ values for *H. influenzae* DapE (*Hi*DapE).

## 1. Introduction

The rise of antibiotic-resistant bacteria underscores the need for new antibiotics in the arsenal of drugs to treat bacterial infections [1,2]. In 2018, the World Health Organization (WHO) estimated that 1.5 million of the approximately 10 million people annually who acquire a tuberculosis infection succumb to this devastating chronic infection [3,4]. Particularly pressing is the need for antibiotics with new mechanisms of action. One very attractive target is the dizinc enzyme diaminopimelate desuccinylase (DapE), [5] which is an enzyme in the primary lysine synthetic pathway in all Gram-negative and most Gram-positive bacteria [6]. DapE is thus required for the production of lysine as well as L,L-diaminopimelic acid (L,L-DAP), which is a key component in the production of the bacterial cell wall. Knock-out experiments in *Helicobacter pylori* and *Mycobacterium smegmatis* demonstrate that bacteria cannot survive without DapE, even in lysine supplemented media [7,8]. As mammals, humans do not express DapE, and lysine is an essential dietary amino acid. Earlier, we screened a small library of potential inhibitors of DapE and identified the thiol-containing angiotensin converting enzyme (ACE) inhibitor drug captopril as a low micromolar inhibitor of DapE [9], and thereafter a co-crystal structure of DapE with bound captopril has been reported [10]. Interestingly, Diaz-Sanchez has studied interactions of DapE with flavonoids [11] and with orphenadrine and disulfiram [12]. Very recently we also reported the asymmetric synthesis of the alternate DapE substrate N^6^,N^6^-dimethyl-SDAP and validation of a new ninhydrin-based assay for DapE [13].

Cyclobutanones are an important class of molecules, both as intermediates and as synthetic targets with unique properties [14,15]. The strained four-membered ring endows cyclobutanones with conformational rigidity and also makes the ketone carbonyl more electrophilic relative to an unstrained ketone. Cyclobutanones have demonstrated utility in medicinal chemistry as covalent but reversible serine protease inhibitors, enabled by the electrophilic ketone carbonyl and the accompanying relief of some angle strain when the sp^2^ ketone carbon is rehybridized to sp^3^ upon formation of a hemiacetal with the nucleophilic serine in the active site of a serine protease. Furthermore, cyclobutanones can function as metalloprotease transition-state inhibitors, given the equilibrium of a cyclobutanone with its corresponding hydrate that can interact with active site metals [16]. We reported the facile preparation of a protected 2-aminocyclobutanone synthon, enabling rapid formation of peptidomimetic 2-amido- and 2-sulfonamidocyclobutanones as a potential toward enzyme inhibitor lead molecules that are tunable for recognition by different proteases and esterases with therapeutic potential. Very recently, we reported cyclobutanone inhibitors against SARS-CoV-2 helicase with antiviral activity, identified through in silico screening [17]. Herein, we describe the preparation of a small cyclobutanone library, which we screened against the antibiotic target DapE, identifying a number of 2-aminocyclobutanone derivatives with micromolar inhibitory potency.

## 2. Results

### 2.1. In Silico Druggability Evaluation

We set out to prepare a small library of peptidomimetic lead-like or drug-like 2-amidocyclobutanones and 2-sulfonamidocyclobutanones, selecting a variety of readily available carboxylic acid or acid chloride precursors, and to test the library for DapE inhibitory potency. We prepared a library of 37 amides (Table 1). The compounds were evaluated with SwissADME [18] and revealed no Lipinski, [19] Veber, [20] or Egan [21] violations. The molecular weights vary from 190 to 425, with an average of 284 daltons. The compounds contain two to seven H-bond acceptors (HBA), with an average of 3.9 HBA, and contain one to three H-bond donors (HBD), with an average of 1.83 HBD. The total polar surface area (TPSA) ranges from 46.2 to 127.0 Å^2^, with an average of 75.6 Å^2^. The estimated solubility (ESOL) determination in SwissADME listed 32 library members as soluble and 5 as very soluble. All are predicted to have high gastrointestinal absorption, including 14 with predicted blood–brain barrier (BBB) penetration and 21 without BBB penetration. Among the library members, 32 are predicted to be without cytochrome P450 3A4 (CYP3A4) inhibition, and 5 are predicted to inhibit CYP3A4. There is one Ghose [22] violation (WlogP < −0.4) and three Muegge violations out of the recommended 2–7 pharmacophore points, failing due to too few pharmacophoric units; however, it should be noted that Muegge [23] does not count pyridine as a pharmacophoric unit. There is one Brenk [24] alert due to the presence of a 2-chloropyridine moiety. Two PAINS [25] alerts for library members arose due to the presence of a catechol moiety; however, here the catechol is substituted by a carbonyl, hence it is less electron rich and less reactive. Furthermore, we were encouraged by several approved drugs containing an acyl catechol moiety, namely the D2 antagonist antiemetic trimethobenzamide, troxipide for gastroesophageal reflux disease, and opiranserin, a treatment for postoperative pain in phase II clinical trials.

### 2.2. Synthesis

**Scheme 1:**
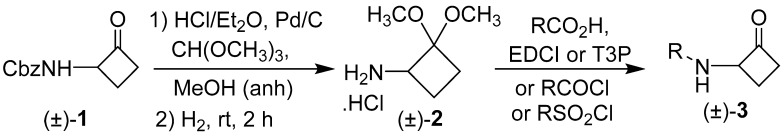
Synthesis of 2-acylaminocyclobutanones **(±)-3a** to **(±)-3af**.

The cyclobutanone derivatives and analogs were prepared from Cbz-protected 2-aminocyclobutanone **(±)-1**, which was converted in two steps in 87–99% yield, in one pot, to the stable, crystalline intermediate **(±)-2**. [26] Amine salt **(±)-2** was coupled with carboxylic acids using either EDCI or T3P as the coupling agent or with an acid chloride. For the preparation of sulfonamides, synthon **(±)-2** was reacted with the requisite sulfonyl chloride. Subsequent treatment with acid to hydrolyze the dimethyl acetal intermediates afforded the 2-acylamino cyclobutanones **(±)-3a** to **(±)-3af**, as shown in Scheme 1.

### 2.3. DapE Inhibition

All compounds were screened for DapE inhibitory potency using a ninhydrin-based assay [27] at 100 μM, and IC_50_ values were determined for molecules that inhibited DapE > 70% at 100 μM (Table 1). Some SAR trends emerge from the data. The most potent analogs were the N-capped amino acid derivatives, with either N-sulfonamides or an N-Cbz group, which are larger and offer greater active site interactions. The most potent analog is the 4-methoxyphenylsulfonamide-D-valine derivative **3y**, with an IC_50_ = 23.1 μM, followed by 3,4,5-trimethoxyphenylamide cyclobutanone **3k**, with an IC_50_ = 39.6 μM. The tosyl-Phe epimer **3v** exhibits an IC_50_ = 45.7 μM which is more potent than the D-Phe epimer **3w** (IC_50_ > 50 μM). The Cbz-protected derivatives of both epimers of phenylalanine (**3ae** and **3ad**) were potent inhibitors, and the IC_50_ values of these two compounds reveals that the D-Phe derivative **3ae** is somewhat more potent (IC_50_ = 55.4 μM) than the L-phenylalanine derivative **3ad** (IC_50_ = 69.8 μM). This preference for the D-epimer is consistent with the p-methoxyphenylsulfonyl-D-Val derivative **3y** as the most potent analog tested (IC_50_ = 23.1 μM) versus the L-Val analog **3z** (30.7% inhibition at 100 μM). Among benzoic acid derivatives, the bulky 3,4,5-trimethoxyphenyl derivative **3k** is the most potent (IC_50_ = 39.6 μM) followed by the meta-chlorobenzamide **3e** (45.8% inhibition at 100 μM), whereas the para-trifluoromethylbenzamide **3f** and the ortho-tolyl benzamide **3a** are less potent (25.2% and 16.8% inhibition, resp., at 100 μM). A few of the simple sulfonamide and amide derivatives exhibited somewhat greater potency, including 4-fluorophenylsulfonamide **3af** (IC_50_ = 53.3 μM) and benzamide derivative **3j** (IC_50_ = 66.4 μM). The extended 2-benzimidazole derivative **3u** exhibited an IC_50_ = 52.4 μM, again offering greater opportunities for ligand-active site interactions. Cyclobutanone hydrate bioisosteres, including cyclopropanol **4a** and *t*-alcohol **4b**, exhibited modest (36.4%) and low (19.1%) inhibition of DapE at 100 μM, respectively, thus the cyclopropyl derivative was approximately twice as potent.

### 2.4. Experimental IC_50_ Curves and Hill Coefficients

All synthesized analogs were assayed for inhibition of *Hi*DapE at 100 µM, and IC_50_ values were determined for more potent analogs (Table 1). Experimental IC_50_ curves for cyclobutanone inhibitors against DapE were identified as sigmoidal shaped response curves, as opposed to simple hyperbolic plots. Further, the calculated Hill coefficients were greater than one, with an average value of 2.8 for all cyclobutanone inhibitors analyzed for IC_50_ determinations. IC_50_ curves with the corresponding Hill coefficients are reported in the Supplementary Materials as Figures S74−S81. Experimental Hill coefficients may provide information on the number of binding sites of a biological target (receptors and enzymes) interacting with ligands [28]. Although n_H_ > 1 could demonstrate the cooperative binding mechanism of ligands with the target enzyme, a Hill coefficient alone does not represent the type of inhibition and is not adequate to distinguish between competitive, noncompetitive, or allosteric binding mechanisms. It is suggested that elucidating mathematical expressions and data fitting for enzyme dose–response curves in the presence of an inhibitor could be challenging compared to the dose–response curves for receptors due to the inclusion of the substrate concentration as an additional unknown parameter [28].

DapE is a dimer with two explicit dizinc binding sites located in the catalytic domains of each subunit. Furthermore, DapE exists in a dynamic equilibrium between open and closed conformers, wherein closure is likely triggered by DapE binding to its native substrate, leading to an active closed form of the enzyme. Therefore, competitive, noncompetitive, or allosteric DapE inhibitors are expected to provide Hill coefficients greater than one, indicating the number of inhibitor-bound binding sites. For example, if two separate inhibitor molecules bind to each of the dizinc active sites of each of the two subunits of DapE, the calculated Hill coefficient for that particular inhibitor could be two or greater than two indicating cooperativity between the two sites. Experimental Hill coefficients for these DapE cyclobutanone inhibitors were determined to have values of 2–3 within the range of error. This is consistent with the statement supported with statistics that the maximum experimentally calculated Hill coefficient should be equal to or smaller than the number of binding sites interacting with the ligands in facilitating the biological response. Based on the mounting high-throughput screening experimental evidence, it is concluded that the shape of the dose–response curve and the corresponding Hill coefficient are determined based on the type of ligand under study [28]. Presently, the binding mode(s) of these cyclobutanone inhibitors and the mechanism leading to the inactivation of DapE are not known with certainty, although we believe that these inhibitors bind to the active site as hydrates based on modeling and docking studies detailed below. 

### 2.5. Thermal Shift Analysis

A thermal shift assay was conducted to observe changes in the thermal stability of DapE under various concentrations of the inhibitor **3y**, as summarized in Table 2. These studies were conducted on a Step One Real-Time PCR System and associated QuantStudio software (QuantStudio 5, Design and Analysis 2) through the fluorescence of the SYPRO Orange dye during protein denaturation. We observed two melt temperatures for *H. influenzae* [13], one at 49.6 °C (T_m_1) and another at 77.5 °C (T_m_2). Upon addition of the inhibitor **3y**, we observed a stabilization of the tertiary structure of the enzyme binding to the inhibitor, as indicated by a positive shift in the first melt temperature (T_m_1). From these data, we were able to calculate the K_i_ of 10.2 µM for compound **3y**, which occurred around the initial positive shift at T_m_1, therefore indicating a thermodynamic change. The thermal shift assay plot of T_m_ vs. log [**3y** (µM)] to determine the K_i_ of compound **3y** is in the Supplementary Materials as Figure S82. At T_m_2, we observed that the inhibitor binding to the enzyme caused a small, but statistically significant, destabilization of the tertiary structure of the enzyme, as evidenced by a reduction in T_m_ from 77.3 °C to 75.7 °C. Binding of a ligand most commonly leads to further stabilization of a protein, but destabilization as indicated by a lower T_m_ has been observed by others [29,30,31]. Based on our previous work, [27] we observed sequential temperatures at which *Hi*DapE was dissociating via circular dichroism: first at about 60 °C, which we hypothesize is the dimer dissociating, and a second at about 80 °C, which may represent denaturation of the monomer.

### 2.6. Cyclobutanone/Hydrate Equilibrium Studies

**Scheme 2:**
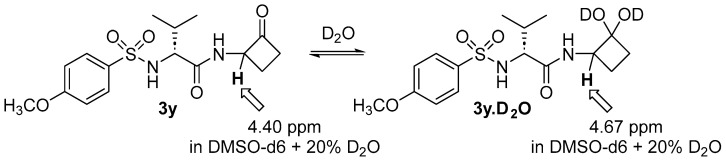
Equilibrium of cyclobutanone **3y** with its hydrate in the presence of D_2_O.

Wiberg studied the hydrates of carbonyl-containing compounds and reported a simple, reproducible method using NMR to measure equilibrium constants of the carbonyl–hydrate equilibrium. He reported that an aqueous solution of unsubstituted cyclobutanone contains only about 2% hydrate when in deuterium oxide [32]. We hypothesized that the electron-withdrawing group attached to the cyclobutanone would shift the equilibrium further towards the hydrated form, and therefore more closely resemble a transition-state mimetic in solution (Scheme 2). 

To conduct these equilibria studies, we followed a protocol similar to Wiberg [32] and chose to study the most potent cyclobutanone, p-methoxybenzenesulfonyl-D-Val-cyclobutanone **3y** (Scheme 2). Cyclobutanone **3y** is soluble in CDCl_3_, but we wanted to vary the amount of D_2_O, and water has a solubility of only 0.815% in chloroform [33], so varying the quantity of D_2_O would not alter the concentration of D_2_O in CDCl_3_ solution once saturation is reached. We therefore selected DMSO-d_6_ as the solvent to enable the varying of the D_2_O concentration, which also provides a more polar environment that is more conducive to hydrate formation and is a better mimic of the environment of the enzyme in aqueous media. The ratio of hydrate to ketone was determined by the ratio of the integrals of the proton alpha to the hydrate/carbonyl and the α-amino group, which was 4.67 ppm for the hydrate in DMSO-d_6_ + 20% D_2_O and 4.40 ppm for the cyclobutanone ketone in DMSO-d_6_ + 20% D_2_O. With 2% D_2_O added to the DMSO-d_6_ solution of **3y**, we observed 35.4% of hydrate present in DMSO-d_6_ (Table 3), and with 20% of D_2_O there was 40% of hydrate present. We found that further increasing the amount of D_2_O in DMSO-d_6_ did not significantly alter the amount of hydrate present. The equilibrium constant K_eq_ = [hydrate]/[ketone] for CDCl_3_ saturated with D_2_O was only K_eq_ = 0.082, whereas for DMSO-d_6_ + 20% D_2_O the equilibrium constant was significantly larger, favoring the hydrate, K_eq_ = 0.58 to 0.68.

### 2.7. Docking Results and Discussion

We previously reported that the DapE enzyme performs the desuccinylase reaction on its substrate through a profound conformational change involving a 10 Å movement of certain amino acid residues of the homodimeric complex [34]. Specifically, this protein flex leads to several loops from the dimerization domain of the opposite peptide chain of the dimeric quaternary structure to close off the crescent-shaped binding cleft of the catalytic domain. This serves to create a transient oxyanion hole made up of one of the catalytic Zn(II) ions (Zn1) and a histidine (His194.B) from the incoming opposite chain, thus enabling scissile-bond cleavage of the substrate. The substantial DapE conformational change was predicted prior to the crystal structure by the computational work of Mishra, who employed principal component analysis of molecular dynamics trajectories of the DapE apo enzyme and the DapE-SDAP complex [35]. The understanding of the enzyme dynamics in regard to how DapE catalyzes native substrate conversion is key to how we have interpreted the results in this docking and modeling study.

The IC_50_ values we report herein are determined from experimental observations and are an expression of the inhibitor’s ability to compete with substate binding to the active site, assuming competitive inhibition. Our docking experiments have focused on the inhibitor binding tightly to the closed form of DapE, and therefore we have utilized the closed conformer X-ray crystal structure (PDB = 5VO3; 1.95 Å resolution) of DapE from *Haemophilus influenzae* for these docking studies.

A second key guideline used in our analysis was to determine if the mode of binding of a generated docking pose conformed to the transition-state mechanism that we have shown is required to trigger conformational closing of the enzyme during native substrate binding [34]. We were able to generate a number of docking poses with high affinity (low energy), but if these docking poses did not conform to the native substate transition state interaction, then the enzyme would not be able to form the closed conformation, and therefore the pose in question would not be able to exist, making these docking poses irrelevant to our analysis. This is particularly important with the cyclobutanone functionality given the equilibrium of the strained ketone with the corresponding hydrate and the existence of epimers at the α-amino group on the cyclobutanone.

The crescent-shaped substrate binding cleft of DapE can be broken down into the succinate and DAP-binding pockets which are divided by the catalytic dimetal binding site. Thus, we define these three binding regions for our discussion. We hypothesize that there could be three main binding paradigms for cyclobutanone inhibitors to interact with the binding pocket of DapE and inhibit *L,L*-SDAP hydrolysis: (1) the cyclobutanone ketone, the ketone hydrate, or a deprotonated-hydrate species (the warhead moiety) may bind to the enzyme at the dizinc center. Binding of the requisite inhibitor form would then involve the body and tail of the ligand occupying the DAP-side of the binding cleft, wherein (2) the warhead group may bind to the dizinc center and allow the rest of the ligand to occupy the succinate-side of the binding cleft, or (3) the rest of the ligand binds to the metal center and the warhead moiety occupies either the succinate binding pocket or the DAP binding pocket. 

**Scheme 3:**
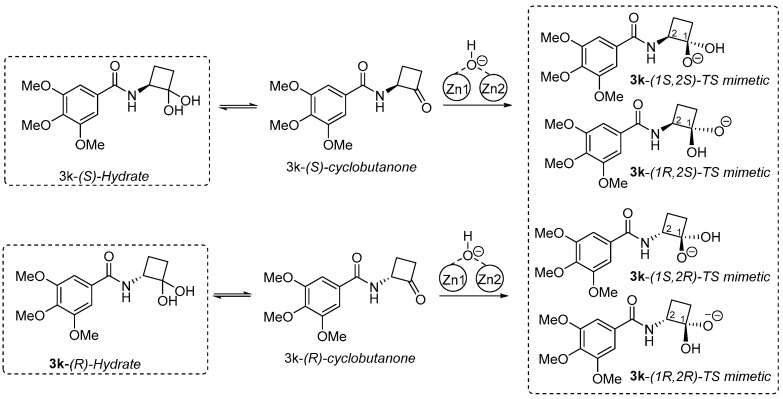
Trimethoxyphenylamide cyclobutanone **3k** hydrate and hydrate-derived ligand species included in the docking studies.

We chose **3k** bearing the 3,4,5-trimethoxybenzamide moiety, which is the most potent analog that lacks an asymmetric center in the side chain (IC_50_ = 39.6 µM). All cyclobutanone inhibitors were synthesized from the racemic α-aminocyclobutanone with respect to the α-carbon next to the cyclobutanone carbonyl group, so we docked both the (*S*)- and (*R*)-isomers of **3k** as the hydrate in our docking library. The tightest binding would be from an anionic species mimicking the oxyanion of the tetrahedral intermediate in the desuccinylation of the actual substrate. An anionic species can be derived from deprotonation of the incoming hydrate, or from attack of the incoming cyclobutanone ketone by the nucleophilic hydroxyl group, as summarized in the ligand models illustrated in Scheme 3. Either pathway would lead to the same bound oxyanion as a transition state [34]. Thus, four species included in our initial docking library, i.e., (*1S*,*2S*), (*1R*,*2S*), (*1S*,*2R*), and (*1R*,*2R*), are illustrated in Scheme 2. Each of these four **3k**-derived ligands were prepared and docked into the closed form of DapE as described previously [34].

**Figure 1:**
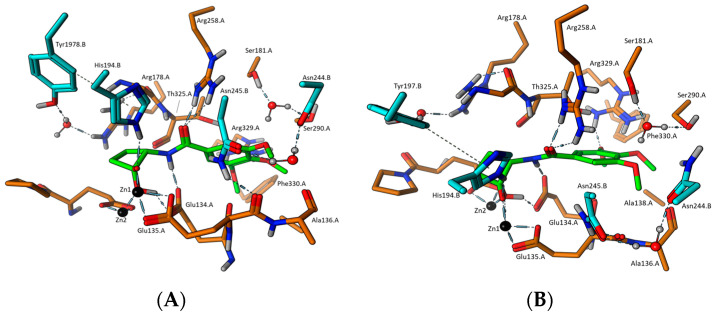
(**A**) Docking model of **3k**-(*1S*,*2R*)-TS mimetic and (**B**) docking pose of **3k**-(*1S*,*2R*)-TS mimetic with 90° roll.

**Figure 2:**
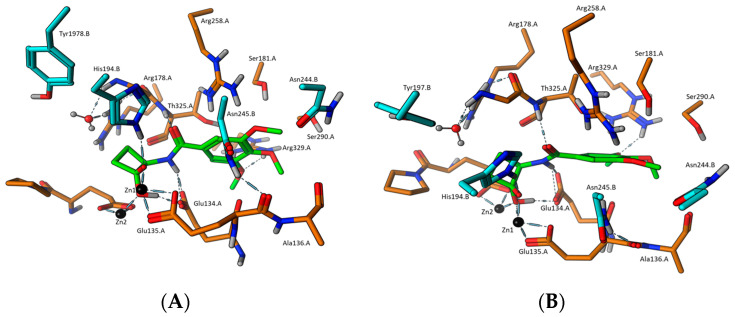
(**A**) Docking model of **3k**-(*1R*,*2R*)-TS mimetic and (**B**) docking pose of **3k**-(*1R*,*2R*)-TS mimetic with 90° roll.

Upon analysis of these initial docking results, it was apparent that neither enantiomer of the neutral hydrates bound as tightly as the deprotonated hydrates, suggesting that the neutral hydrate species is not relevant to binding. Focusing on docking results of the deprotonated species of **3k**, the (*1S*,*2R*) diastereomer docked with a dG = −11.66 kcal/mol (Figure 1), while the (*1R*,*2R*) configuration showed a dG = −10.65 kcal/mol (Figure 2), thus the (*1S*,*2R*) configuration is favored, but the (*1R*,*2R*) was also able to bind comparable to the native substate TS. These docking results suggest that the generation of the transition-state mimetic enables the subsequent formation of strong interactions between the negatively charged oxygen of the ligand with the side chain of His194.B. This, in turn, should trigger the conformational change in the protein to the closed form that provides more interactions to further stabilize the ligand binding in the binding cleft. In addition to the key interactions between Zn1, the negative oxygen of **3k**, and the catalytic histidine from chain B, it may be seen that the neutral oxygen of the ligand’s carbonyl/hydrate group also forms a bridging interaction between both catalytic metals as well as hydrogen bonding with the general acid/base Glu135.A. As observed in the docking results, these important interactions can form with either alpha carbon chirality and still allow interactions between the ligand’s amide group and the protein. In both chiral forms of **3k**, the nitrogen proton forms a hydrogen bond with Glu135.A, while the amide carbonyl simultaneously forms hydrogen bonding interactions with the side chain of Arg258.A. Additionally, the (*1S*,*2R*)-configuration of **3k** causes the cyclobutane ring to push the ligand slightly away from the metal center, so that the carbonyl oxygen atom forms stronger hydrogen bonding with Arg258.A. Further, the cyclobutane ring of the (*1R*,*2R*)-species of **3k** allows the ligand to sit slightly closer to the metal center, causing the carbonyl oxygen to pick up another hydrogen bond with the backbone amide of Thr325.A, at the expense of the interaction strength with Arg258.A. We also see evidence of hydrogen bonding between the side chain of Arg.329.A and the 3-position methoxy group in both **3k** chiral species, with additional hydrogen bonding with the other two methoxy groups seeming possible, but not directly observed in these docking results, with the exception of the HB interaction formed between the 4-poition methoxy group of (*1R*,*2R*)-**3k** and the side residue of Ser290.A.

## 3. Discussion and Conclusions

The alarming rise in antibiotic resistant bacteria calls for the discovery of antibiotics with a new mechanism of action. DapE is a key enzyme in the bacterial lysine biosynthetic pathway and stands out as a promising new antibiotic target. Our interest in cyclobutanones and their ability to serve as peptidomimetics as well as transition-state mimetics to inhibit different families of proteases prompted us to prepare and screen a library of α-aminocyclobutanones to screen for DapE inhibitor potency, providing a number of analogs with micromolar potency. Molecular docking suggests binding to a deprotonated cyclobutanone hydrate, and thermal shift data are consistent with binding near the IC_50_ concentration, while antibiotic testing of the most potent analog prepared has validated antibiotic activity of the most potent member of the series. This series of compounds described herein provides leads for further optimization toward druggable DapE inhibitors as new antibiotics and also provides compounds for co-crystallization with DapE to confirm the hypothesized binding mode.

## 4. Materials and Methods

### 4.1. General Procedures

All reagents were used without further purification, unless otherwise noted, and solvents were distilled before use. Reactions were performed under an atmosphere of nitrogen. For column chromatography, silica gel 60 Å, 40−75 μm (200 × 400 mesh), was utilized, and aluminum-backed silica gel 200 μm plates were used for thin-layer chromatography (TLC). ^1^H (proton) NMR spectra were obtained employing a 500 MHz spectrometer and ^13^C were obtained at 125 MHz, with tetramethylsilane (TMS) as the internal standard. NMR spectra were processed using the Mnova NMR software program (version 12.0.4) provided by Mestrelab Research (Santiago de Compostela, A Coruña, Spain). The ^1^H and ^13^C NMR spectra of all compounds tested are included in the Supplementary Materials as Figures S1−S73 and S83−S87. The purity of all compounds that were assayed was confirmed to be ≥95% as determined with high-performance liquid chromatography (HPLC) utilizing a mobile phase A, composed of 5% acetonitrile in water, and a mobile phase B, composed of 0.1% trifluoroacetic acid (TFA) in acetonitrile, employing a gradient of 60% B increasing to 95% over 10 min, holding at 95% B for 5 min, and then returning to 60% B, and finally holding for 5 min. High-resolution mass spectra (HRMS) spectra were measured on a Time-of-Flight (TOF) instrument utilizing electrospray ionization (ESI). High-resolution mass spectral (HRMS) data were obtained at the Integrated Molecular Structure Education and Research Center (IMSERC, Northwestern University, Evanston, IL, USA) on an Agilent 6210A TOF mass spectrometer in the positive ion mode coupled to an Agilent 1200 series high-performance liquid chromatography (HPLC) system. Data were processed using MassHunter software version B.04.00.

### 4.2. Chemistry

#### 4.2.1. General Procedure for Alpha-Benzamide Cyclobutanones (**3a**-**3j**, and **3s**-**3u**)

To a solution of substituted benzoic acid (1 eq, 0.249 mmol) and acetal **(±)-2** (1.2 eq, 0.298 mmol) in ethyl acetate (1.25 mL), N-methyl morpholine (5.0 eq, 1.24 mmol) was added. Then, propylphosphonic anhydride (2.5 eq, 0.62 mmol) was added to the above mixture as a solution in ethyl acetate (purchased as ≥50 weight % in EA). The reaction mixture was stirred under N_2_ at 80 °C for 48 h or until complete consumption of the benzoic acid determined with TLC (EA/hexane = 50/50 and ~3 to 4 drops of glacial acetic). The reaction was quenched by adding water (3 mL); then, the organic product was extracted using ethyl acetate (3 × 3 mL). The combined organic layers were washed successively with water (3 × 3 mL) and 1N HCl (3 mL) and then dried over Na_2_SO_4_. The solvent was removed via evaporation under reduced pressure providing the corresponding acetal intermediate. Without further purification, crude acetal (1 eq) was subjected to hydrolysis conditions, wherein the crude mixture was dissolved in acetone (1.2 mL), water (0.17 mL, 10% *v*/*v*), and 1N HCl (0.34 mL, 30% *v*/*v*). Then, the reaction mixture was stirred at 50 °C for 18 h with periodic TLC and HPLC monitoring. Upon completion, the organic product was extracted using ethyl acetate (3 × 3 mL) or methylene chloride for highly water-soluble analogs, and the combined organic layers were dried over Na_2_SO_4_. The solvent was evaporated under reduced pressure, and the crude mixture was purified via column chromatography to afford the corresponding alpha-benzamide cyclobutanone.

*2-Methyl-N-(2-oxocyclobutyl)benzamide* (**3a**). The crude product of **3a** was purified via column chromatography using ethyl acetate/hexane (40/60) to afford compound **3a** as a white solid (35.7 mg, 71%): mp 100–102 °C. ^1^H NMR (500 MHz, CDCl_3_) δ 7.40 (dd, J = 7.6, 1.5 Hz, 1H), 7.35 (td, J = 7.5, 1.5 Hz, 1H), 7.30–7.18 (m, 2H), 6.30 (NH, d, J = 7.8 Hz, 1H), 5.21–5.12 (m, 1H), 3.10–2.96 (m, 2H), 2.63–2.52 (m, 1H), 2.47 (s, 3H), 2.16 (dtd, J = 11.0, 9.6, 8.2 Hz, 1H). ^13^C NMR (126 MHz, CDCl_3_) δ 205.0, 169.4, 136.6, 134.8, 131.2, 130.4, 126.8, 125.8, 64.5, 42.3, 19.9. HRMS (ESI) calcd for M+Na^+^ C_12_H_13_NO_2_: 226.0838, found (M+Na^+^) 226.0840.

*3-Methyl-N-(2-oxocyclobutyl)benzamide* (**3b**). The crude product of **3b** was purified via column chromatography on a Teledyne Isco Rf Flash chromatography unit (Teledyne ISCO, Lincoln, NE, USA) eluting with ethyl acetate/hexane (20/80) to afford compound **3b** as a white crystalline solid (25.5 mg, 50%): mp 94–96 °C. ^1^H NMR (500 MHz, CDCl_3_) δ 7.52 (s, 1H), 7.51–7.43 (m, 1H), 7.32–7.21 (m, 2H), 6.56 (NH, d, J = 7.4 Hz, 1H), 5.14–5.05 (m, 1H), 2.95 (dd, J = 9.5, 7.8 Hz, 2H), 2.53–2.43 (m, 1H), 2.32 (d, J = 0.8 Hz, 3H), 2.07 (dtd, J = 11.0, 9.6, 8.2 Hz, 1H). ^13^C NMR (126 MHz, CDCl_3_) δ 204.1, 166.0, 137.5, 132.1, 131.7, 127.6, 127.5, 126.8, 123.0, 76.3, 76.0, 75.7, 63.6, 41.2, 20.3, 18.9. HRMS (ESI) calcd for M+Na^+^ C_12_H_13_NO_2_: 226.0838, found (M+Na^+^) 226.083.

*4-Methyl-N-(2-oxocyclobutyl)benzamide* (**3c**). The crude product of **3c** was purified via column chromatography using ethyl acetate/hexane (40/60) to afford compound **3c** as a white solid (27.4 mg, 50.6%): mp 149–150 °C. ^1^H NMR (500 MHz, CDCl_3_) δ 7.68 (d, 2H), 7.23 (d, *J* = 7.9 Hz, 2H), 6.80 (NH, d, *J* = 7.6 Hz, 1H), 5.19–5.10 (m, 1H), 3.05–2.96 (m, 2H), 2.59–2.48 (m, 1H), 2.40 (s, 3H), 2.18 (dtd, *J* = 11.0, 9.6, 8.1 Hz, 1H). ^13^C NMR (126 MHz, CDCl_3_) δ 205.6, 166.8, 142.5, 130.3, 129.3, 127.1, 64.6, 42.2, 21.5, 19.8. HRMS (ESI) calcd for M+Na^+^ C_12_H_13_NO_2_: 226.0838, found (M+Na^+^) 226.0840.

*2-Chloro-N-(2-oxocyclobutyl)benzamide* (**3d**). The crude product of **3d** was purified via column chromatography on a Teledyne Isco Rf Flash chromatography unit eluting with ethyl acetate/hexane (40/60) to afford cyclobutanone **3d** as a white crystalline solid (32.5 mg, 59%): mp 102–103 °C. ^1^H NMR (500 MHz, CDCl_3_) δ 7.71 (dd, J = 7.6, 1.7 Hz, 1H), 7.45–7.35 (m, 2H), 7.33 (td, J = 7.3, 1.9 Hz, 1H), 6.81 (NH, d, J = 7.2 Hz, 1H), 5.16 (dt, J = 10.3, 7.9 Hz, 1H), 3.03 (dd, J = 9.6, 7.7 Hz, 2H), 2.57 (tt, J = 10.6, 7.2 Hz, 1H), 2.24–2.13 (m, 1H). 1H NMR (500 MHz, CDCl_3_) δ 7.76–7.70 (m, 1H), 7.46–7.35 (m, 2H), 7.35 (ddd, J = 7.5, 6.8, 1.8 Hz, 1H), 6.85 (d, J = 7.6 Hz, 1H), 5.23–5.14 (m, 1H), 3.10–3.01 (m, 2H), 2.64–2.53 (m, 1H), 2.20 (dtd, J = 11.0, 9.6, 8.2 Hz, 1H). ^13^C NMR (126 MHz, CDCl_3_) δ 204.4, 165.8, 133.5, 131.9, 130.8, 130.6, 130.4, 127.2, 64.5, 42.4, 19.7. HRMS (ESI) calcd for MNa+ C_11_H_10_ClNO_2_: 246.0291, found (M+Na^+^) 246.0292.

*3-Chloro-N-(2-oxocyclobutyl)benzamide* (**3e**). The crude product of **3e** was purified via column chromatography using ethyl acetate/hexane (50/50) to afford compound **3e** as a white solid (47.6 mg, 79%): mp 64–66 °C. ^1^H NMR (500 MHz, CDCl_3_) δ 7.77 (t, J = 1.9 Hz, 1H), 7.65 (dt, J = 7.7, 1.4 Hz, 1H), 7.50 (ddd, J = 8.0, 2.1, 1.0 Hz, 1H), 7.38 (t, J = 7.9 Hz, 1H), 6.82 (d, J = 7.1 Hz, 1H), 5.23–5.14 (m, 1H), 3.12–2.97 (m, 2H), 2.62–2.51 (m, 1H), 2.18 (dtd, J = 11.0, 9.6, 8.2 Hz, 1H). ^13^C NMR (126 MHz, CDCl_3_) δ 205.12, 165.61, 134.87, 132.04, 129.99, 127.52, 125.19, 64.51, 42.30, 19.74. HRMS (ESI) calcd for MNa+ C_11_H_10_ClNO_2_: 246.0292, found (M+Na^+^) 246.0280.

*4-Bromo-N-(2-oxocyclobutyl)benzamide* (**3f**). The crude product of **3f** was purified via column chromatography on a Teledyne Isco Rf Flash chromatography unit eluting with ethyl acetate/hexane (30/70) to afford compound **3f** as a white crystalline solid (23.7 mg, 36%): mp 104–105 °C. ^1^H NMR (500 MHz, CDCl_3_) δ 7.69–7.61 (m, 2H), 7.58 (dd, J = 8.6, 2.0 Hz, 2H), 6.85–6.77 (NH, s, 1H), 5.15 (tdd, J = 9.6, 7.8, 1.4 Hz, 1H), 3.04 (ddd, J = 9.5, 7.8, 1.6 Hz, 2H), 2.55 (dtdd, J = 12.3, 9.7, 6.9, 2.1 Hz, 1H), 2.19 (dtd, J = 11.0, 9.6, 8.1 Hz, 1H). ^13^C NMR (126 MHz, CDCl_3_) δ 205.2, 165.9, 131.9, 128.7, 126.8, 64.5, 42.3, 19.7.

*3-Chloro-4-methoxy-N-(2-oxocyclobutyl)benzamide (***3g**). The crude product of **3g** was purified via column chromatography on a Teledyne Isco Rf Flash chromatography unit eluting with ethyl acetate/hexane (40/60) to afford compound **3g** as a white solid (29.5 mg, 47%): mp 134–136 °C. ^1^H NMR (500 MHz, CDCl_3_) δ 7.80 (d, J = 2.2 Hz, 1H), 7.70–7.65 (m, 1H), 6.94 (d, J = 8.8 Hz, 1H), 6.63 (d, J = 7.4 Hz, 1H), 5.18–5.09 (m, 1H), 3.95 (s, 3H), 3.06–2.99 (m, 2H), 2.54 (tt, J = 10.5, 7.2 Hz, 1H), 2.21–2.10 (m, 1H). ^1^H NMR (500 MHz, CDCl_3_) δ 7.80 (d, J = 2.3 Hz, 1H), 7.66 (dd, J = 8.6, 2.3 Hz, 1H), 6.97 (d, J = 7.7 Hz, 1H), 6.92 (d, J = 8.6 Hz, 1H), 5.12 (dt, J = 9.8, 7.6 Hz, 1H), 3.94 (s, 3H), 3.01 (dd, J = 9.4, 7.8 Hz, 2H), 2.57–2.46 (m, 1H), 2.20 (dtd, J = 11.0, 9.5, 8.1 Hz, 1H). ^13^C NMR (126 MHz, CDCl_3_) δ 206.0, 165.4, 157.8, 129.4, 127.3, 126.1, 122.7, 111.5, 64.5, 56.3, 42.2, 19.6.

*2-Chloro-4,5-dimethoxy-N-(2-oxocyclobutyl)benzamide* (**3h**). The crude product of **3h** was purified via column chromatography eluting with ethyl acetate/hexane (60/40) to afford **3h** as a white solid (38.2 mg, 54%): mp 123–125 °C. ^1^H NMR (500 MHz, CDCl_3_) δ 7.43 (s, 1H), 7.40–7.29 (m, 1H), 7.24 (NH, d, J = 7.5 Hz, 1H), 6.83 (s, 1H), 5.13–5.04 (m, 1H), 3.90 (d, J = 4.6 Hz, 6H), 3.14–2.85 (m, 2H), 2.59–2.42 (m, 1H), 2.24 (dddd, J = 11.0, 10.1, 9.0, 8.0 Hz, 1H). 1H NMR (500 MHz, CDCl_3_) δ 7.46 (s, 1H), 7.25 (d, J = 7.6 Hz, 1H), 6.85 (s, 1H), 5.10 (tdd, J = 10.0, 7.1, 2.2 Hz, 1H), 3.92 (d, J = 4.4 Hz, 6H), 3.15–3.05 (m, 1H), 3.08–2.98 (m, 1H), 2.55 (dtd, J = 11.0, 10.1, 5.1 Hz, 1H), 2.26 (dddd, J = 11.1, 10.1, 9.1, 8.1 Hz, 1H). ^13^C NMR (126 MHz, CDCl_3_) δ 204.8, 165.0, 151.5, 148.0, 124.2, 122.8, 113.6, 112.8, 64.8, 56.3, 56.2, 42.3, 19.6.

*2-Hydroxy-N-(2-oxocyclobutyl)benzamide* (**3i**). The crude mixture of **3i** was purified via column chromatography using ethyl acetate/hexane (50/50) to afford cyclobutanone **3i** as a white crystalline solid (49.0 mg, 88%): mp 149–151 °C. ^1^H NMR (500 MHz, CDCl_3_) δ 11.76 (OH, s, 1H), 7.40–7.28 (m, 1H), 7.28 (dd, J = 8.0, 1.6 Hz, 1H), 6.91 (dd, J = 8.4, 1.2 Hz, 1H), 6.88–6.73 (m, 2H), 5.10–5.01 (m, 1H), 3.05–2.92 (m, 2H), 2.55–2.43 (m, 1H), 2.12 (dtd, J = 11.1, 9.6, 8.2 Hz, 1H). ^13^C NMR (126 MHz, CDCl_3_) δ 203.5, 168.6, 160.7, 133.8, 124.6, 117.8, 117.7, 112.3, 62.9, 41.4, 18.7. HRMS (ESI) calcd for M+Na^+^ C_11_H_12_NO_3_: 228.0631, found (M+Na^+^) 228.0600.

*2-Hydroxy-4-methoxy-N-(2-oxocyclobutyl)benzamide* (**3j**). The crude product of **3j** was purified via column chromatography using ethyl acetate/hexane (50/50) to afford cyclobutanone **3j** as a white crystalline solid (38.4 mg, 88%): mp 131–133 °C. ^1^H NMR (500 MHz, CDCl_3_) δ 12.20 (s, 1H), 7.30–7.25 (m, 1H), 6.96 (NH, d, J = 7.7 Hz, 1H), 6.41 (d, J = 2.6 Hz, 1H), 6.37 (dd, J = 8.8, 2.6 Hz, 1H), 5.09 (dt, J = 9.9, 7.7 Hz, 1H), 3.79 (s, 3H), 3.01 (dd, J = 9.4, 7.8 Hz, 2H), 2.55–2.44 (m, 1H), 2.26–2.14 (m, 1H). ^13^C NMR (126 MHz, CDCl_3_) δ 205.0, 169.5, 164.8, 164.0, 126.9, 107.3, 106.3, 101.6, 64.0, 55.5, 42.3, 19.8. HRMS (ESI) calcd for MNa+ C_12_H_13_NO_4_: 258.0737, found (M+Na^+^) 258.0730.

*3,4,5-Trimethoxy-N-(2-oxocyclobutyl)benzamide* (**3k**). Acetal **(±)-2** (30 mg, 0.18 mmol) was suspended in methylene chloride (0.9 mL) and cooled to 0 °C. Triethylamine (49.9 µL, 0.36 mmol) was then added to the solution, followed by dropwise addition of 3,4,5-trimethoxybenzoyl chloride (41.5 mg, 0.18 mmol). The reaction was allowed to stir for 5 h at room temperature with periodic TLC (hexane/diethyl ether = 50/50) and HPLC monitoring. Upon completion, the reaction mixture was successively washed with 1 M HCl (3 mL) and water (3 mL). Then, this methylene chloride layer containing the acetal intermediate was subjected to hydrolysis by vigorously stirring with 1 M HCl (1 mL) overnight. After the hydrolysis was determined to be complete via TLC and HPLC, the organic layer was successively washed with water (3 mL) and brine (3 mL) and dried over Na_2_SO_4_. Then the solvent was removed under reduced pressure, and the resultant crude product was purified via column chromatography using ethyl acetate/hexane (50/50) to afford compound **3k** as a white solid (25 mg, 21%): mp 144–146 °C. ^1^H NMR (500 MHz, CDCl_3_) δ 6.99 (s, 2H), 6.90 (NH, d, J = 7.6 Hz, 1H), 5.11–5.02 (m, 1H), 3.87 (d, J = 1.9 Hz, 9H), 3.10–3.01 (m, 1H), 3.04–2.94 (m, 1H), 2.51 (dtd, J = 11.0, 9.7, 5.6 Hz, 1H), 2.22 (dddd, J = 11.1, 10.0, 9.1, 8.1 Hz, 1H). ^13^C NMR (126 MHz, CDCl_3_) δ 206.1, 166.6, 153.1, 141.3, 128.3, 104.5, 77.3, 77.1, 76.8, 64.7, 60.9, 56.3, 42.2, 19.6. HRMS (ESI) calcd for M+Na^+^ C14H17NO5: 302.0999, found (M+Na^+^) 302.0990.

#### 4.2.2. General Procedure for N-Heterocyclic Benzamide Cyclobutanones (**3l**-**3r**)

The general procedure for the synthesis of benzamide cyclobutanones (**3l**-**3r**) was followed. In order to neutralize and isolate the desired product as the free base, the hydrolysis reaction was quenched by adding saturated Na_2_CO_3_ until effervescence ceased. The organic product was extracted using ethyl acetate (3 × 3 mL) or methylene chloride for highly water-soluble analogs, and the combined organic layers were dried over Na_2_SO_4_. Then, the solvent was evaporated under reduced pressure, and the resultant crude product was purified via column chromatography to afford the heterocyclic benzamide cyclobutanones.

*N-(2-Oxocyclobutyl)picolinamide* (**3l**). The crude product of **3l** was purified via column chromatography using ethyl acetate/hexane (40/60) to afford compound **3l** as a white solid (37.7 mg, 79.5%): mp 97–99 °C. ^1^H NMR (500 MHz, CDCl_3_) δ 8.56 (ddd, *J* = 4.8, 1.7, 0.9 Hz, 1H), 8.50 (NH, d, *J* = 8.1 Hz, 1H), 8.18 (dt, *J* = 7.8, 1.1 Hz, 1H), 7.87 (td, *J* = 7.7, 1.7 Hz, 1H), 7.46 (ddd, *J* = 7.6, 4.8, 1.3 Hz, 1H), 5.22 (dt, *J* = 9.9, 7.8 Hz, 1H), 3.05 (dd, *J* = 9.7, 7.9 Hz, 2H), 2.56 (tt, *J* = 10.6, 7.1 Hz, 1H), 2.24 (dtd, *J* = 11.0, 9.6, 8.3 Hz, 1H). ^13^C NMR (126 MHz, CDCl_3_) δ 205.2, 164.1, 149.0, 148.2, 137.4, 126.6, 122.4, 64.1, 42.3, 19.6. HRMS (ESI) calcd for MNa^+^ C_10_H_10_N_2_O_2_: 213.0634, found (M+Na^+^) 213.0630.

*N-(2-Oxocyclobutyl)nicotinamide* (**3m**). The crude product of **3m** was purified via column chromatography using methanol/ethyl acetate (1/99) to afford compound **3m** as a white solid (25.3 mg, 53%): ^1^H NMR (500 MHz, CDCl_3_)δ 8.98 (dd, J = 2.3, 0.8 Hz, 1H), 8.68 (dd, J = 4.9, 1.7 Hz, 1H), 8.12 (dt, J = 8.0, 2.0 Hz, 1H), 7.68 (d, J = 7.7 Hz, 1H), 7.37 (ddd, J = 8.0, 4.9, 0.9 Hz, 1H), 5.13 (dt, J = 10.3, 7.9 Hz, 1H), 3.01 (dd, J = 9.4, 7.7 Hz, 2H), 2.51 (tt, J = 10.7, 7.1 Hz, 1H), 2.24 (dtd, J = 11.0, 9.6, 8.1 Hz, 1H). ^13^C NMR (126 MHz, CDCl_3_) δ 205.8, 165.2, 152.3, 148.0, 135.6, 129.1, 123.7, 64.4, 42.2, 19.4. HRMS (ESI) calcd for MH+ C_10_H_10_N_2_O_2_: 191.0815, found (M+Na^+^) 191.0810.

*2-Chloro-N-(2-oxocyclobutyl)nicotinamide* (**3n**). The crude product of **3n** was purified via column chromatography using ethyl acetate/hexane (70/30) to afford compound **3n** as a white crystalline solid (25.3 mg, 64%): mp 80–82 °C. ^1^H NMR (500 MHz, CDCl_3_) δ 8.48 (dd, J = 4.7, 2.0 Hz, 1H), 8.14 (dd, J = 7.7, 2.0 Hz, 1H), 7.37 (dd, J = 7.6, 4.7 Hz, 1H), 7.18 (NH, s, 1H), 5.20–5.11 (m, 1H), 3.11–3.03 (m, 2H), 2.64–2.53 (m, 1H), 2.23 (dtd, J = 11.1, 9.6, 8.2 Hz, 1H). ^13^C NMR (126 MHz, CDCl_3_) δ 204.0, 164.1, 151.4, 147.2, 140.2, 129.9, 122.9, 77.3, 77.01, 76.6, 64.5, 42.5, 19.5. HRMS (ESI) calcd for M+Na^+^ C_10_H_9_ClN_2_O_2_: 247.0245, found 247.024 (35Cl), 249.021 (37Cl).

*5-Bromo-N-(2-oxocyclobutyl)nicotinamide* (**3o**). The crude product of **3o** was purified via column chromatography using ethyl acetate/hexane (50/50) to afford compound **3o** as a white crystalline solid (25.3 mg, 64%). %): mp 114–115 °C. ^1^H NMR (500 MHz, CDCl_3_) δ 8.89 (d, *J* = 2.3 Hz, 1H), 8.80 (d, *J* = 2.2 Hz, 1H), 8.27 (t, *J* = 2.1 Hz, 1H), 7.06 (NH, d, *J* = 7.4 Hz, 1H), 5.18 (dddd, *J* = 11.0, 9.5, 8.0, 1.6 Hz, 1H), 3.14–2.99 (m, 2H), 2.58 (tdd, *J* = 10.8, 8.4, 5.8 Hz, 1H), 2.27–2.15 (m, 1H). ^13^C NMR (126 MHz, CDCl_3_) δ 204.7, 163.7, 153.7, 145.9, 138.0, 130.3, 121.0, 77.3, 77.0, 76.8, 64.4, 42.4, 19.6. HRMS (ESI): Calcd for (MH^+^) C_10_H_10_BrN_2_O_2_: 268.9920, found 268.9916 (^79^Br), 269.9948, 270.9896 (^81^Br).

*N-(2-Oxocyclobutyl)isonicotinamide* (**3p**). The crude product of **3p** was purified via column chromatography using methanol/ethyl acetate (1/99) to afford compound **3p** as a white solid (15.4 mg, 33%): mp 119–121 °C. 1H NMR (500 MHz, CDCl_3_) δ 8.77–8.72 (m, 2H), 7.64–7.59 (m, 2H), 7.10 (NH, d, J = 7.4 Hz, 1H), 5.22–5.12 (m, 1H), 3.13–2.98 (m, 2H), 2.64–2.52 (m, 1H), 2.20 (dtd, J = 11.1, 9.6, 8.2 Hz, 1H). ^13^C NMR (126 MHz, CDCl_3_) δ 204.7, 165.0, 150.6, 140.3, 120.9, 77.3, 77.0, 76.8, 64.4, 42.4, 19.6. HRMS (ESI) calcd for MH^+^ C_10_H_10_N_2_O_2_: 191.0815, found 191.0810.

*N-(2-Oxocyclobutyl)quinoline-3-carboxamide* (**3q**). The crude product of **3q** was purified via column chromatography using ethyl acetate/hexane (80/20) to afford compound **3q** as a white solid (30.0 mg, 50%): mp 155–156 °C. ^1^H NMR (500 MHz, CDCl_3_) δ 9.18 (d, J = 2.3 Hz, 1H), 8.52 (dd, J = 2.3, 0.8 Hz, 1H), 8.04 (dd, J = 8.4, 1.1 Hz, 1H), 7.79 (dd, J = 8.2, 1.4 Hz, 1H), 7.73 (ddd, J = 8.4, 6.9, 1.4 Hz, 1H), 7.53 (ddd, J = 8.1, 6.9, 1.2 Hz, 1H), 7.21 (NH, s, 1H), 5.15 (dt, J = 9.9, 7.7 Hz, 1H), 2.99 (dd, J = 9.4, 7.8 Hz, 2H), 2.57–2.46 (m, 1H), 2.19 (dtd, J = 11.1, 9.6, 8.2 Hz, 1H). ^13^C NMR (126 MHz, CDCl_3_) δ 205.3, 165.2, 149.3, 148.0, 136.2, 131.6, 129.3, 128.9, 127.7, 126.8, 125.8, 77.3, 77.0, 76.8, 64.6, 42.4, 19.7. HRMS (ESI) calcd for MNa+ C_14_H_12_N_2_O_2_: 263.0791, found (M+Na^+^) 263.0790.

*N-(2-Oxocyclobutyl)quinoline-8-carboxamide* (**3r**). The crude product of **3r** was purified via column chromatography using ethyl acetate/hexane (70:30) to afford compound **3r** as a white soft solid (11.8 mg, 19%): mp 89–91 °C. ^1^H NMR (500 MHz, CDCl_3_) δ 11.83 (NH, d, J = 7.3 Hz, 1H), 8.93 (dd, J = 4.3, 1.8 Hz, 1H), 8.83 (dd, J = 7.4, 1.6 Hz, 1H), 8.29 (dd, J = 8.4, 1.8 Hz, 1H), 7.99 (dd, J = 8.1, 1.6 Hz, 1H), 7.68 (dd, J = 8.1, 7.3 Hz, 1H), 7.51 (dd, J = 8.3, 4.3 Hz, 1H), 5.25–5.15 (m, 1H), 3.16 (dddd, J = 17.5, 10.1, 4.6, 2.7 Hz, 1H), 3.04 (dddd, J = 17.6, 10.6, 8.8, 2.0 Hz, 1H), 2.56 (qd, J = 10.5, 4.7 Hz, 1H), 2.39 (dddd, J = 11.0, 10.1, 8.8, 8.0 Hz, 1H). ^13^C NMR (126 MHz, CDCl_3_) δ 206.3, 165.7, 149.4, 145.5, 137.8, 134.2, 132.4, 128.5, 127.8, 126.6, 121.0, 65.0, 42.3, 19.7. HRMS (ESI) calcd for MNa+ C_14_H_12_N_2_O_2_: 263.0791, found (M+Na^+^) 263.0810.

*2-(3-Methoxyphenyl)-N-(2-oxocyclobutyl)acetamide* (**3s**). The crude product of **3s** was purified via column chromatography using ethyl acetate/hexane (40/60) where compound **3s** was isolated as an off-white crystalline solid (26 mg, 88% HPLC purity). Recrystallization of the above-mentioned solid from hot ethyl acetate afforded compound **3s** as white crystals (11.4 mg, 20%): mp 104–105 °C. ^1^H NMR (500 MHz, CDCl_3_) δ 7.27 (dd, J = 8.3, 7.4 Hz, 1H), 6.87–6.81 (m, 2H), 6.80 (t, J = 2.1 Hz, 1H), 5.94 (NH, d, J = 7.4 Hz, 1H), 4.86 (dtd, J = 9.6, 7.4, 6.7, 1.2 Hz, 1H), 3.81 (s, 3H), 3.56 (s, 2H), 2.99–2.84 (m, 2H), 2.44–2.33 (m, 1H), 2.02 (dtd, J = 11.0, 9.6, 8.1 Hz, 1H). ^13^C NMR (126 MHz, CDCl_3_) δ 205.2, 170.5, 160.1, 135.7, 130.2, 121.7, 115.0, 113.1, 64.2, 55.3, 43.2, 42.01, 19.5. HRMS (ESI) calcd for MNa^+^ C_13_H_15_NO_3_: 256.0944, found (M+Na^+^) 256.095.

*2-(4-Bromophenyl)-N-(2-oxocyclobutyl)acetamide* (**3t**). The crude product of **3t** was purified via column chromatography using ethyl acetate/hexane (50/50) isocratic elution to afford compound **3t** as a white crystalline solid (49.7 mg, 71%): mp 124–127 °C. ^1^H NMR (500 MHz, CDCl_3_) δ 7.51–7.43 (m, 2H), 7.20–7.11 (m, 2H), 5.95 (d, 1H), 4.89 (dt, *J* = 10.3, 8.0 Hz, 1H), 3.53 (s, 2H), 3.02–2.86 (m, 2H), 2.40 (tt, *J* = 10.5, 7.2 Hz, 1H), 2.01 (dtd, *J* = 11.1, 9.5, 8.0 Hz, 1H). ^13^C NMR (126 MHz, CDCl_3_) δ 205.1, 170.0, 133.2, 132.2, 131.1, 121.6, 64.2, 42.4, 42.1, 19.5. HRMS (ESI) calcd for MNa^+^ C_12_H_12_BrNO_2_: 303.9944, found (M+Na) 303.994 (100%) 304.997 (13.56%).

*3-(1H-Benzo[d]imidazol-2-yl)-N-(2-oxocyclobutyl)propenamide* (**3u**). 2-Benzimidazolepropionic acid was reacted with acetal **(±)-2** following the general T3P coupling reaction method. Upon the completion of the acetal hydrolysis, acetone was evaporated off under reduced pressure. Then, the reaction was quenched by adding saturated Na_2_CO_3_ until effervescence ceased. The organic product was extracted using methylene chloride (5 × 2 mL) and the combined organic layers were dried over Na_2_SO_4_. Then, the solvent was evaporated under reduced pressure, and the resultant crude product was purified via column chromatography using methanol/ethyl acetate (3/97) isocratic elution to afford an off-white solid with 93% HPLC purity. This solid was washed with ethyl acetate (3 × 0.5 mL) to yield cyclobutanone **3u** as a white crystalline solid (10.5 mg, 16%): mp 179–181 °C. ^1^H NMR (500 MHz, CDCl_3_) δ 7.54 (dt, J = 6.7, 3.3 Hz, 2H), 7.26 (NH, s, 1H), 7.21 (dt, J = 7.0, 3.5 Hz, 2H), 7.14 (NH, d, J = 7.6 Hz, 1H), 4.78 (tdd, J = 7.7, 5.9, 4.2 Hz, 1H), 3.27–3.13 (m, 2H), 3.03–2.87 (m, 2H), 2.82–2.62 (m, 2H), 2.44–2.31 (m, 1H), 2.12 (tt, J = 10.0, 9.0 Hz, 1H). ^13^C NMR (126 MHz, CDCl_3_) δ 205.8, 172.6, 153.7, 122.4, 64.4, 42.1, 33.4, 24.6, 19.1. HRMS (ESI) calcd for MH^+^ C_14_H_16_N_3_O_2_: 258.1237, found (MH^+^) 258.1230.

#### 4.2.3. General Procedure for Synthesis of N-Aryl Sulfonamide-Amino Acid Intermediates

Following the general method reported by Mishra [36], to a vigorously stirring solution of amino acid (1.21 mmol) in DI water (1.5 mL), sodium carbonate (179.8 mg, 1.45 mmol) was added. Once all the solutes were dissolved, the solution was cooled to 0 °C, and the respective aryl sulfonyl chloride (1.45 mmol) was added to the stirring reaction mixture in four portions over 1 h. Then, the reaction was allowed to warm up to rt, and the slurry was stirred at rt for 4 h or until completion. The reaction progress was monitored via TLC (methanol/methylene chloride = 10/90). Once completed, the mixture was acidified using 2 N HCl until the pH of the solution was reduced to pH 2. Crystals formed during the acidification were filtered and washed with pH 2.2 buffer. Final pure products (**3v**-**3x**) were dried under high vacuum. Both proton and carbon NMR spectra of pure products **3v**-**3x** matched the reported characterization data as indicated.

#### 4.2.4. General Procedure for the Synthesis of Amino Acid Cyclobutanone Analogs **3v**-**3ae**

To a solution of substituted *N*-benzene sulfonamide amino acids (1 eq) and acetal **(±)-2** (1.2 eq) in ethyl acetate (0.2 M), *N*-methyl morpholine (4.0 eq) was added. Then, propylphosphonic anhydride (T3P, 2.5 eq) was added to the above mixture as a solution in ethyl acetate (purchased as ≥50 weight % in EA). The reaction mixture was stirred under N_2_ at rt to 40 °C for 18 h or until complete consumption of protected carboxylic acid intermediates determined with HPLC analysis. The reaction was quenched by adding water (3 mL) and then the organic product was extracted using ethyl acetate (3 × 3 mL). The combined organic layers were washed successively with water (3 × 3 mL) and 1N HCl (3 mL) and then dried over Na_2_SO_4_. The solvent was removed via evaporation under reduced pressure, providing the corresponding acetal intermediate. Without further purification, crude acetal (1 eq, final concentration was 0.1 M) was subjected to hydrolysis conditions, wherein the crude mixture was dissolved in acetone, water (10% *v*/*v*), and 1N HCl (30% *v*/*v*). Then, the reaction mixture was stirred at 40 °C overnight with periodic HPLC monitoring. Upon completion, the organic product was extracted using ethyl acetate (3 × 3 mL) or methylene chloride for highly water-soluble analogs, and the combined organic layers were dried over Na_2_SO_4_. The solvent was evaporated under reduced pressure, and the crude mixture was purified via column chromatography to afford the corresponding amino acid-derived cyclobutanone (**3v**-**3ae**).

*(2S)-2-((4-Methylphenyl)sulfonamido)-N-(2-oxocyclobutyl)-3-phenylpropanamide* (**3v**). Tosyl-L-phenylalanine (50.0 mg, 0.129 mmol) was prepared according to Mishra [36] and was employed in the reaction (50.0 mg, 0.129 mmol). The crude mixture of **3v** was purified via column chromatography eluting with a gradient of ethyl acetate/hexane (20/80) and ethyl acetate/hexane (40/60) followed by recrystallization from hot EA to afford compound **3v** (9.8 mg, 20%) as white crystalline solid. ^1^H NMR (500 MHz, CDCl_3_) δ 7.57–7.44 (m, 2H), 7.25–7.15 (m, 5H), 6.96–6.87 (m, 2H), 6.72 (d, J = 8.3 Hz, 1H), 4.97 (dtt, J = 10.1, 8.2, 2.1 Hz, 1H), 4.85 (d, J = 6.6 Hz, 1H), 3.85 (dt, J = 7.3, 6.4 Hz, 1H), 3.01–2.89 (m, 3H), 2.88 (dddd, J = 17.4, 9.9, 4.7, 2.3 Hz, 1H), 2.43 (s, 3H), 2.46–2.35 (m, 1H), 1.86 (dtd, J = 11.1, 9.7, 8.3 Hz, 1H). HRMS (ESI): Calcd for (MH^+^) C_20_H_23_N_2_O_4_S: 387.1373, found 387.1366.

*(2R)-2-((4-Methylphenyl)sulfonamido)-N-(2-oxocyclobutyl)-3-phenylpropanamide* (**3w**). Tosyl-D-phenylalanine (50.0 mg, 0.129 mmol) was prepared according to Mishra [36] and was employed in the reaction. The crude mixture of **3w** was purified via column chromatography eluting with a gradient of ethyl acetate/hexane (20/80) and ethyl acetate/hexane (30/70) to afford compound **3w** (18.1 mg, 36.2%) as a white solid: mp 190–192 °C. ^1^H NMR (500 MHz, CDCl_3_) δ 7.57–7.48 (m, 2H), 7.24–7.14 (m, 5H), 6.95–6.86 (m, 2H), 6.75 (NH, d, J = 8.3 Hz, 1H), 4.98 (dtt, J = 10.2, 8.3, 1.9 Hz, 0.5H), 4.87 (NH, d, J = 6.6 Hz, 0.5H), 4.82 (NH, d, J = 6.8 Hz, 0.5H), 4.78–4.68 (m, 0.5H), 3.87 (dtd, J = 16.8, 7.0, 6.1 Hz, 1H), 3.07–2.93 (m, 1.5H), 2.96–2.91 (m, 1H), 2.93–2.85 (m, 1H), 2.83 (dd, J = 14.1, 5.8 Hz, 0.5H), 2.44 (s, 1.5H), 2.43 (s, 1.5H), 2.43–2.36 (m, 0.5H), 2.39–2.31 (m, 0.5H), 2.16 (dddd, J = 11.2, 10.3, 8.9, 8.0 Hz, 0.5H), 1.86 (dtd, J = 11.2, 9.7, 8.4 Hz, 0.5H). ^13^C NMR (126 MHz, CDCl_3_) δ 204.5, 204.4, 170.2, 169.9, 144.2, 144.1, 135.4, 135.3, 134.9, 134.7, 130.0, 129.9, 129.3, 129.1, 129.0, 127.4, 127.4, 127.2, 127.2, 77.3, 77.0, 76.8, 64.3, 63.7, 57.6, 57.3, 42.3, 42.1, 38.2, 37.7, 21.6, 21.6, 19.4, 18.9.

*(2S)-3-(1H-Indol-3-yl)-2-((4-methylphenyl)sulfonamido)-N-(2-oxocyclobutyl)propanamide* (**3x**). Tosyl-L-tryptophan was prepared according to Mishra [36] and was employed in the reaction (50.0 mg, 0.139 mmol). The crude mixture of **3x** was purified via column chromatography on a Teledyne Isco Rf Flash chromatography unit eluting with a gradient of ethyl acetate/hexane (20/80), ethyl acetate/hexane (30/70), and ethyl acetate/hexane (40/60) to afford compound **3x** (12.4 mg, 21%) as an off-white solid: mp 200–201 °C. ^1^H NMR (500 MHz, acetonitrile-*d*_3_) δ 9.07 (NH, s, 1H), 7.48–7.41 (m, 2H), 7.41–7.32 (m, 2H), 7.16–7.09 (m, 3H), 7.10 (NH, d, *J* = 7.5 Hz, 1H), 7.07–6.96 (m, 2H), 5.82 (NH, s, 1H), 4.68 (dtt, *J* = 10.2, 8.0, 2.2 Hz, 1H), 3.90 (dd, *J* = 8.1, 5.7 Hz, 1H), 3.14–3.06 (m, 1H), 2.95–2.73 (m, 3H), 2.35 (s, 3H), 2.21–2.10 (m, 1H), 1.82 (tt, *J* = 10.5, 8.6 Hz, 1H). ^13^C NMR (126 MHz, CD_3_CN) δ 205.6, 170.7, 143.5, 136.6, 136.4, 129.4, 127.1, 126.6, 126.5, 124.1, 121.4, 118.9, 111.3, 109.0, 63.6, 63.6, 57.0, 41.4, 41.3, 28.7, 28.6, 20.6, 20.6, 18.2, 18.1. HRMS (ESI): Calcd for (MH^+^) C_22_H_24_N_3_O_4_S: 426.1482, found 426.1472.

*General procedure of D-valine sulfonamide para-phenyl substituents* (**3y**, **3aa**-**3ac**) Valine *tert*-butyl ester HCl salt (0.477 mmol) was dissolved in anhydrous pyridine (1.0 mL) and stirred until all the solutes were dissolved. The reaction mixture was cooled to 0 °C with an ice bath, and the respective para-benzene sulfonyl chloride (0.477 mmol) was added to the stirring reaction mixture. Then, the reaction was allowed to warm up to rt and stirred at rt for 24 h with periodic monitoring using HLPC. Upon completion, the reaction mixture was diluted with methylene chloride (3 mL), and the organic layer was washed successively with water (3 mL), 1N HCl (10 × 3 mL), and brine (3 mL) and then dried over Na_2_SO_4_. The solvent was removed via evaporation under reduced pressure providing a pale-yellow solid. Without further purification, the respective phenyl-D-valine tert butyl esters were subjected to hydrolysis conditions. To a solution of crude phenyl-D-valine *tert*-butyl ester (0.188 mmol) in methylene chloride (0.94 mL), TFA (282 µL, 30% *v*/*v*) was added under nitrogen. The mixture was stirred at room temperature overnight and monitored via TLC (EA/hexane = 50/50). The solvent was evaporated under reduced pressure. To the resultant residue, toluene (2 mL) was added to enable the formation of an azeotrope. The excess TFA was removed via evaporation on a rotary evaporator at 50 °C providing a yellow crystalline solid with yields between 90–99%. The intermediates were immediately subjected to coupling conditions to form cyclobutanones **3y**, **3aa**-**3ac**.

*(2R)-2-((4-Methoxyphenyl)sulfonamido)-3-methyl-N-(2-oxocyclobutyl)butanamide* (**3y**). D-Cyclobutanone **3y** was synthesized from the intermediate [37] described in the general procedure of D-valine sulfonamide para-phenyl substituents. Compound **3y** was synthesized from this intermediate (30 mg and 0.104 mmol) following the general procedure of the T3P coupling reactions of N-Ts amino acids with 2-aminocyclobutanone synthon **(±)-2**, and the acetal intermediate was subjected to hydrolysis using general reaction conditions (1 M HCl, acetone, and H_2_O) and stirred at 40 °C for 18 h. The crude mixture of **3y** was purified via column chromatography using ethyl acetate/hexane (60/40) to afford Ts-D-valine cyclobutanone analog **3y** (15.5 mg, 42%) as a white solid: mp 179–181. ^1^H NMR (500 MHz, CDCl_3_) δ 7.84–7.77 (m, 2H), 7.04–6.96 (m, 2H), 6.51 (dd, J = 16.8, 7.7 Hz, 1H), 5.08 (dd, J = 13.7, 7.8 Hz, 1H), 4.89–4.76 (m, 1H), 3.90 (s,1.5H), 3.89 (s, 1.5H), 3.48 (ddd, J = 16.5, 7.8, 5.1 Hz, 1H), 2.99–2.87 (m, 2H), 2.43–2.30 (m, 1H), 2.17–2.06 (m, 1H), 2.01–1.94 (m, 0.5H), 1.87 (dtd, J = 11.0, 9.6, 8.1 Hz, 0.5H), 0.87 (dd, J = 6.9, 3.2 Hz, 3H), 0.81 (t, J = 6.7 Hz, 3H). ^13^C NMR (126 MHz, CDCl_3_) δ 204.2, 170.4, 170.3, 163.3, 130.5, 129.6, 114.4, 64.1, 63.9, 61.8, 61.8, 55.7, 42.3, 42.2, 31.1, 31.1, 19.2, 19.0, 17.2, 17.1. HRMS (ESI): Calcd for (MH+) C_16_H_23_N_2_O_5_S: 355.1322, found 355.1309.

*(2S)-2-((4-Methoxyphenyl)sulfonamido)-3-methyl-N-(2-oxocyclobutyl)butanamide* (**3z**). The starting material ((4-methoxyphenyl)sulfonyl)-*L*-valine was synthesized as described by Marques et al. [37], and the ^1^H and ^13^C NMR matched spectra reported in the literature [38]. Without further purification, ((4-methoxyphenyl)sulfonyl)-*L*-valine was coupled with the 2-aminocyclobutanone synthon following the general procedure of the T3P coupling reactions of *N*-Ts amino acids. The acetal intermediate was subjected to hydrolysis using general reaction conditions (1 M HCl, acetone, and H_2_O) and stirred at 40 °C for 18 h to afford (2*S*)-2-((4-methoxyphenyl)sulfonamido)-3-methyl-*N*-(2-oxocyclobutyl)butanamide. Further purification on the crude mixture was conducted via rinsing the solid with ether three times to afford analog **3z** (15.3 mg, 15%) as a white solid: mp 179–181 °C. ^1^H NMR (500 MHz, CHCl_3_) δ 7.75–7.67 (m, 2H), 6.94–6.85 (m, 2H), 6.42 (dd, J = 15.7, 7.6 Hz, 1H), 4.99 (dd, J = 11.8, 7.8 Hz, 1H), 4.79–4.66 (m, 1H), 3.81 (d, J = 5.0 Hz, 3H), 3.39 (ddd, J = 16.3, 7.8, 5.1 Hz, 1H), 2.91–2.78 (m, 2H), 2.34–2.21 (m, 1H), 2.08–1.96 (m, 1H), 1.94–1.72 (m, 1H), 0.78 (dd, J = 6.9, 3.5 Hz, 3H), 0.72 (t, J = 6.7 Hz, 3H). ^13^C NMR (126 MHz, CDCl_3_) δ 204.2, 170.4, 170.3, 163.3, 130.6, 129.6, 114.4, 64.1, 63.9, 61.8, 55.7, 42.3, 42.2, 31.1, 31.1, 19.2, 19.1, 17.1, 17.1.

*(2R)-2-((4-Cyanophenyl)sulfonamido)-3-methyl-N-(2-oxocyclobutyl)butanamide* (**3aa**). Cyclobutanone **3aa** was synthesized from the intermediate described in the general procedure of D-valine sulfonamide para-phenyl substituents following the general procedure of the T3P coupling reactions of N-Ts amino acids with 2-aminocyclobutanone synthon **(±)-2** followed by immediate acid-hydrolysis of the acetal to form the ketone. The crude mixture of **3aa** was purified via column chromatography using ethyl acetate/hexane (60/40) to afford Ts-D-valine cyclobutanone analog **3aa** (23.1 mg, 30%) as a white solid: mp 149.5–151 °C. ^1^H NMR (500 MHz, chloroform-*d*) 7.92–7.84. (m, 2H), 7.79–7.70 (m, 2H), 5.29 (dd, J = 24.2, 8.8 Hz, 1H), 3.47 (ddd, J = 11.3, 8.9, 5.3 Hz, 1H), 2.95–2.85 (m, 1H), 2.85–2.77 (m, 1H), 2.35–2.22 (m, 1H), 1.99–1.89 (m, 1H), 1.70 (dtd, J = 11.1, 9.7, 8.2 Hz, 1H), 0.87–0.79 (m, 6H). ^13^C NMR (126 MHz, chloroform-*d*) δ 203.5, 169.5, 132.9, 127.9, 117.2, 63.8, 61.9, 42.3, 31.8, 19.4, 19.1, 17.3.

*Methyl 4-(N-((2R)-3-methyl-1-oxo-1-((2-oxocyclobutyl)amino)butan-2-yl)sulfamoyl)benzoate* (**3ab**). Cyclobutanone sulfonamide **3ab** was synthesized from the intermediate described in the general procedure of D-valine sulfonamide para-phenyl substituents following the general procedure of the T3P coupling reactions of N-Ts amino acids with 2-aminocyclobutanone synthon **(±)-2** followed by immediate acid-hydrolysis of the acetal to form the ketone. The crude cyclobutanone **3ab** was purified via column chromatography using ethyl acetate/hexane (60/40) to afford Ts-D-valine cyclobutanone analog **3ab** (23.8 mg, 20%) as a white solid: mp 190–191 °C. ^1^H NMR (500 MHz, chloroform-d) δ 8.2 (dq, J = 8.5, 1.9 Hz, 2H), 7.9 (dq, J = 8.5, 2.0 Hz, 2H), 5.3 (s, 1H), 4.9–4.7 (m, 1H), 3.9 (d, J = 2.1 Hz, 3H), 3.6–3.5 (m, 1H), 3.0–2.9 (m, 2H), 2.4–2.3 (m, 1H), 2.1 (hd, J = 6.8, 5.3 Hz, 1H), 1.8 (ddtd, J = 35.8, 11.1, 9.6, 8.1 Hz, 1H), 0.9 (ddd, J = 14.4, 6.8, 3.1 Hz, 6H). ^13^C NMR (126 MHz, chloroform-*d*) δ 203.8, 169.8, 165.6, 143.3, 134.2, 130.4, 130.4, 127.4, 127.3, 63.9, 61.9, 52.7, 42.3, 31.5, 19.3, 19.0, 17.2.

*(2R)-2-((4-hydroxyphenyl)sulfonamido)-3-methyl-N-(2-oxocyclobutyl)butanamide* (**3ac**). Cyclobutanone sulfonamide **3ac** was synthesized from the intermediate described in the general procedure of D-valine sulfonamide para-phenyl substituents following the general procedure of the T3P coupling reactions of N-Ts amino acids with 2-aminocyclobutanone synthon **(±)-2** followed by immediate acid-hydrolysis of the acetal to form the ketone. The crude cyclobutanone **3ac** was purified via column chromatography using ethyl acetate/hexane (60/40) to afford Ts-D-valine cyclobutanone analog **3ac** (11 mg, 14%) as a white solid: mp 139–141 °C. ^1^H NMR (500 MHz, chloroform-*d*) δ 7.66–7.62 (m, 2H), 7.2 (d, *J* = 2.1 Hz, 2H), 6.9–6.8 (m, 1H), 5.0 (dtt, *J* = 10.3, 8.3, 2.1 Hz, 2H), 3.7–3.6 (m, 1H), 3.0–2.9 (m, 1H), 2.9 (t, *J* = 2.0 Hz, 1H), 2.5–2.5 (m, 1H), 2.1–2.0 (m, 1H), 1.2 (s, 6H). ^13^C (126 MHz, chloroform-*d*) 208.27, 173.76, 159.37, 131.28, 120.62, 63.40, 42.76, 31.94, 22.70, 19.28, 14.13.

#### 4.2.5. Synthesis of Cbz Protected Amino Acid Cyclobutanone Analogs **3ad**-**3ag**

Cbz protected amino acids (Phe, Try, Val, 50 mg) were coupled with 2-aminocyclobutanone synthon **(±)-2** following the general T3P coupling reaction procedure. The resulting acetal intermediates were subjected to hydrolysis without further purification. To a solution of crude acetal product (1 eq, concentration = 0.1 M) in THF (40% *v*/*v*), water (20% *v*/*v*) and acetic acid were added, and the reaction mixture was stirred at 60 °C overnight or until deemed complete via HPLC. Upon completion, excess acetic acid was neutralized by adding a solution of saturated Na_2_CO_3_ until a pH of 8 was achieved, and the organic product was extracted with methylene chloride (3 × 3 mL). The combined organic layers were dried over Na_2_SO_4_, and the solvent was evaporated under reduced pressure. The crude mixture was purified via column chromatography to afford the corresponding Cbz-protected amino acid-derived cyclobutanone (**3ad**-**3af**).

*Benzyl ((2S)-1-oxo-1-((2-oxocyclobutyl)amino)-3-phenylpropan-2-yl)carbamate* (**3ad**). The crude mixture of **3ad** was purified via column chromatography eluting with a gradient of ethyl acetate/hexane (20/80) to (30/70). Appropriate fractions were combined and concentrated to produce **3ad** as a white solid (94% HPLC purity) which was then recrystallized from hot methylene chloride to produce white needle-like crystals of compound **3ad** with 97% HPLC purity (30.2 mg, 49%): mp 100–101 °C. ^1^H NMR (500 MHz, CDCl_3_) δ 7.40–7.23 (m, 8H), 7.23–7.17 (m, 2H), 6.63 (NH, d, J = 7.6 Hz, 0.5H), 6.48 (NH, d, J = 7.9 Hz, 0.5H), 5.46 (t, J = 10.6 Hz, 1H), 5.07 (t, J = 3.3 Hz, 2H), 4.96–4.87 (m, 0.5H), 4.78 (qt, J = 7.9, 1.4 Hz, 0.5H), 4.45 (d, J = 7.0 Hz, 1H), 3.09 (d, J = 6.7 Hz, 2H), 2.98–2.83 (m, 2H), 2.38 (dd, J = 10.9, 5.2 Hz, 0.5H), 2.33 (dd, J = 10.3, 4.7 Hz, 0.5H), 2.03 (q, J = 9.6 Hz, 0.5H), 1.90–1.73 (m, 0.5H). ^13^C NMR (126 MHz, CDCl_3_) δ 204.8, 204.8, 170.8, 170.7, 156.0, 136.2, 136.2, 136.1, 129.4, 129.4, 128.8, 128.6, 128.3, 128.0, 127.1, 67.2, 64.0, 63.7, 56.0, 55.8, 42.2, 42.1, 38.6, 38.4, 19.4, 19.1. HRMS (ESI): Calcd for (MH^+^) C_21_H_23_N_2_O_4_: 367.1652, found 367.1646.

*Benzyl ((2R)-1-oxo-1-((2-oxocyclobutyl)amino)-3-phenylpropan-2-yl)carbamate* (**3ae**). The crude mixture of **3ae** was purified via column chromatography eluting with a gradient of ethyl acetate/hexane (20/80) and ethyl acetate/hexane (30/70). Appropriate fractions were combined and concentrated to produce a white solid (93% HPLC purity), which was then subjected to recrystallization. The product was dissolved in a minimal amount of methylene chloride, and hexane was added dropwise until cloudiness was observed. Colorless crystals were formed providing compound **3ae** (16.1 mg, 26%): mp 98–100 °C. ^1^H NMR (500 MHz, CDCl_3_) δ 7.41–7.24 (m, 8H), 7.21 (t, J = 6.0 Hz, 2H), 6.35 (d, J = 7.6 Hz, 0.5H), 6.20 (d, J = 8.0 Hz, 0.5H), 5.32 (s, 0.5H), 5.27 (s, 0.5H), 5.10 (s, 2H), 4.92 (q, J = 9.0 Hz, 0.5H), 4.81 (q, J = 8.7 Hz, 0.5H), 4.42 (q, J = 7.3 Hz, 1H), 3.17–3.11 (m, 1H), 3.06 (td, J = 13.5, 12.9, 7.6 Hz, 1H), 2.99–2.85 (m, 2H), 2.39 (dq, J = 15.8, 9.7 Hz, 1H), 2.06–2.03 (m, 0.5H), 1.88–1.81 (m, 0.5H). ^13^C NMR (126 MHz, CDCl_3_) δ 204.3, 170.7, 170.5, 156.0, 136.0, 136.0, 129.4, 129.4, 128.8, 128.6, 128.3, 128.3, 128.1, 127.2, 67.2, 64.1, 63.8, 56.0, 42.2, 42.1, 38.6, 38.2, 19.4, 19.2. HRMS (ESI): Calcd for (MH^+^) C_21_H_23_N_2_O_4_: 367.1652, found 367.1650.

*Benzyl ((2S)-3-(4-hydroxyphenyl)-1-oxo-1-((2-oxocyclobutyl)amino)propan-2-yl)carbamate* (**3af**). The crude mixture of **3af** was purified via column chromatography eluting with a gradient of ethyl acetate/hexane (20/80) to (30/70). Combined fractions were recrystallized from hot methylene chloride to afford compound **3af** (14.6 mg, 24%) as small needle-like crystals: mp 154–155 °C. ^1^H NMR (500 MHz, acetonitrile-d3) δ 7.42–7.26 (m, 4H), 7.23–7.13 (m, 1H), 7.11–7.04 (m, 2H), 6.77–6.71 (m, 2H), 5.88 (dd, J = 12.9, 8.5 Hz, 1H), 5.08 (d, J = 12.8 Hz, 1H), 4.99 (d, J = 12.7 Hz, 1H), 4.80 (dddd, J = 20.0, 10.0, 5.9, 2.1 Hz, 1H), 4.25 (dtd, J = 17.5, 8.7, 5.3 Hz, 1H), 3.04 (ddd, J = 13.6, 7.8, 5.4 Hz, 1H), 2.96–2.81 (m, 1H), 2.79 (dddd, J = 14.1, 12.0, 8.3, 2.7 Hz, 1H), 2.15–1.91 (m, 1H). ^13^C NMR (126 MHz, CD3CN) δ 206.1, 171.2, 171.1, 155.7, 137.1, 130.4, 128.5, 128.3, 128.2, 127.9, 127.5, 115.1, 66.1, 63.7, 63.6, 56.3, 56.1, 41.4, 41.4, 18.3, 18.1. HRMS (ESI): Calcd for (MNa^+^) C_22_H_21_N_2_O_5_Na: 405.1421, found 405.1420.

*Benzyl ((2S)-3-methyl-1-oxo-1-((2-oxocyclobutyl)amino)butan-2-yl)carbamate* (**3ag**). The crude mixture of **3ag** was purified via column chromatography eluting with a gradient of ethyl acetate/hexane (20/80) to (30/70). Appropriate fractions were combined and concentrated to produce a white solid, which was then subjected to recrystallization. The product was dissolved in a minimal amount of methylene chloride, and hexane was added dropwise until cloudiness was observed. Over 24 h, needle-like crystals formed providing compound **3ag** (21.3 mg, 34%): mp 140–144 °C. ^1^H NMR (500 MHz, CDCl_3_) δ 7.38 (s, 3H), 7.43–7.32 (m, 2H), 6.44–6.39 (d, 1H), 5.26 (d, J = 8.9 Hz, 1H), 5.14 (s, 2H), 4.91 (dt, J = 10.1, 7.9 Hz, 1H), 4.01 (dd, J = 8.8, 6.0 Hz, 1H), 2.98 (t, J = 8.8 Hz, 2H), 2.46 (dd, J = 13.3, 5.3 Hz, 1H), 2.18 (h, J = 6.8 Hz, 1H), 2.13–2.06 (m, 1H), 1.00 (d, J = 6.8 Hz, 3H), 0.95 (d, J = 6.8 Hz, 3H). ^13^C NMR (126 MHz, CDCl_3_) δ 204.4, 171.0, 152.2, 136.1, 128.6, 128.3, 128.1, 67.3, 64.2, 60.0, 42.3, 30.8, 29.7, 19.5, 19.2. HRMS (ESI): Calcd for (MH^+^) C_17_H_23_N_2_O_4_: 319.1652, found 319.1641.

#### 4.2.6. General Procedure for Sulfonamide Cyclobutanones (**3ah** and **3ai**)

Acetal **(±)-2** (50.0 mg, 0.298 mmol) was suspended in methylene chloride (0.8 mL) and the reaction was cooled to 0 °C. Triethylamine (82.6 µL, 0.596 mmol) was then added to the solution, followed by dropwise addition of the requisite benzenesulfonyl chloride (87.6 mg, 0.358 mmol) in methylene chloride (0.7 mL). The reaction was stirred at 0 °C for 5 min, then allowed to warm to rt and stirred overnight. Once the reaction was deemed complete via TLC, the organic layer was washed successively with water (2 × 3 mL), 1N HCl (3 mL), and saturated Na_2_CO_3_. Then, the organic layer was dried over sodium sulfate, and the solvent was removed under reduced pressure to afford the crude acetal intermediate. Without further purification, the crude material (97.9 mg) was subjected to hydrolysis by dissolving in a mixture of acetone (2.0 mL), water (0.30 mL), and 1N HCl (0.60 mL) and was then allowed to stir at 40 °C overnight. The reaction was then partitioned between water and methylene chloride, and the organic product was extracted with methylene chloride (2 × 3 mL). The combined organic fractions were dried over Na_2_SO_4_ and concentrated under reduced pressure. The resultant crude product was purified via column chromatography to produce the corresponding sulfonamides. 

*N-(2-Oxocyclobutyl)-4-(trifluoromethyl)benzenesulfonamide* (**3ah**). The crude product of **3ah** was purified via column chromatography eluting with ethyl acetate/hexane (30/70) to afford benzenesulfonamide **3ah** as a clear oil (60.8 mg, 70%), which formed needle-like crystals in ethyl acetate/hexane (10/90). mp 74–76 °C. ^1^H NMR (500 MHz, CDCl_3_) δ 7.97 (d, J = 8.2 Hz, 2H), 7.74 (d, J = 8.3 Hz, 2H), 5.30 (d, J = 8.1 Hz, 1H), 4.73–4.64 (m, 1H), 2.89 (dddd, J = 17.4, 11.3, 9.9, 1.7 Hz, 1H), 2.73 (dddd, J = 17.4, 9.9, 4.2, 2.5 Hz, 1H), 2.41 (tdd, J = 11.1, 10.1, 4.2 Hz, 1H), 1.79 (dtd, J = 11.3, 9.9, 8.4 Hz, 1H). ^13^C NMR (126 MHz, CDCl_3_) δ 201.30, 142.80, 133.71 (q, J C-F = 33.2 Hz), 126.50, 125.36 (q, J C-F = 3.8 Hz), (ortho carbons of aryl-CF_3_ long range F splitting: 123.2, 121.1, 118.9, 76.2, 76.0, 75.7, 64.8, 40.9, 19.9. HRMS (ESI): Calcd for (MH^+^) C_11_H_11_F_3_NO_3_S: 294.04, found 294.0405, Calcd for: (M+Na^+^) C_11_H_11_F_3_NO_3_SNa^+^: 316.0231, found 316.0222.

*N-(2-Oxocyclobutyl)-4-(fluoro)benzenesulfonamide* (**3ai**). The crude product of **3ai** was purified via column chromatography on a Teledyne Isco Rf Flash chromatography unit eluting with a gradient of 100% hexane, 5% EA in hexane, and 10% EA in hexane to afford compound **3ai** as a clear oil (13.6 mg, 23%). ^1^H NMR (500 MHz, CDCl_3_) δ 8.00–7.90 (m, 1H), 7.93–7.85 (m, 1H), 7.27–7.15 (m, 2H), 5.52 (d, J = 8.2 Hz, 1H), 4.72 (dtt, J = 10.3, 8.3, 2.2 Hz, 1H), 2.93 (dddt, J = 17.4, 11.3, 9.8, 1.7 Hz, 1H), 2.77 (dddd, J = 17.3, 9.9, 4.2, 2.5 Hz, 1H), 2.47–2.36 (m, 1H), 1.83 (dtd, J = 11.1, 9.9, 8.5 Hz, 1H). ^13^C NMR (126 MHz, CDCl_3_) δ 203.0, 165.23 (d, J C-F = 255.3 Hz), 136.3 (d, J C-F = 3.6 Hz), 129.8 (d, J C-F = 9.4 Hz), 116.5 (d, J C-F = 22.9 Hz), 65.9, 41.9, 20.8. HRMS (ESI) calcd for MNa^+^ C_10_H1_0_FNO_3_SNa^+^: 266.0258, found (M+Na^+^) 266.0250.

*(S)-N-((1-hydroxycyclopropyl)methyl)-2-((4-methylphenyl)sulfonamido)-3-phenylpropanamide* (**4a**). Tosyl-L-phenylalanine (50 mg, 0.129 mmol) was reacted with 1-(aminomethyl)cyclopropanol (14.4 µL, 0.155 mmol) following the general T3P coupling procedure and stirred at 50 °C for 4 days. The crude mixture of **4a** was purified via column chromatography on a Teledyne Isco Rf Flash chromatography unit eluting with a gradient of ethyl acetate/hexane (20/80) and ethyl acetate/hexane (30/70). Appropriate fractions were combined and concentrated to produce a white solid, which was then subjected to recrystallization. The product was dissolved in a minimal amount of hot methylene chloride, and hexane was added dropwise until cloudiness was observed. While cooling, needle-like crystals formed providing compound **4a** (13.6 mg, 27%). mp 131–133 °C. ^1^H NMR (500 MHz, CDCl_3_) δ 7.51 (dd, J = 8.1, 2.4 Hz, 2H), 7.25 (d, J = 13.9 Hz, 1H), 7.21 (dd, J = 12.2, 7.2 Hz, 5H), 6.94 (d, J = 7.1 Hz, 2H), 6.75 (NH, d, J = 6.3 Hz, 1H), 4.96 (NH, d, J = 5.7 Hz, 1H), 3.86–3.80 (m, 1H), 3.39 (ddd, J = 14.3, 6.8, 2.4 Hz, 1H), 3.30–3.22 (m, 1H), 3.20 (OH, s, 1H), 2.95 (ttd, J = 14.1, 10.3, 8.5, 4.4 Hz, 2H), 2.43 (d, J = 2.4 Hz, 3H), 0.79 (s, 2H), 0.64–0.56 (m, 1H), 0.56–0.50 (m, 1H). ^13^C NMR (126 MHz, CDCl_3_) δ 171.1, 144.2, 135.2, 135.0, 129.9, 129.1, 129.1, 127.4, 127.3, 58.2, 55.4, 47.7, 38.3, 21.6, 12.8, 12.5.

*(S)-N-(2-hydroxy-2-methylpropyl)-2-((4-methylphenyl)sulfonamido)-3-phenylpropanamide* (**4b**). Tosyl-L-phenylalanine (50 mg, 0.129 mmol) was reacted with dimethylethanolamine (14.4 µL, 0.155 mmol) following the general T3P coupling procedure and stirred at 50–60 °C for 6 days until completion. The crude product (oil) of **4b** was crystallized from hot EA/hexane = 20/80. When the EA/hexane solution was cooled to rt, the product became an oil. Therefore, it is important to maintain the temperature of the solution to stay between 50–60 °C to allow a slow crystallization of the final product. Recrystallization provided compound **4b** (29 mg, 58%) as clear needle-like crystals: mp 131–133 °C. ^1^H NMR (500 MHz, CDCl_3_) δ 7.53–7.47 (m, 2H), 7.23–7.14 (m, 5H), 6.96–6.90 (m, 2H), 6.74 (s, 1H), 4.93 (d, J = 6.2 Hz, 1H), 3.84 (dt, J = 7.8, 6.1 Hz, 1H), 3.29 (dd, J = 13.7, 6.9 Hz, 1H), 3.12 (dd, J = 13.7, 5.5 Hz, 1H), 2.97 (dd, J = 14.0, 5.9 Hz, 1H), 2.91 (dd, J = 14.0, 7.7 Hz, 1H), 2.43 (s, 3H), 1.16 (d, J = 2.2 Hz, 6H). ^13^C NMR (126 MHz, CDCl_3_) δ 170.9, 144.1, 135.2, 135.2, 129.9, 129.1, 129.0, 127.4, 127.2, 70.9, 58.1, 50.3, 38.3, 27.12, 27.1, 21.6. HRMS (ESI): Calcd for (MH^+^) C_20_H_27_N_2_O_4_S: 391.1686, found 391.1680.

### 4.3. Molecular Docking Protocol

Recent docking experiments utilized the new 1.30 Å resolution DapE structure from *Neisseria meningitidis* (PDB ID: 5UEJ) as the docking receptor due to the high quality of refinement at the dimerization loops that is critical in substrate binding. Ligand molecular models were generated using Molecular Operating Environment (MOE) computational suite’s Builder utility. Protonation states of ligands were optimized at pH 7.4 and relaxed through energy minimization in the gas phase using the MMFF94X1 force field. Ligand models were then sampled via a conformational search that generates energetically reasonable 3D atomic configurations of ligands with or without geometric constraints and analyzed for potential energy local minima. Depending on the ligand, a set of stable conformers, or the most stable conformer, was identified as the preferred docking ligand model. Then, the X-ray crystal structure of *Nm*DapE (PDB ID: 5UEJ) was loaded into MOE and prepared for docking using MOE’s Structure Preparation utility. The hydrogen-bonding network of the docking receptor was further optimized at physiological conditions (pH 7.4 and T = 310 K) by automatically sampling different tautomer/protomer states using Protonate3D, which calculates optimal protonation states, including titration, rotamer, and “flips” using a large-scale combinatorial search. The substrate-binding pocket of chain A/B, where the di-Zn(II) metal center is located, was analyzed using MOE’s Site Finder utility and populated with inactivated dummy atoms that define the docking location. Following preparation of the *Nm*DapE docking receptor model, an induced-fit molecular docking using the previously identified ligand conformers (a database or a single conformer) was carried out with solvent atoms inactivated at the docking site. The docking site was specified by the dummy atoms populating the substrate-binding site of the docking receptor. The Alpha triangle placement with Affinity dG scoring generated 1000 data points, which were further refined using the induced fit method with GBVI/WSA dG scoring to obtain the top 300 docking results. These calculations were performed using the Amber12:EHT3 force field. Then, the ligand poses were analyzed and selected based on the docking score and the observed desired interactions. For DapE inhibitors, the top ligand docking poses that coordinate to the active site zinc ions, along with other favorable interactions (Coulombic and hydrophobic), were selected.

### 4.4. DapE Assay

The inhibition of *Hi*DapE by potential inhibitors was assessed using the previously developed ninhydrin assay [27] with slight modifications. It was performed in a PCR Thermal Cycler System. All experiments were performed in triplicate in 50 mM HEPES buffer at pH 7.5. Reaction volumes were 100 µL of 2 mM *N*^6^-Methyl-L,L-SDAP and 8 nM *Hi*DapE. Ninhydrin was purchased as a 2% solution in 100% DMSO/lithium acetate buffer at pH 5.2. The inhibitor, followed by *Hi*DapE, was added to a 50 mM HEPES (pH 7.5) buffered solution at 30 °C, and the mixture was incubated at 30 °C for 10 min. *N*^6^-Methyl-L,L-SDAP was added, and the reaction mixture was incubated at 30 °C for 10 min, then heated to 99 °C for 1 min, and further cooled to 0 °C for 1 min. The 2% ninhydrin solution (50 µL) was added, and the mixture was thoroughly mixed. The reaction mixture was heated to 80 °C for 15 min, then cooled to 0 °C for 1 min. The absorbance of an 80 μL aliquot was recorded at 570 nm on a BioTek Synergy 2 microplate reader. All the inhibition assays of *Hi*DapE were performed in triplicate and the IC_50_ values were obtained by fitting the data following the modified Hill equation: V = V_0_ + (V_max_ − V_0_) XnH/(XnH0.5 + XnH) using the graphing suite Origin 9.1 with the Levenberg–Marquardt non-linear least-squares algorithm. The velocity in the absence of the substrate is V; the velocity at saturating concentrations of the substrate or with no inhibitor for the inhibition assay is V_max_; X is the concentration of the substrate or inhibitor; and X0.5 is the substrate (S0.5) and inhibitor (I0.5) concentration at 50% maximum velocity and at 50% inhibition, respectively. The Hill coefficient is represented as nH.

### 4.5. Thermal Shift

*H. influenzae* DapE was used at a final concentration of 200 nM and SYPRO Orange was used at a final concentration of 10×. It was performed in a Step One Real-Time PCR System (Applied Biosystems, Waltham, MA, USA). The experiment was carried out in 10 µL triplicates in 50 mM HEPES buffer at pH 7.5, nanopure water, and the inhibitor concentrations were based on the IC_50_ values of the inhibitor, **3y**. Sample solutions were dispensed into a 96-well optical reaction plate (Thermo Fisher Scientific, Waltham, WA, USA) and the plate was sealed with an optical PCR plate sheet (Thermo Fisher Scientific). The temperature was continuously increased 0.05 °C/s for 2 min at 25 °C then increased 0.05 °C/s for 2 min at 99 °C and was scanned every 0.4 °C. Melting curves were obtained from the negative derivative and exported from the instrument into Excel. The negative melting temperatures were smoothed and differentiated to the negative third derivative. The T_m_ data were plotted against the log(concentration of **3y**) with GraphPad Prism using a derived Van’t Hoff equation [39] to find the K_i_ value.

### 4.6. Equilibria Studies

Cyclobutanone **3y** was massed to 2–3 mg and the exact mass was recorded. Compound **3y** was then dissolved in the appropriate amount of deuterated solvent (DMSO-d6 or CDCl_3_) and vortexed. The appropriate amount of deuterium oxide (0%, 2%, 10%, 20%, or 30%) was added to the sample and the sample was vortexed. The sample was inserted into the Bruker 500 MHz NMR and data were collected for 64 scans. The raw data were analyzed with the Mnova NMR program, and the K_eq_ was calculated via the method described by the Wiberg group [32,40].

## Figures and Tables

**Table 1:** Cyclobutanone derivatives and analogs.

Cmpd ID	R Group for Structure 3	LogP	mp (°C)	DapE % Inhib at 100 µM	DapE IC_50_ (µM)
**3a**	o-tolyl-CO	2.0	100–102	16.8	---
**3b**	m-tolyl-CO	2.0	94–96	37.8	---
**3c**	p-tolyl-CO	2.0	149–150	58.1	---
**3d**	o-chlorophenyl-CO	2.2	102–103	33.6	---
**3e**	m-chlorophenyl-CO	2.2	64–66	45.8	---
**3f**	p-bromophenyl-CO	2.4	104–105	25.2	---
**3g**	3-Cl-4-MeO-phenyl-CO	2.1	134–136	29.1	---
**3h**	2-chloro-4,5-dimethoxyphenyl-CO	1.8	123–125	24.4	---
**3i**	2-hydroxyphenyl-CO	1.6	149–151	28.9	---
**3j**	2-hydroxy-4-methoxyphenyl-CO	1.5	131–133	73	66.4 ± 2.9
**3k**	3,4,5-trimethoxyphenyl-CO	1.0	144–146	93.7	39.6 ± 2.9
**3l**	2-pyridyl-CO	0.4	97–99	25.6	---
**3m**	3-pyridyl-CO	0.3	oil	24.9	---
**3n**	2-chloro-3-pyridyl-CO	1.1	80–82	<5.0	---
**3o**	3-bromo-5-pyridyl-CO	1.1	114–115	34.6	---
**3p**	4-pyridyl-CO	0.3	119–121	<5.0	---
**3q**	3-quinoline-CO	1.7	155–156	48	---
**3r**	8-quinoline-CO	1.7	89–91	<5.0	---
**3s**	3-methoxyphenyl-CH_2_CO	1.3	104–105	27	---
**3t**	4-bromophenyl-CH_2_CO	2.2	124–127	59	---
**3u**	2-benzimidazole-CH_2_CH_2_-CO	0.9	179–181	84.7	52.4 ± 7.4
**3v**	Tos-L-Phe	2.6	177–180	92.8	45.7 ± 1.5
**3w**	Tos-D-Phe	2.6	190–192	55.0	---
**3x**	Tos-L-Trp	2.7	200–201	19.6	---
**3y**	p-methoxyphenyl-SO_2_-D-Val	1.3	179–181	99.7	23.1 ± 0.9
**3z**	p-methoxyphenyl-SO_2_-L-Val	1.3	179–181	30.7	---
**3aa**	p-cyanophenyl-SO_2_-D-Val	1.39	150–151	5.6	---
**3ab**	p-methylbenzoate-SO_2_-D-Val	1.12	190–191	10.7	---
**3ac**	p-hydroxyphenyl-SO_2_-D-Val	0.8	139–141	44.4	---
**3ad**	Cbz-L-Phe	2.9	100–101	96.2	69.8 ± 1.4
**3ae**	Cbz-D-Phe	2.9	98–100	97.9	55.4 ± 5.5
**3af**	Cbz-L-Tyr	2.5	154–155	15.5	---
**3ag**	Cbz-L-Val	2.1	140–144	53.9	---
**3ah**	4-trifluoromethylphenyl-SO_2_	2.0	74–76	22.6	---
**3ai**	4-fluorophenyl-SO_2_	1.2	oil	72.9	53.3 ± 5.4
**4a**	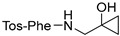	2.2	131–132	36.4	---
**4b**	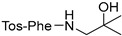	2.6	131–133	19.1	---

**Table 2:** Thermal shift assay data.

200 nM *Hi*DapE + 10× SYPRO + 3y	T_m_1 (°C)	K_i_ (µM)	T_m_2 (°C)
no inhibitor	49.6 +/− 0.24		77.3 +/− 0.37
1 μM	49.6 +/− 0.18		76.5 +/− 0.54
5 μM	49.0 +/− 0.30		77.3 +/− 0.20
10 μM	50.0 +/− 0.34	10.2 +/− 0.26	77.3 +/− 0.23
20 μM	51.2 +/− 0.13		77.3 +/− 0.51
30 μM	52.1 +/− 0.5		75.1 +/− 0.17
40 μM	52.7 +/− 0.37		75.2 +/− 0.11
50 μM	53.4 +/− 0.23		74.7 +/− 0.40
60 μM	54.1 +/− 0.35		75.1 +/− 0.23
75 μM	54.6 +/− 0.42		75.7 +/− 0.29

**Table 3:** D_2_O hydrate:cyclobutanone equilibrium studies of cyclobutanone **3y**.

Compound + D_2_O	K_eq_	% Hydrate
Cyclobutanone + D_2_O	2.8 × 10^−3^	2.0%
**3y** in CDCl_3_ + 30% D_2_O	0.082	7.33%
**3y** in DMSO-d6 + 2% D_2_O	0.58	35.4%
**3y** in DMSO-d6 + 10% D_2_O	0.61	38.8%
**3y** in DMSO-d6 + 20% D_2_O	0.68	40.0%

## Data Availability

Data is contained within the article and Supplementary Material. Samples of the compounds described may be available from the authors.

## Supplementary Materials

The following supporting information can be downloaded at: https://www.mdpi.com/article/10.3390/ijms25021339/s1.
