# Supplementary material for: Cyclobutanone Inhibitors of Diaminopimelate Desuccinylase (DapE) as Potential New Antibiotics
Source: Int J Mol Sci. 2024 Jan 22;25(2):1339. doi: 10.3390/ijms25021339 (PMC10815964; doi:10.3390/ijms25021339)
Supplement: Supplementary file 1 [file ijms-25-01339-s001.zip › ijms-2788624-supplementary.pdf]

Cyclobutanone Inhibitors of Diaminopimelate Desuccinylase (DapE) as Potential Antibiotics

Thahani S. Habeeb Mohammad, Emma H. Kelley, Cory T. Reidl, Katherine Konczak, Megan Beulke, Janielle Javier, Ken Olsen, and Daniel P. Becker*

Department of Chemistry and Biochemistry, 1032 West Sheridan Road, Loyola University Chicago, Chicago, IL 60660, USA

Table of Contents

Figure # Item Description	Pg #
**Figure S1.** ^{1}H NMR of 2-methyl-*N*-(2-oxocyclobutyl)benzamide (**3a**).	S3
**Figure S2.** ^{13}C NMR of 2-methyl-*N*-(2-oxocyclobutyl)benzamide (**3a**).	S4
**Figure S3.** ^{1}H NMR of 3-methyl-*N*-(2-oxocyclobutyl)benzamide (**3b**).	S5
**Figure S4.** ^{13}C NMR of 3-methyl-*N*-(2-oxocyclobutyl)benzamide (**3b**).	S6
**Figure S5.** ^{1}H NMR of 4-methyl-*N*-(2-oxocyclobutyl)benzamide (**3c**).	S7
**Figure S6.** ^{13}C NMR of 4-methyl-*N*-(2-oxocyclobutyl)benzamide (**3c**).	S8
**Figure S7.** ^{1}H NMR of 2-chloro-*N*-(2-oxocyclobutyl)benzamide **(3d).**	S9
**Figure S8.** ^{13}C NMR of 2-chloro-*N*-(2-oxocyclobutyl)benzamide (**3d**).	S10
**Figure S9.** ^{1}H NMR of 3-chloro-*N*-(2-oxocyclobutyl)benzamide (**3e**).	S11
**Figure S10.** ^{13}C NMR of 3-chloro-*N*-(2-oxocyclobutyl)benzamide (**3e**).	S12
**Figure S11.** ^{1}H NMR of 4-bromo-*N*-(2-oxocyclobutyl)benzamide **(3f).**	S13
**Figure S12.** ^{13}C spectrum of 4-bromo-*N*-(2-oxocyclobutyl)benzamide (**3f).**	S14
**Figure S13.** ^{1}H NMR of 3-chloro-4-methoxy-*N*-(2-oxocyclobutyl)benzamide (**3g**).	S15
**Figure S14.** ^{13}C spectrum of 3-chloro-4-methoxy-*N*-(2-oxocyclobutyl)benzamide (**3g**).	S16
**Figure S15.** ^{1}H NMR of 2-chloro-4,5-dimethoxy-*N*-(2-oxocyclobutyl)benzamide (**3h**).	S17
**Figure S16** ^{13}C spectrum of 2-chloro-4,5-dimethoxy-*N*-(2-oxocyclobutyl)benzamide (**3h**).	S18
**Figure S17.** ^{1}H NMR of 2-hydroxy-*N*-(2-oxocyclobutyl)benzamide (**3i**)	S19
**Figure S18.** ^{13}C spectrum of 2-hydroxy-*N*-(2-oxocyclobutyl)benzamide (**3i**)	S20
**Figure S19.** ^{1}H spectrum of 2-hydroxy-4-methoxy-*N*-(2-oxocyclobutyl)benzamide (**3j**).	S21
**Figure S20.** ^{13}C spectrum of 2-hydroxy-4-methoxy-*N*-(2-oxocyclobutyl)benzamide (**3j**).	S22
**Figure S21.** ^{1}H NMR of 3,4,5-trimethoxy-*N*-(2-oxocyclobutyl)benzamide (**3k**).	S23
**Figure S22.** ^{13}C spectrum of 3,4,5-trimethoxy-*N*-(2-oxocyclobutyl)benzamide (**3k**).	S24
**Figure S23.** ^{1}H NMR of *N*-(2-oxocyclobutyl)picolinamide (**3l**).	S25
**Figure S24.** ^{13}C spectrum of *N*-(2-oxocyclobutyl)picolinamide (**3l**).	S26
**Figure S25.** ^{1}H NMR of *N*-(2-oxocyclobutyl)nicotinamide (**3m**).	S27
**Figure S26.** ^{13}C spectrum of *N*-(2-oxocyclobutyl)nicotinamide (**3m**).	S28
**Figure S27.** ^{1}H NMR of 2-chloro-*N*-(2-oxocyclobutyl)nicotinamide (**3n**).	S29
**Figure S28.** ^{13}C spectrum of 2-chloro-*N*-(2-oxocyclobutyl)nicotinamide (**3n**).	S30
**Figure S29.** ^{1}H NMR of 5-bromo-*N*-(2-oxocyclobutyl)nicotinamide (**3o**).	S31
**Figure S30.** ^{13}C spectrum of 5-bromo-*N*-(2-oxocyclobutyl)nicotinamide (**3o**).	S32
**Figure S31.** ^{1}H NMR of *N*-(2-oxocyclobutyl)isonicotinamide (**3p**).	S33
**Figure S32.** ^{13}C spectrum of *N*-(2-oxocyclobutyl)isonicotinamide (**3p**).	S34
**Figure S33.** ^{1}H NMR of *N*-(2-oxocyclobutyl)quinoline-3-carboxamide (**3q**).	S35
**Figure S34.** ^{13}C spectrum of *N*-(2-oxocyclobutyl)quinoline-3-carboxamide (**3q**).	S36
**Figure S35.** ^{1}H NMR of *N*-(2-oxocyclobutyl)quinoline-8-carboxamide (**3r**).	S37
**Figure S36.** ^{13}C spectrum of *N*-(2-oxocyclobutyl)quinoline-8-carboxamide (**3r**).	S38
**Figure S37.** ^{1}H NMR of 2-(3-methoxyphenyl)-*N*-(2-oxocyclobutyl)acetamide (**3s**).	S39
**Figure S38.** ^{13}C spectrum of 2-(3-methoxyphenyl)-*N*-(2-oxocyclobutyl)acetamide (**3s**).	S40
**Figure S39.** ^{1}H NMR of 2-(4-bromophenyl)-*N*-(2-oxocyclobutyl)acetamide (**3t**).	S41
**Figure S40.** ^{13}C spectrum of 2-(4-bromophenyl)-*N*-(2-oxocyclobutyl)acetamide (**3t**).	S42
**Figure S41.** ^{1}H NMR of 3-(*1H*-benzo[*d*]imidazol-2-yl)-*N*-(2-oxocyclobutyl)propenamide (**3u).**	S43
**Figure S42.** ^{13}C spectrum of 3-(*1H*-benzo[*d*]imidazol-2-yl)-*N*-(2-oxocyclobutyl)propenamide (**3u).**	S44
**Figure S43.** ^{1}H NMR of (*2S*)-2-((4-methylphenyl)sulfonamido)-*N*-(2-oxocyclobutyl)-3-phenylpropanamide (**3v**).	S45
**Figure S44.** ^{1}H NMR of (*2R*)-2-((4-methylphenyl)sulfonamido)-*N*-(2-oxocyclobutyl)-3-phenylpropanamide (**3w**).	S46
**Figure S45.** ^{13}C NMR of (*2R*)-2-((4-methylphenyl)sulfonamido)-*N*-(2-oxocyclobutyl)-3-phenylpropanamide (**3w**).	S47
**Figure S46.** ^{1}H NMR of (*2S*)-3-(1*H*-indol-3-yl)-2-((4-methylphenyl)sulfonamido)-*N*-(2-oxocyclobutyl)propanamide (**3x**).	S48
**Figure S47.** ^{13}C NMR of (*2S*)-3-(1*H*-indol-3-yl)-2-((4-methylphenyl)sulfonamido)-*N*-(2-oxocyclobutyl)propanamide (**3x**).	S49

Figure S48. ^{1}H NMR of (*2R*)-2-((4-methoxyphenyl)sulfonamido)-3-methyl-*N*-(2-oxocyclobutyl)butanamide (**3y**). S50
Figure S49. ^{13}C NMR of (*2R*)-2-((4-methoxyphenyl)sulfonamido)-3-methyl-*N*-(2-oxocyclobutyl)butanamide (**3y**). S51
Figure S50. ^{1}H NMR of (*2S*)-2-((4-methoxyphenyl)sulfonamido)-3-methyl-*N*-(2-oxocyclobutyl)butanamide (**3z**). S52
Figure S51. ^{13}C NMR of (*2S*)-2-((4-methoxyphenyl)sulfonamido)-3-methyl-*N*-(2-oxocyclobutyl)butanamide (**3z**). S53
Figure S52. ^{1}H NMR (500 MHz $CDCl_3$) of (2R)-2-((4-cyanophenyl)sulfonamido)-3-methyl-N-(2-oxocyclobutyl)butanamide (**3aa**). S54
Figure S53. ^{13}C NMR (126 MHz, $CDCl_3$) of (2R)-2-((4-cyanophenyl)sulfonamido)-3-methyl-N-(2-oxocyclobutyl)butanamide (**3aa**). S55
Figure S54. ^{1}H NMR (500 MHz $CDCl_3$) of methyl 4-(N-((2R)-3-methyl-1-oxo-1-((2-oxocyclobutyl)amino)butan-2-yl)sulfamoyl)benzoate (**3ab**). S56
Figure S55. ^{13}C NMR (126 MHz, $CDCl_3$) of methyl 4-(N-((2R)-3-methyl-1-oxo-1-((2-oxocyclobutyl)amino)butan-2-yl)sulfamoyl)benzoate (**3ab**). S57
Figure S56. ^{1}H NMR (500 MHz $CDCl_3$) of (2R)-2-((4-hydroxyphenyl)sulfonamido)-3-methyl-N-(2-oxocyclobutyl)butanamide (**3ac**). S58
Figure S57. ^{13}C NMR (126 MHz, $CDCl_3$) of (2R)-2-((4-hydroxyphenyl)sulfonamido)-3-methyl-N-(2-oxocyclobutyl)butanamide (**3ac**). S59
Figure S58. ^{1}H NMR of benzyl ((*2S*)-1-oxo-1-((2-oxocyclobutyl)amino)-3-phenylpropan-2-yl)carbamate (**3ad**). S60
Figure S59. ^{13}C spectrum of benzyl ((*2S*)-1-oxo-1-((2-oxocyclobutyl)amino)-3-phenylpropan-2-yl)carbamate (**3ad**). S61
Figure S60. ^{1}H NMR of benzyl ((*2R*)-1-oxo-1-((2-oxocyclobutyl)amino)-3-phenylpropan-2-yl)carbamate (**3ae**). S62
Figure S61. ^{13}C spectrum of benzyl ((*2R*)-1-oxo-1-((2-oxocyclobutyl)amino)-3-phenylpropan-2-yl)carbamate (**3ae**). S63
Figure S62. ^{1}H NMR of benzyl ((*2S*)-3-(4-hydroxyphenyl)-1-oxo-1-((2-oxocyclobutyl)amino)propan-2-yl)carbamate (**3af**). S64
Figure S63. ^{13}C spectrum of benzyl ((*2S*)-3-(4-hydroxyphenyl)-1-oxo-1-((2-oxocyclobutyl)amino)propan-2-yl)carbamate (**3af**). S65
Figure S64. ^{1}H NMR of benzyl ((*2S*)-3-methyl-1-oxo-1-((2-oxocyclobutyl)amino)butan-2-yl)carbamate (**3ag**). S66
Figure S65. ^{13}C spectrum of benzyl ((*2S*)-3-methyl-1-oxo-1-((2-oxocyclobutyl)amino)butan-2-yl)carbamate (**3ag**). S67
Figure S66. ^{1}H spectrum of *N*-(2-oxocyclobutyl)-4-(trifluoromethyl)benzenesulfonamide (**3ah**). S68
Figure S67. ^{13}C spectrum of *N*-(2-oxocyclobutyl)-4-(trifluoromethyl)benzenesulfonamide (**3ah**). S69
Figure S68. ^{1}H NMR of *N*-(2-oxocyclobutyl)-4-(fluoro)benzenesulfonamide (**3ai**). S70
Figure S69. ^{13}C NMR of *N*-(2-oxocyclobutyl)-4-(fluoro)benzenesulfonamide (**3ai**). S71
Figure S70. ^{1}H NMR of (S)-*N*-((1-hydroxycyclopropyl)methyl)-2-((4-methylphenyl)sulfonamido)-3-phenylpropanamide (**4a**). S72
Figure S71. ^{13}C NMR of (S)-*N*-((1-hydroxycyclopropyl)methyl)-2-((4-methylphenyl)sulfonamido)-3-phenylpropanamide (**4a**). S73
Figure S72. ^{1}H NMR of (S)-*N*-(2-hydroxy-2-methylpropyl)-2-((4-methylphenyl)sulfonamido)-3-phenylpropanamide (**4b**). S74
Figure S73. ^{13}C NMR of (S)-*N*-(2-hydroxy-2-methylpropyl)-2-((4-methylphenyl)sulfonamido)-3-phenylpropanamide (**4b**). S75
Figure S74. IC_{50} plot of 2-hydroxy-4-methoxy-*N*-(2-oxocyclobutyl)benzamide (**3j**). S76
Figure S75. IC_{50} plot of 3,4,5-trimethoxy-*N*-(2-oxocyclobutyl)benzamide (**3k**). S77
Figure S76. IC_{50} plot of 3-(*1H*-benzo[*d*]imidazol-2-yl)-*N*-(2-oxocyclobutyl)propenamide (**3u).** S78
Figure S77. IC_{50} plot of (*2S*)-2-((4-methylphenyl)sulfonamido)-*N*-(2-oxocyclobutyl)-3-phenylpropanamide (**3v**). S79
Figure S78. IC_{50} plot of (*2R*)-2-((4-methoxyphenyl)sulfonamido)-3-methyl-*N*-(2-oxocyclobutyl)butanamide (**3y**). S80
Figure S79. IC_{50} plot of benzyl ((*2S*)-1-oxo-1-((2-oxocyclobutyl)amino)-3-phenylpropan-2-yl)carbamate (**3ad**). S81
Figure S80. IC_{50} plot of benzyl ((*2R*)-1-oxo-1-((2-oxocyclobutyl)amino)-3-phenylpropan-2-yl)carbamate (**3ae**). S82
Figure S81. IC_{50} plot of 4-fluoro-*N*-(2-oxocyclobutyl)benzenesulfonamide (**3ah**). S83
Figure S82. K_i Graph of T_m vs. log [**3y** (μM)]. S84
Figure S83. ^{1}H NMR (500 MHz DMSO-d6) of (*2R*)-2-((4-methoxyphenyl)sulfonamido)-3-methyl-*N*-(2-oxocyclobutyl)butanamide (**3y**). S85
Figure S84. ^{1}H NMR (500 MHz DMSO-d6 + 2% D_2O) of (*2R*)-2-((4-methoxyphenyl)sulfonamido)-3-methyl-*N*-(2-oxocyclobutyl)butanamide (**3y**). S86
Figure S85. ^{1}H NMR (500 MHz DMSO-d6 + 10% D_2O) of (*2R*)-2-((4-methoxyphenyl)sulfonamido)-3-methyl- *N*-(2-oxocyclobutyl)butanamide (**3y**). S87
Figure S86. ^{1}H NMR (500 MHz DMSO-d6 + 20% D_2O) of (*2R*)-2-((4-methoxyphenyl)sulfonamido)-3-methyl- *N*-(2-oxocyclobutyl)butanamide (**3y**). S88
Figure S87. ^{1}H NMR (500 MHz 70% $CDCl_3$ +30% D_2O) of (*2R*)-2-((4-methoxyphenyl)sulfonamido)-3-methyl- *N*-(2-oxocyclobutyl)butanamide (**3y**). S89

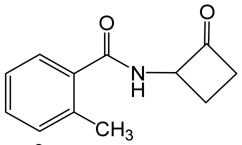

3a

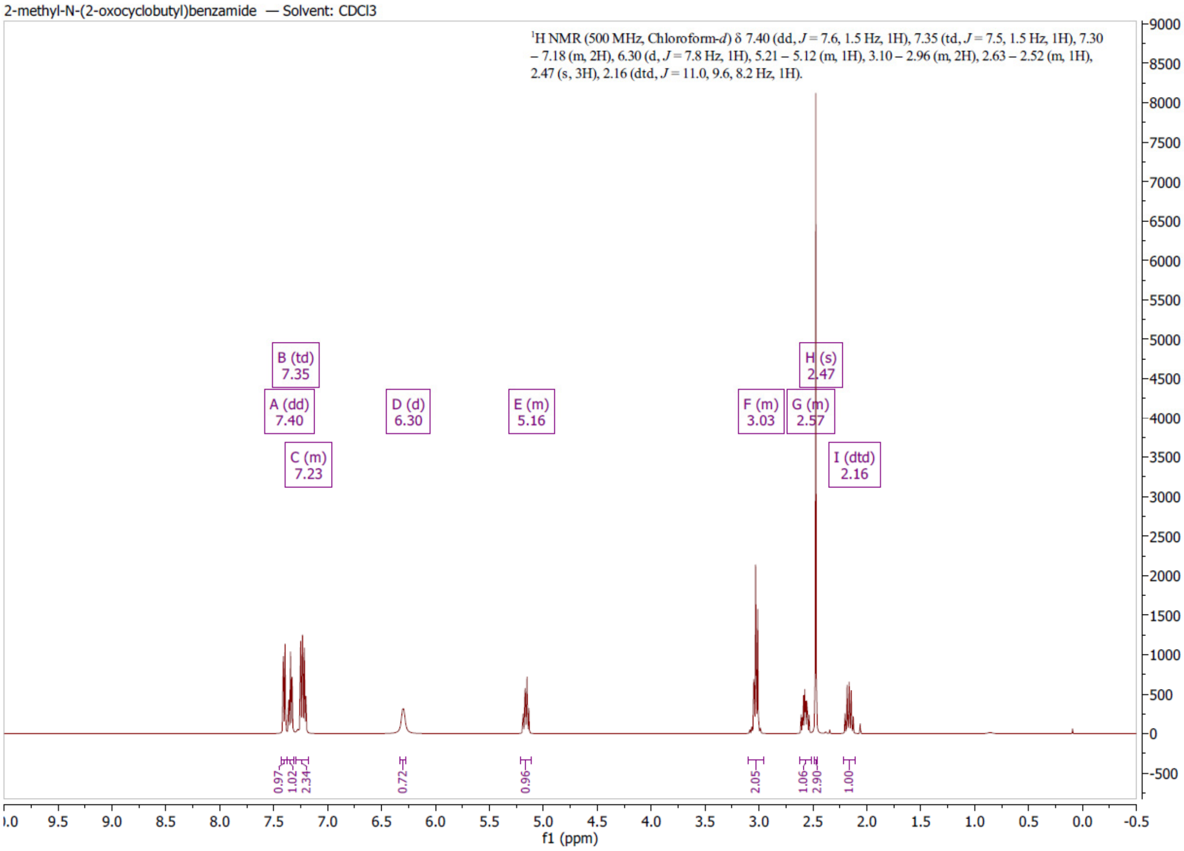

Figure S1. ^{1}H NMR (500 MHz $CDCl_3$) of 2-methyl-*N*-(2-oxocyclobutyl)benzamide (**3a**).

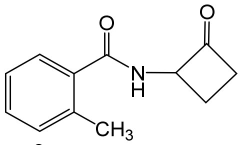

3a

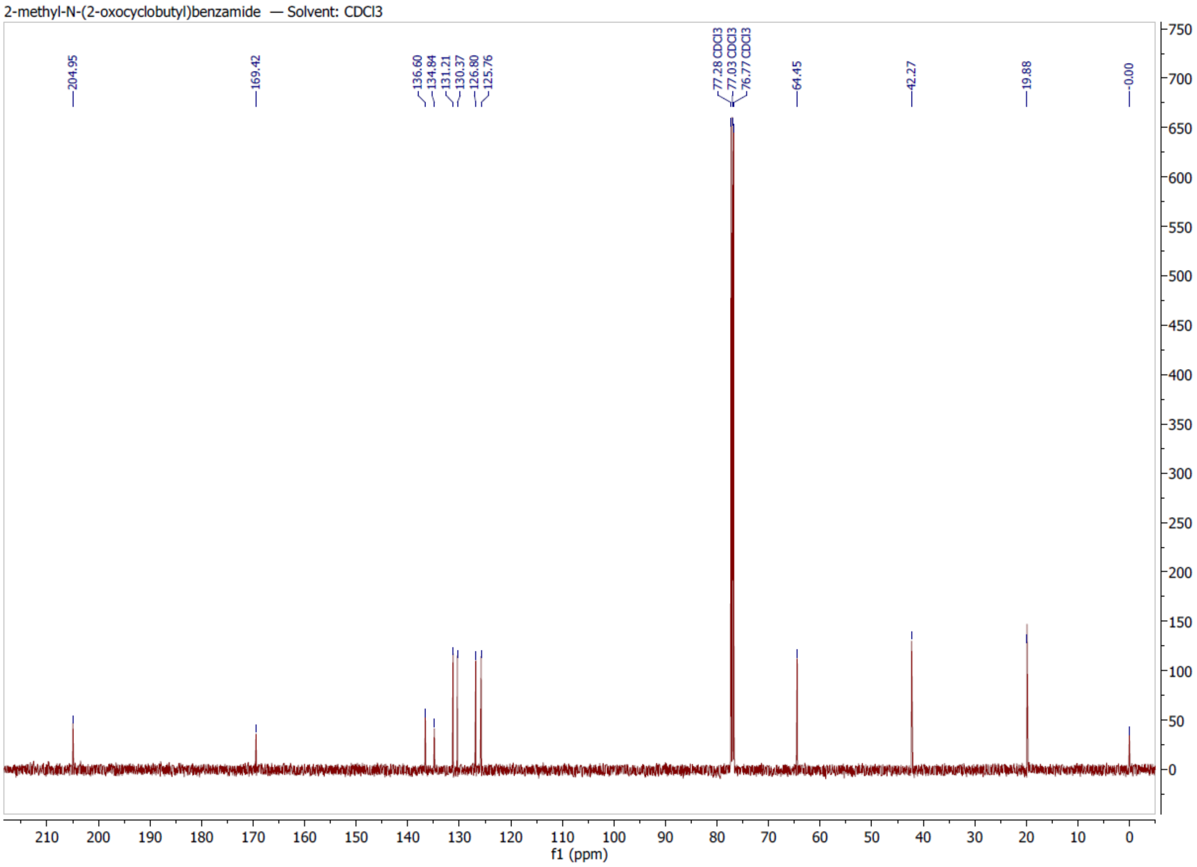

Figure S2. ^{13}C NMR (126 MHz, $CDCl_3$) of 2-methyl-*N*-(2-oxocyclobutyl)benzamide (**3a**).

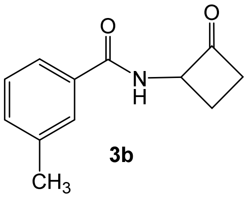

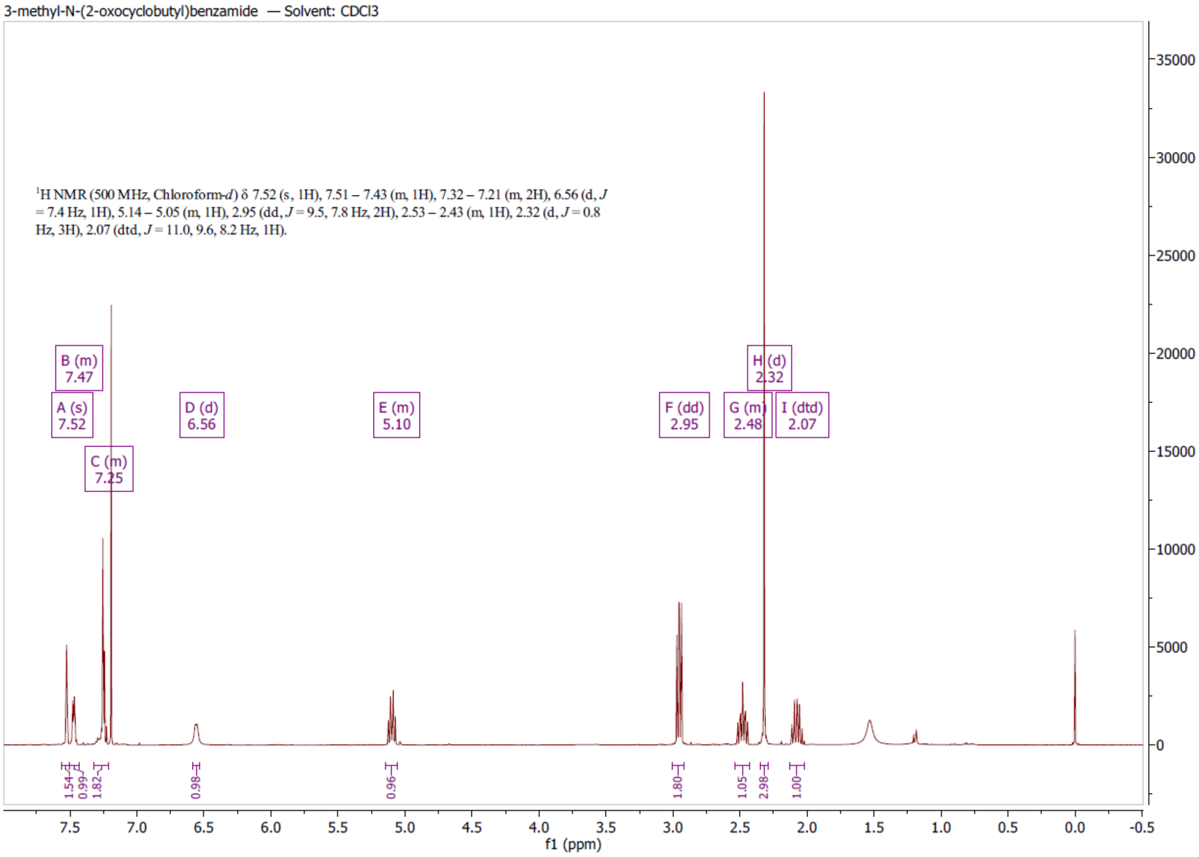

Figure S3. ^{1}H NMR (500 MHz $CDCl_3$) of 3-methyl-*N*-(2-oxocyclobutyl)benzamide (**3b**).

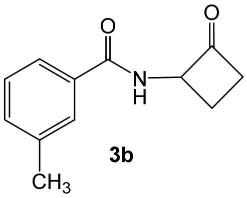

3b

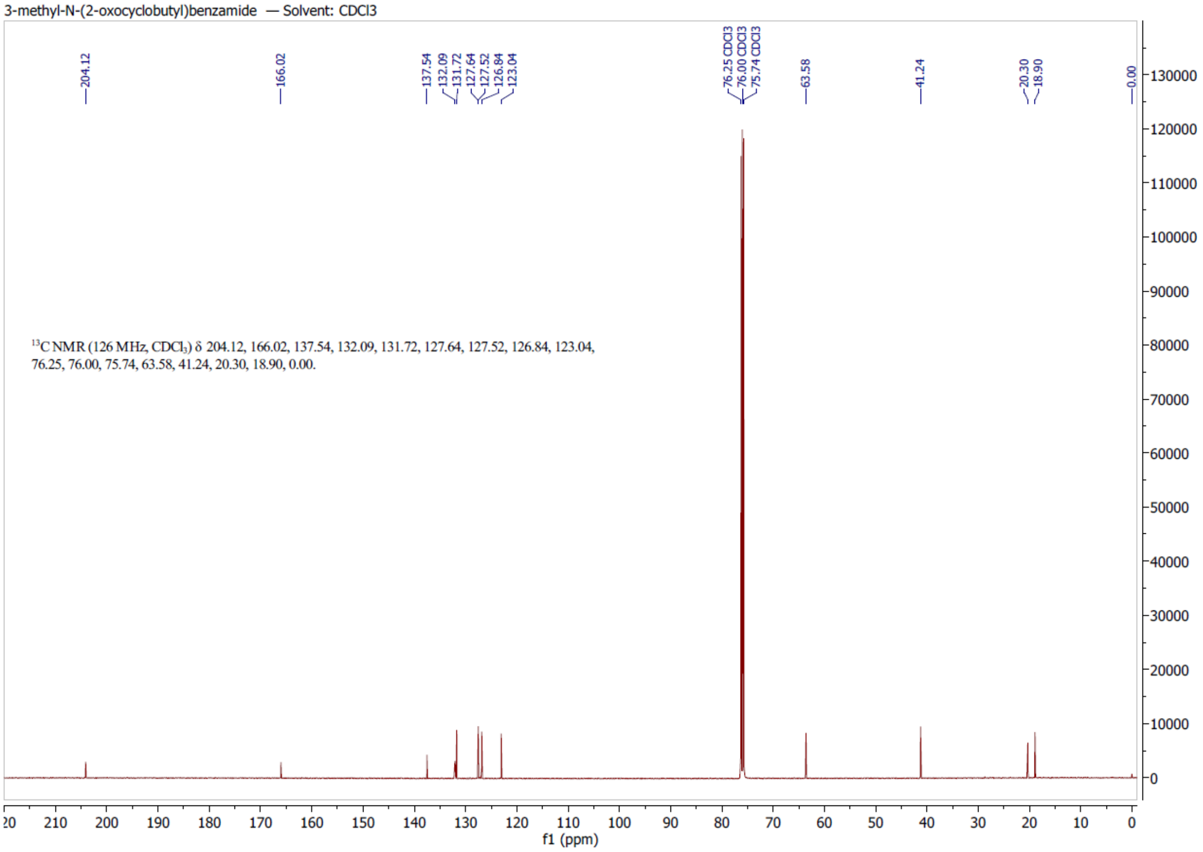

Figure S4. ^{13}C NMR (126 MHz, $CDCl_3$) of 3-methyl-*N*-(2-oxocyclobutyl)benzamide (**3b**).

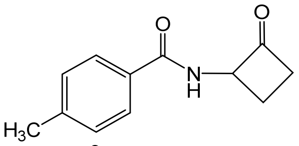

3c

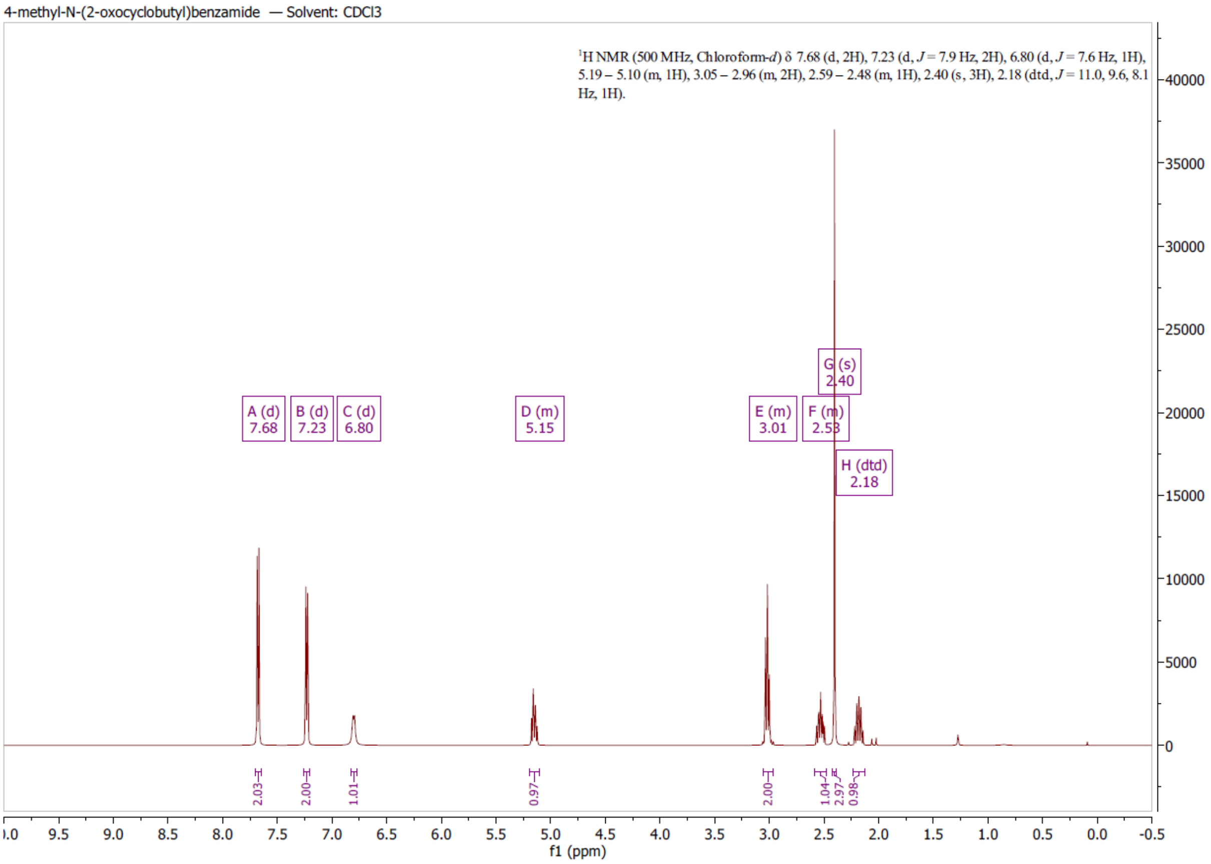

Figure S5. ^{1}H NMR (500 MHz $CDCl_3$) of 4-methyl-*N*-(2-oxocyclobutyl)benzamide (**3c**).

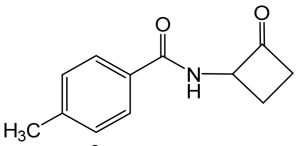

3c

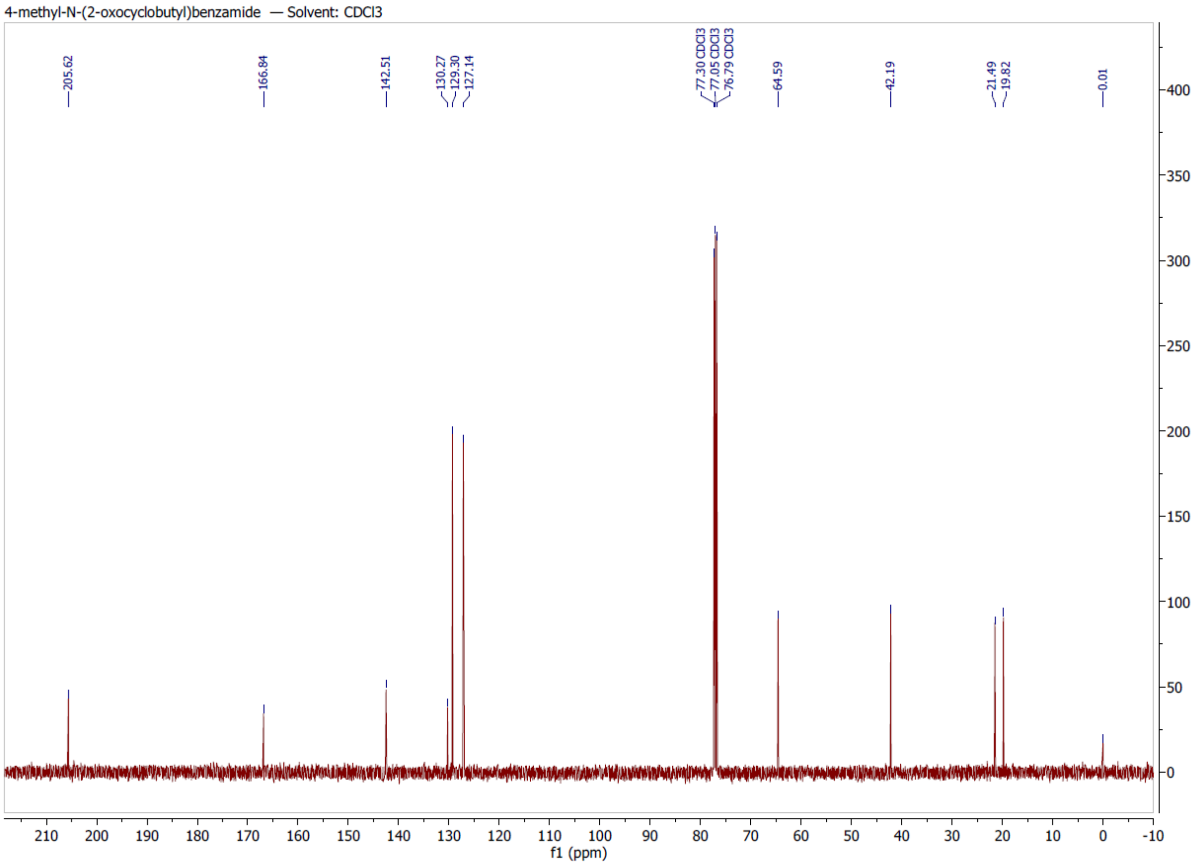

Figure S6. ^{13}C NMR (126 MHz, $CDCl_3$) of 4-methyl-*N*-(2-oxocyclobutyl)benzamide (**3c**).

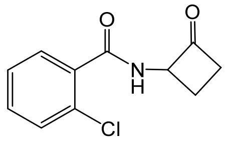

3d

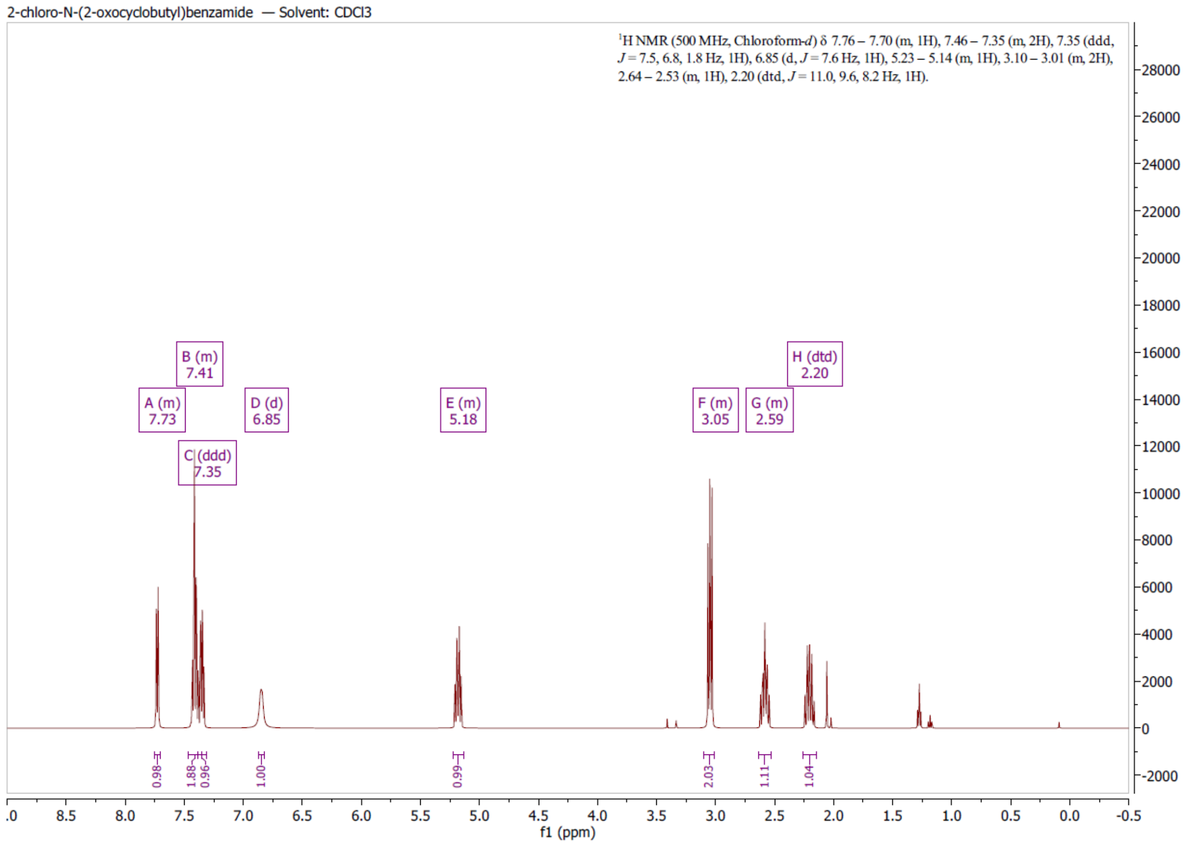

Figure S7. ^{1}H NMR (500 MHz $CDCl_3$) of 2-chloro-*N*-(2-oxocyclobutyl)benzamide **(3d).**

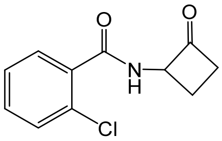

3d

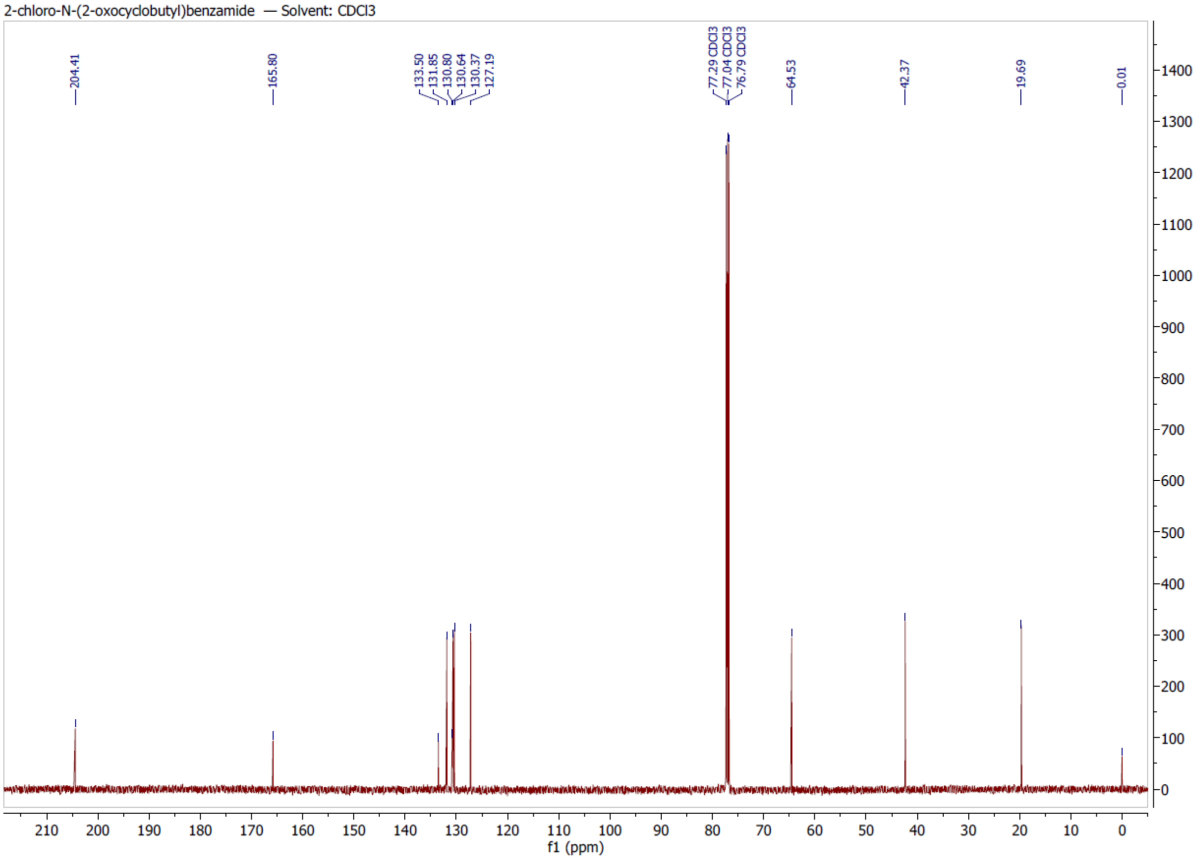

Figure S8. ^{13}C NMR (126 MHz, $CDCl_3$) of 2-chloro-*N*-(2-oxocyclobutyl)benzamide (**3d**).

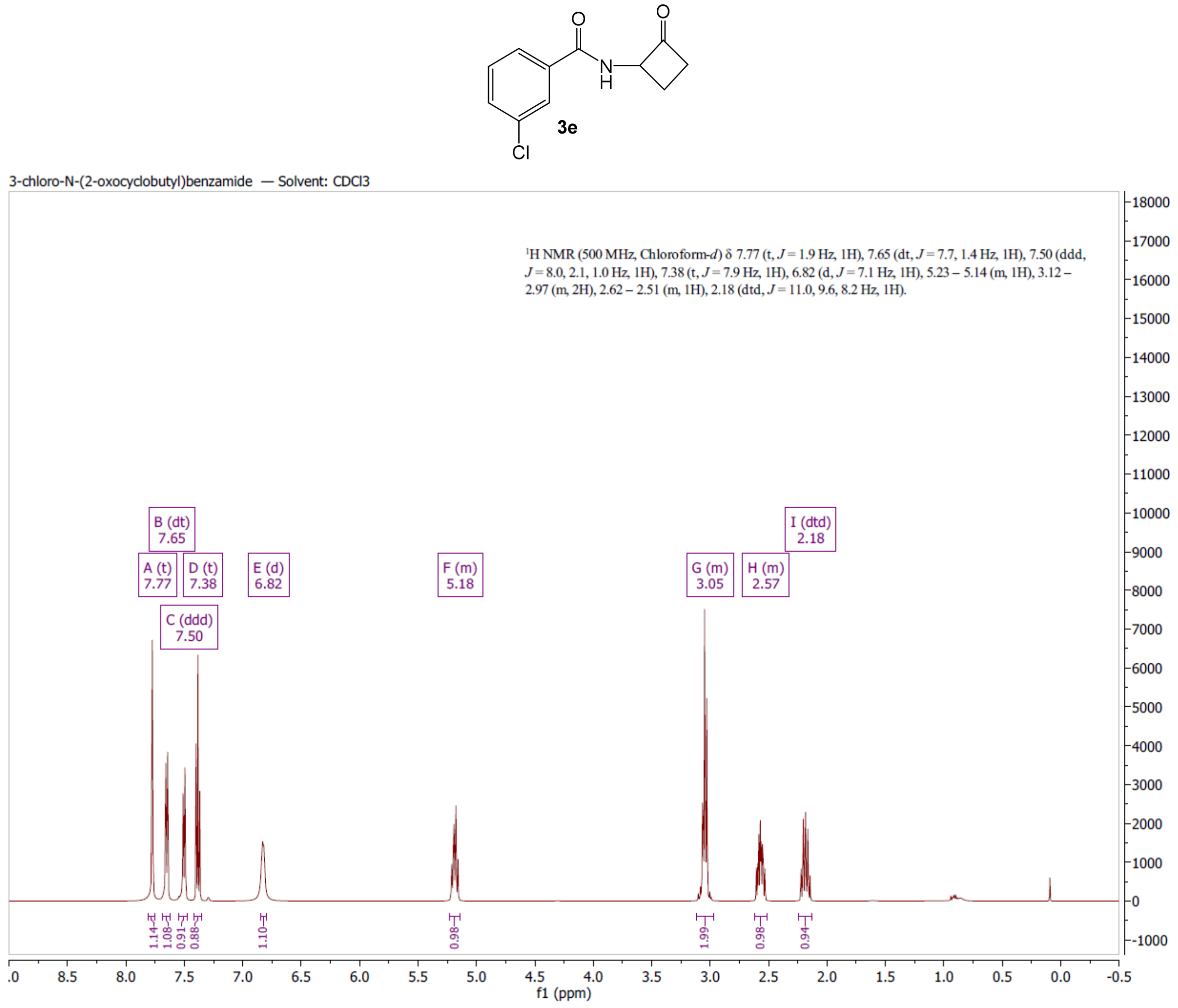

Figure S9. ^{1}H NMR (500 MHz $CDCl_3$) of 3-chloro-*N*-(2-oxocyclobutyl)benzamide (**3e**).

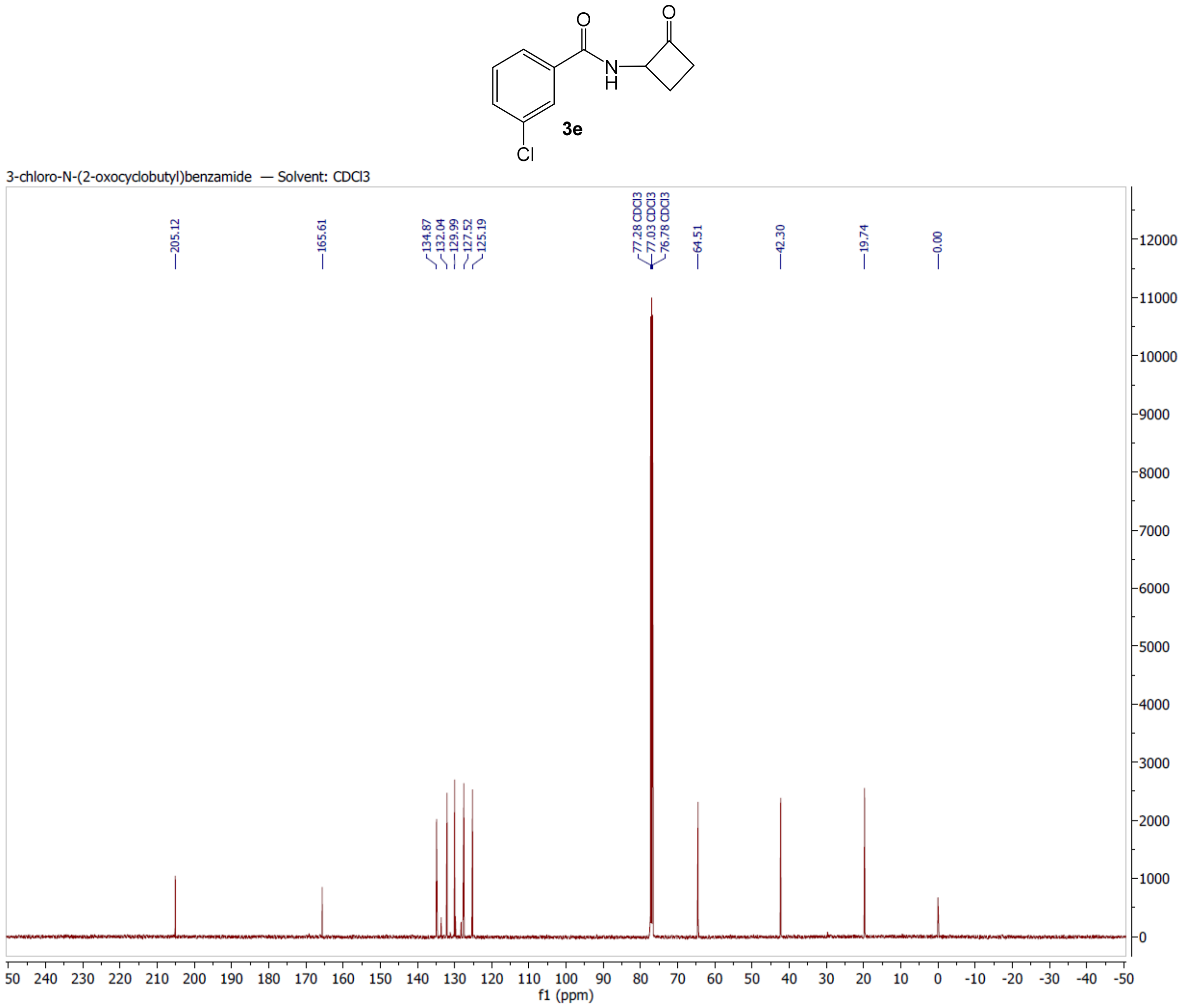

Figure S10. ^{13}C NMR (126 MHz, $CDCl_3$) of 3-chloro-*N*-(2-oxocyclobutyl)benzamide (**3e**).

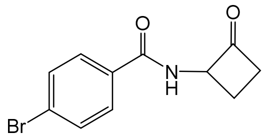

3f

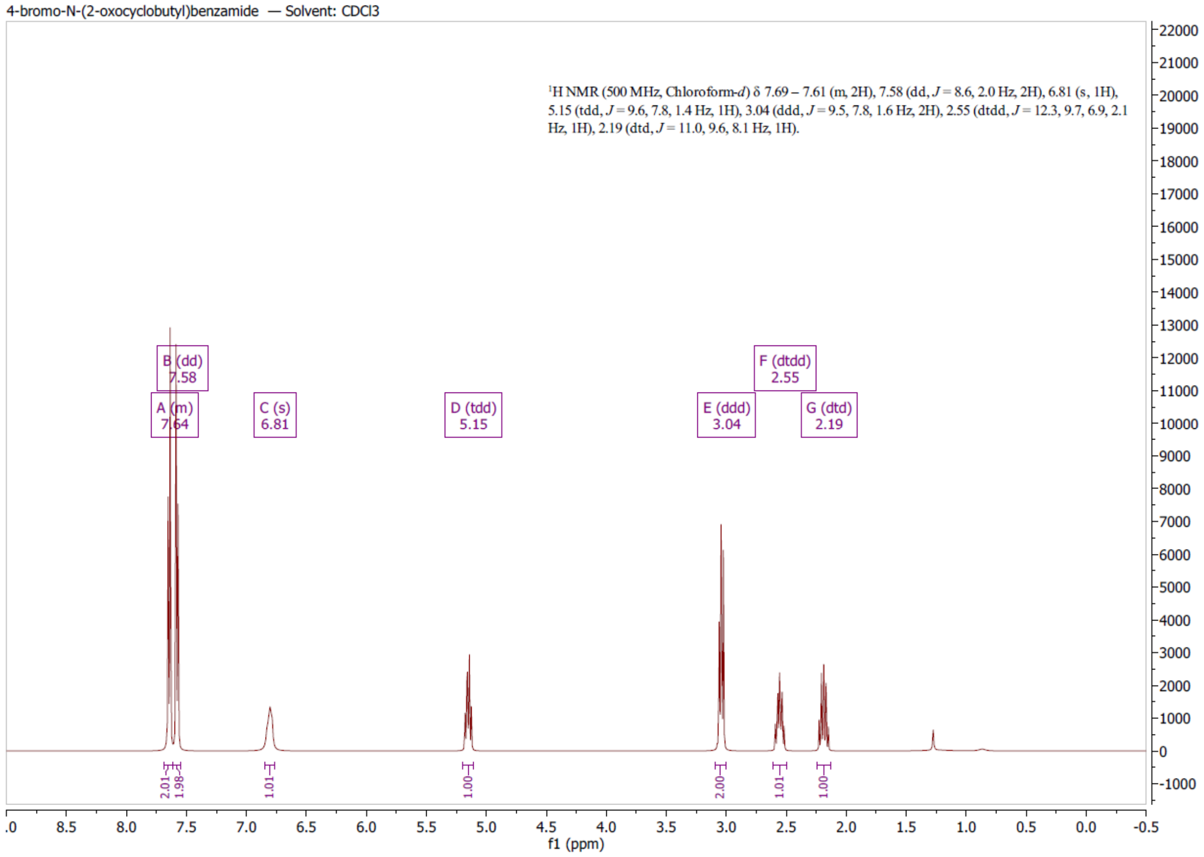

Figure S11. ¹H NMR (500 MHz $CDCl_3$) of 4-bromo-*N*-(2-oxocyclobutyl)benzamide (**3f**).

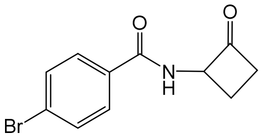

3f

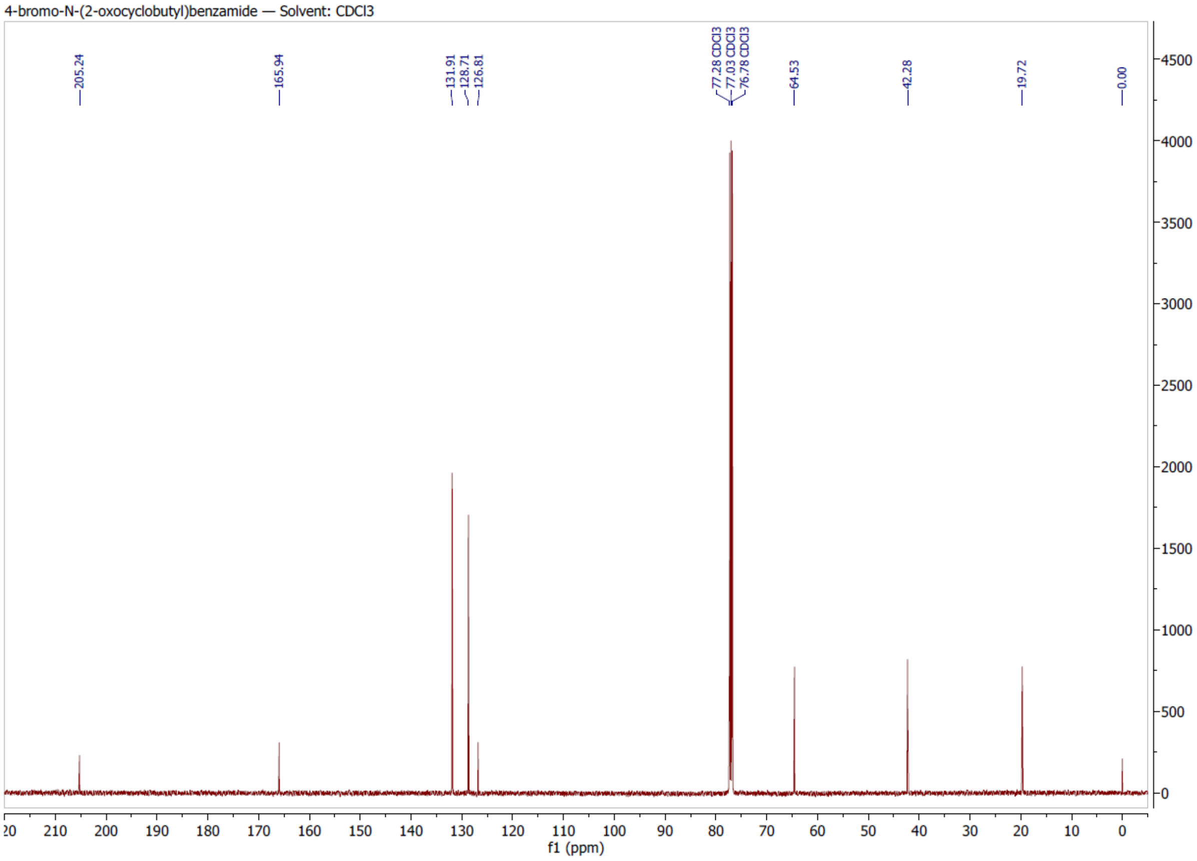

Figure S12. ^{13}C NMR (126 MHz, $CDCl_3$) of 4-bromo-*N*-(2-oxocyclobutyl)benzamide (**3f**).

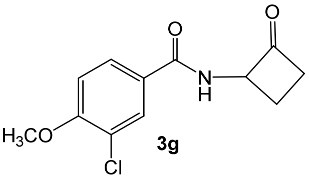

3g

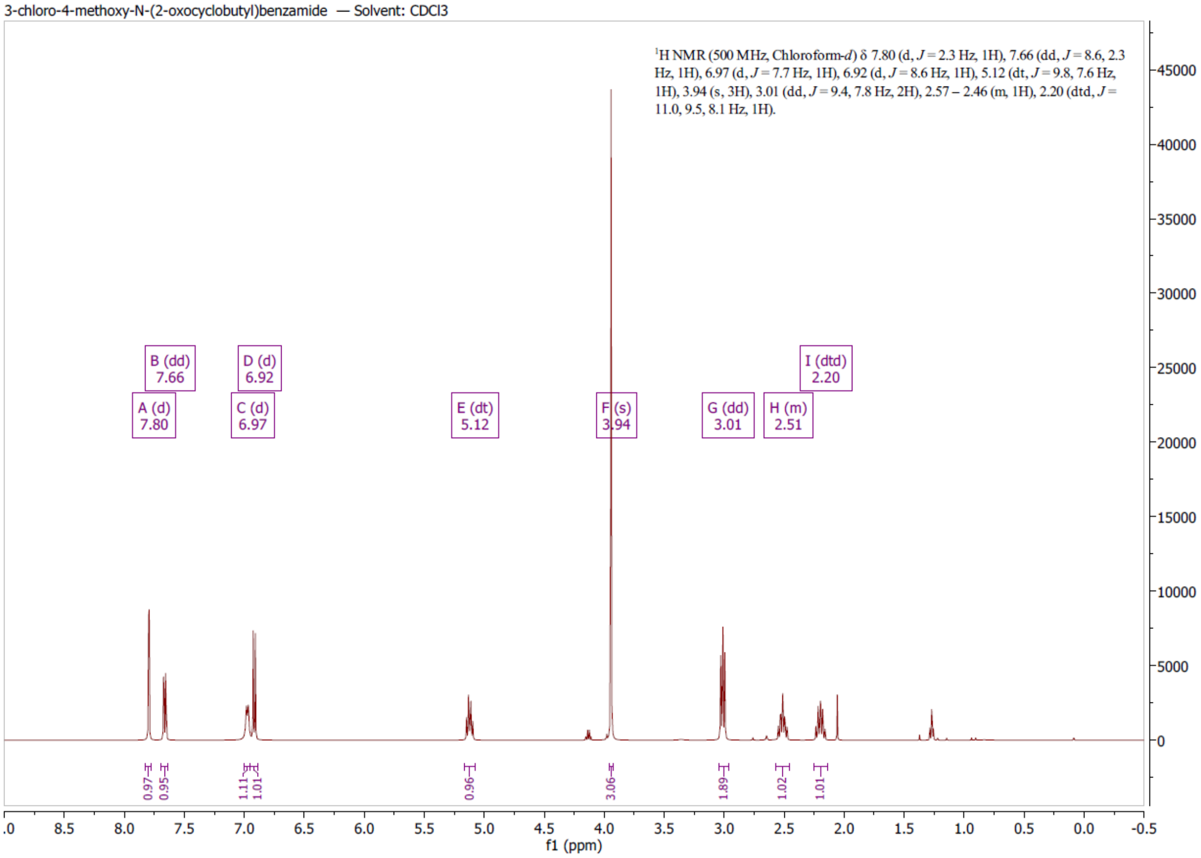

Figure S13. ^{1}H NMR (500 MHz $CDCl_3$) of 3-chloro-4-methoxy-*N*-(2-oxocyclobutyl)benzamide (**3g**).

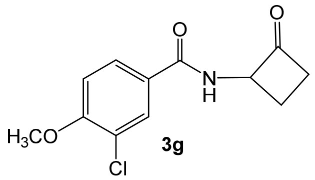

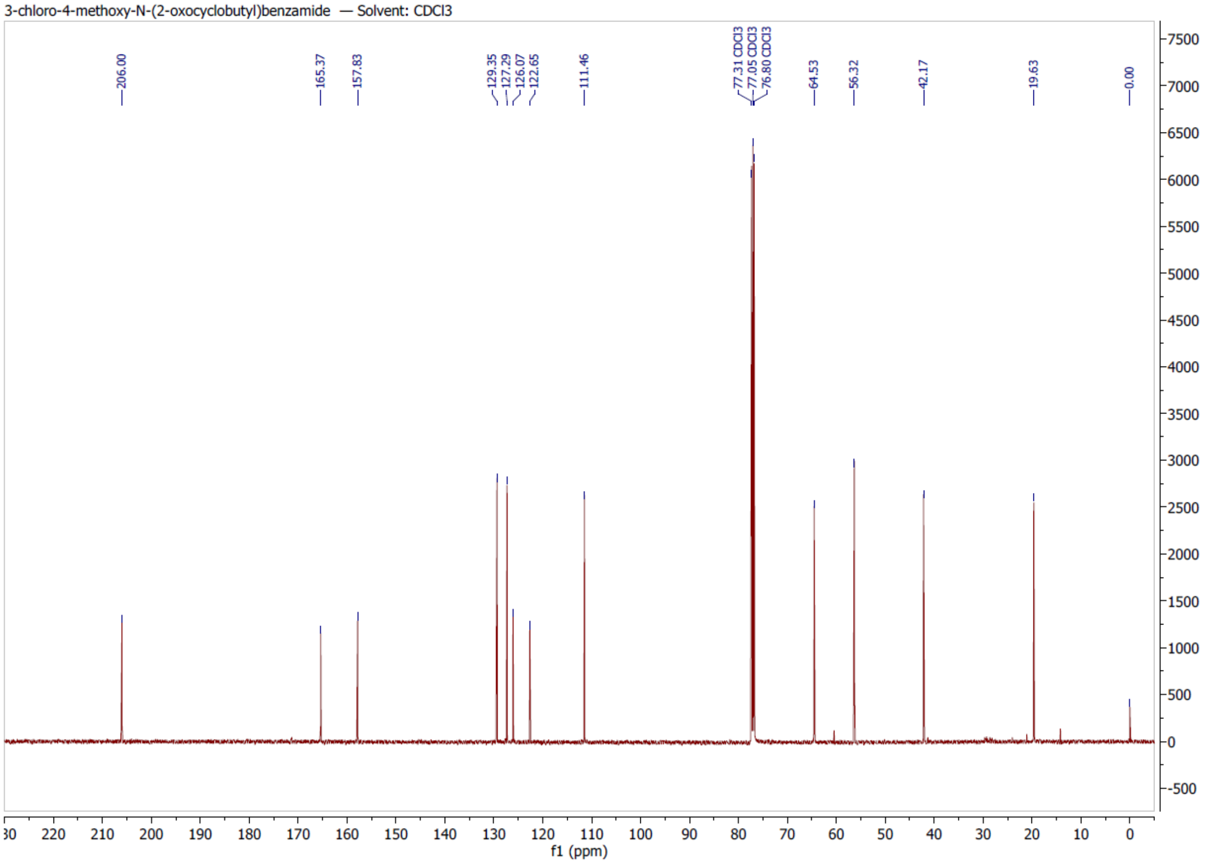

Figure S14. ^{13}C NMR (126 MHz, $CDCl_3$) of 3-chloro-4-methoxy-*N*-(2-oxocyclobutyl)benzamide (**3g**).

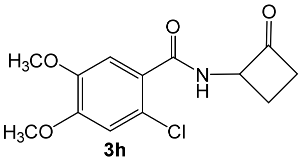

3h

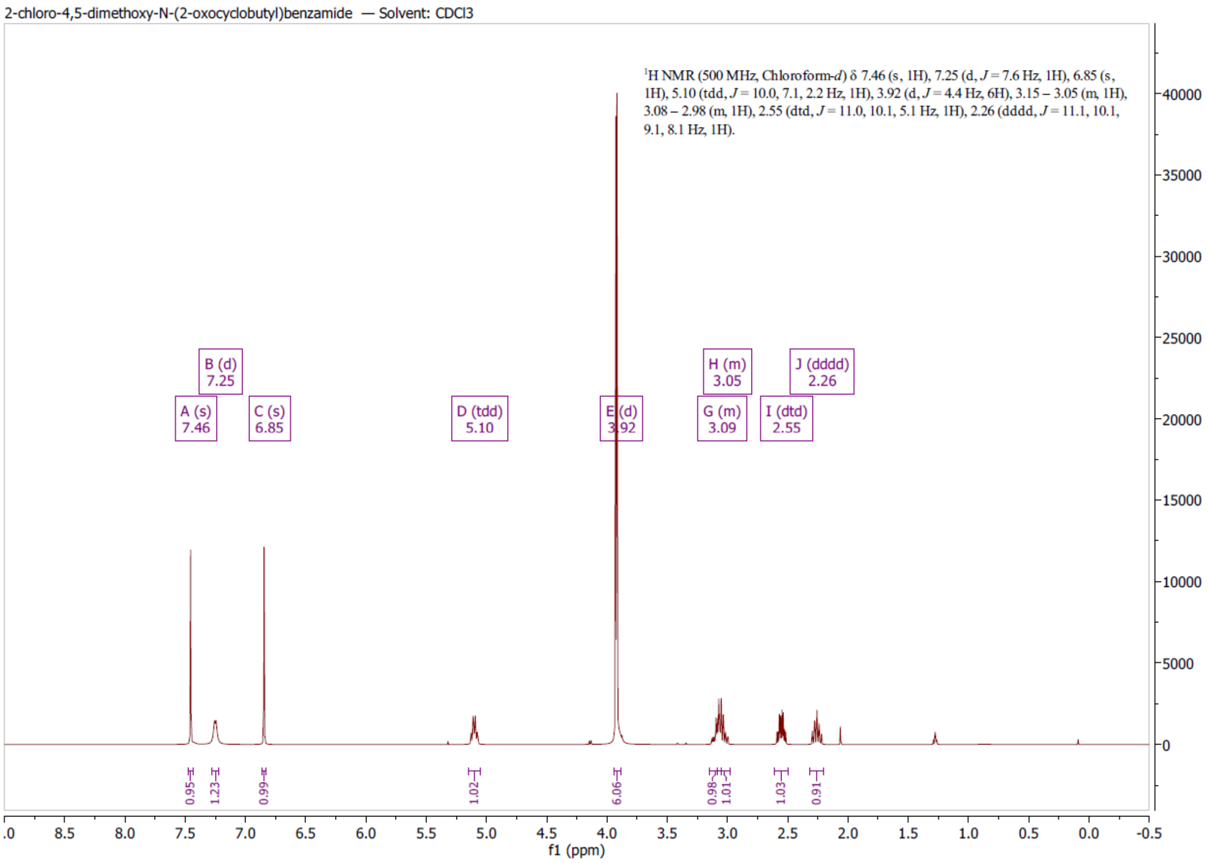

Figure S15. ^{1}H NMR (500 MHz $CDCl_3$) of 2-chloro-4,5-dimethoxy-*N*-(2-oxocyclobutyl)benzamide (**3h**).

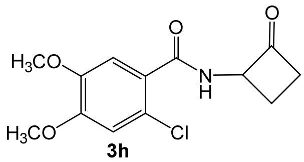

3h

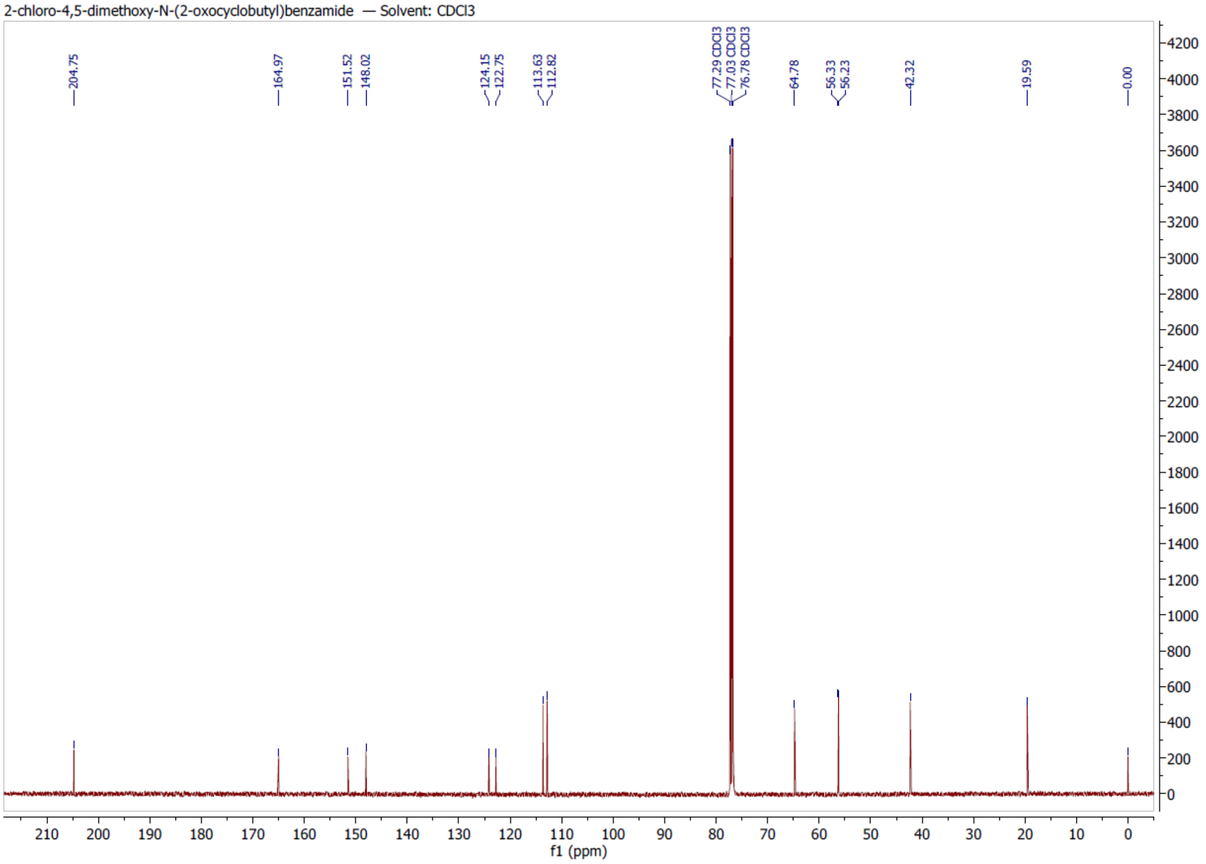

Figure S16. ^{13}C NMR (126 MHz, $CDCl_3$) of 2-chloro-4,5-dimethoxy-*N*-(2-oxocyclobutyl)benzamide (**3h**).

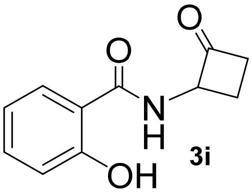

3i

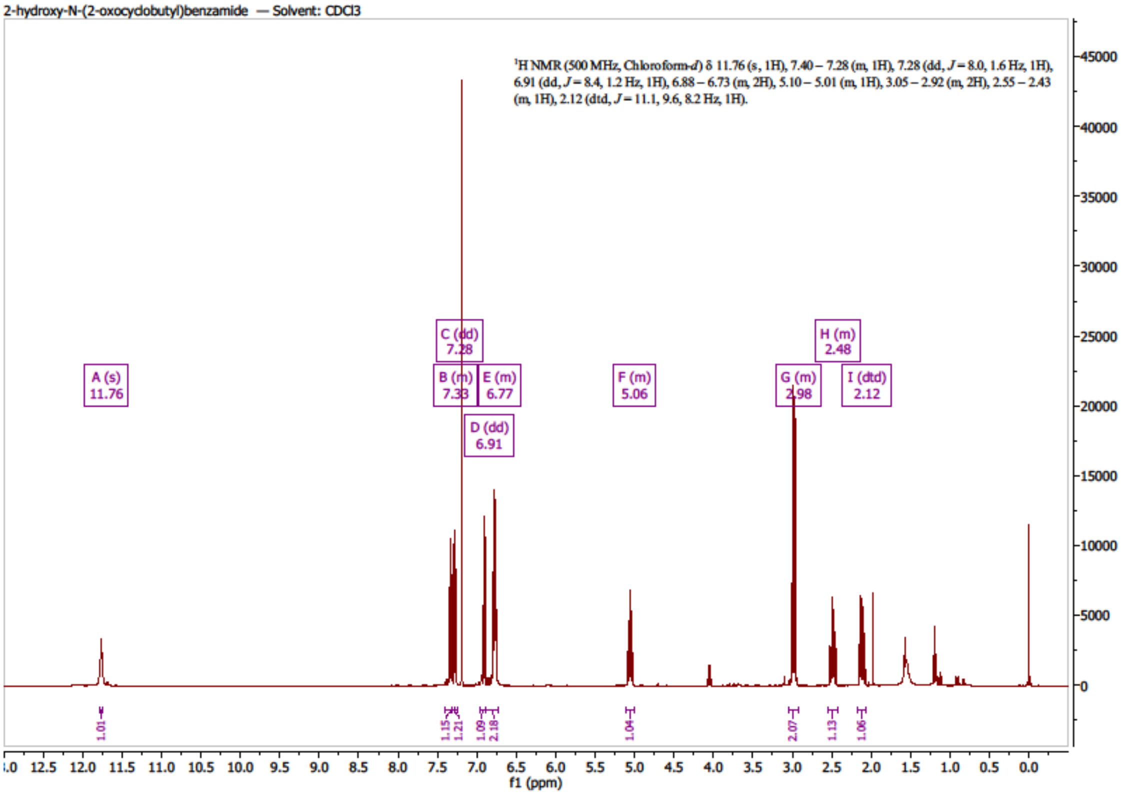

Figure S17. ^{1}H NMR (500 MHz $CDCl_3$) of 2-hydroxy-*N*-(2-oxocyclobutyl)benzamide (**3i**)

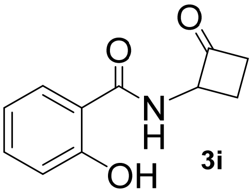

3i

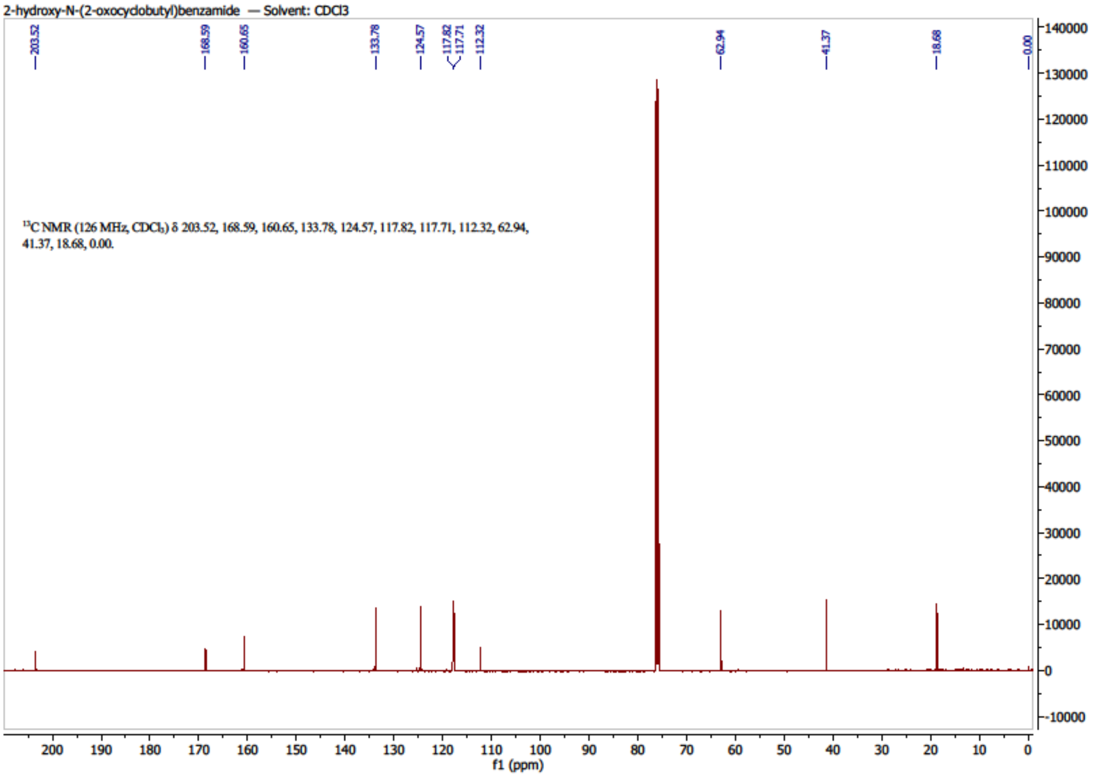

Figure S18. ^{13}C NMR (126 MHz, $CDCl_3$) of 2-hydroxy-*N*-(2-oxocyclobutyl)benzamide (**3i**)

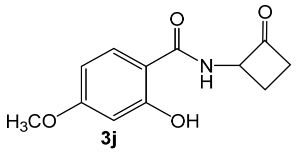

3j

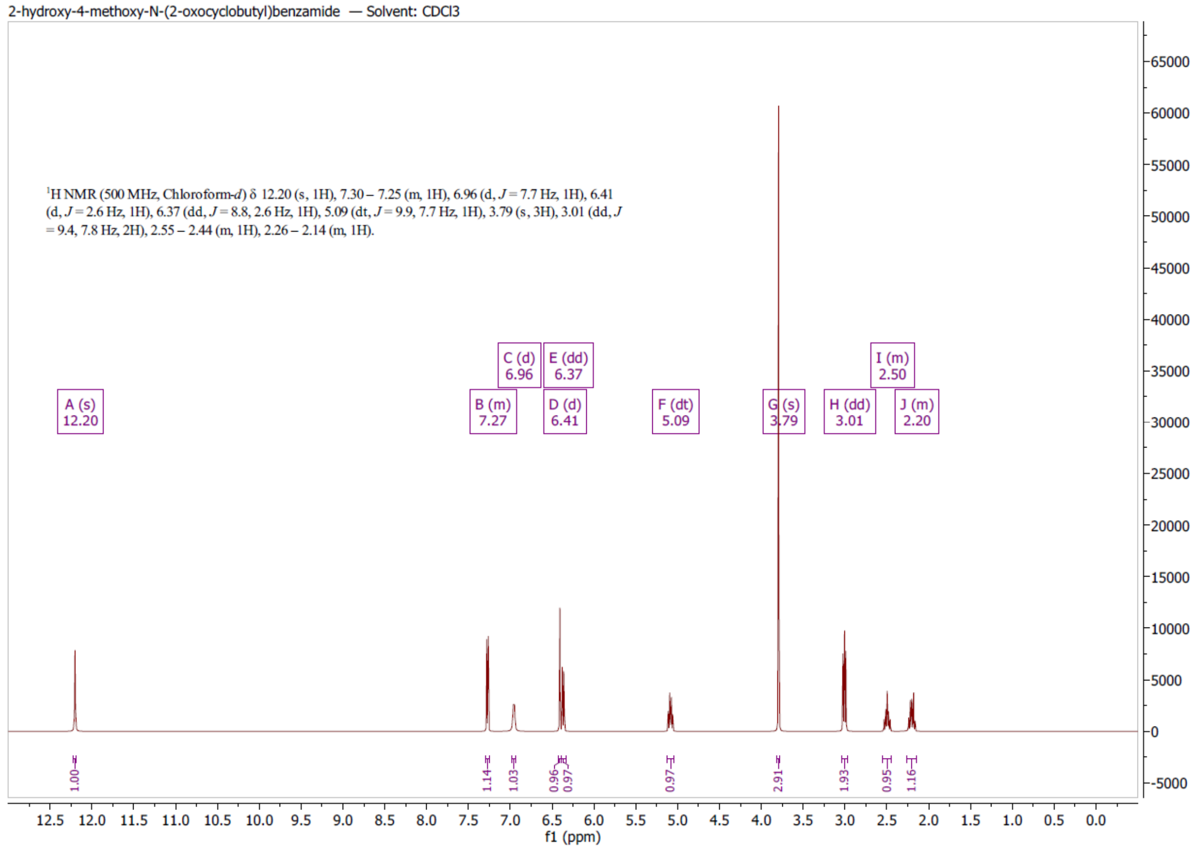

Figure S19. ^{1}H NMR (500 MHz $CDCl_3$) of 2-hydroxy-4-methoxy-*N*-(2-oxocyclobutyl)benzamide (**3j**).

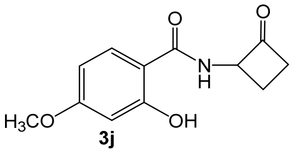

3j

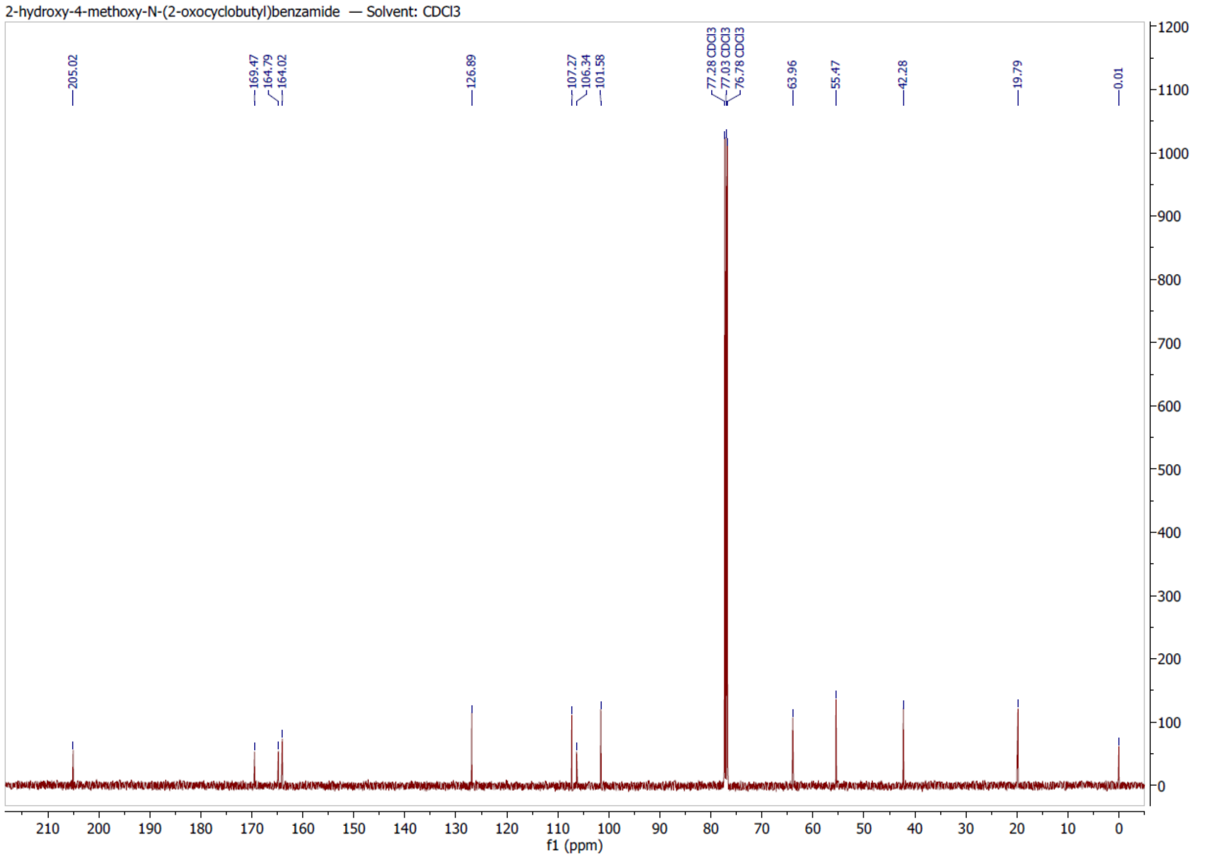

Figure S20. ^{13}C NMR (126 MHz, $CDCl_3$) of 2-hydroxy-4-methoxy-*N*-(2-oxocyclobutyl)benzamide (**3j**).

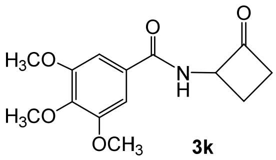

3k

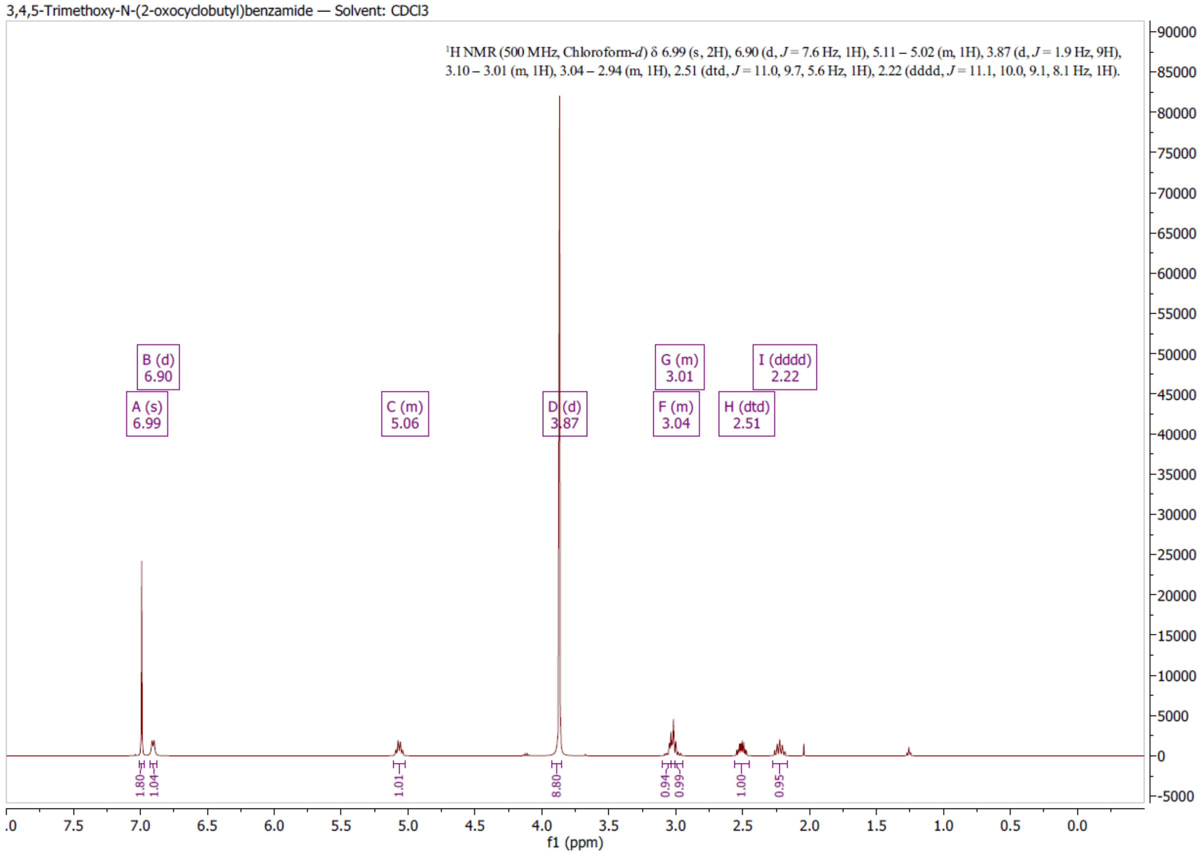

Figure S21. ^{1}H NMR (500 MHz $CDCl_3$) of 3,4,5-trimethoxy-*N*-(2-oxocyclobutyl)benzamide (**3k**).

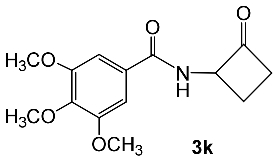

3k

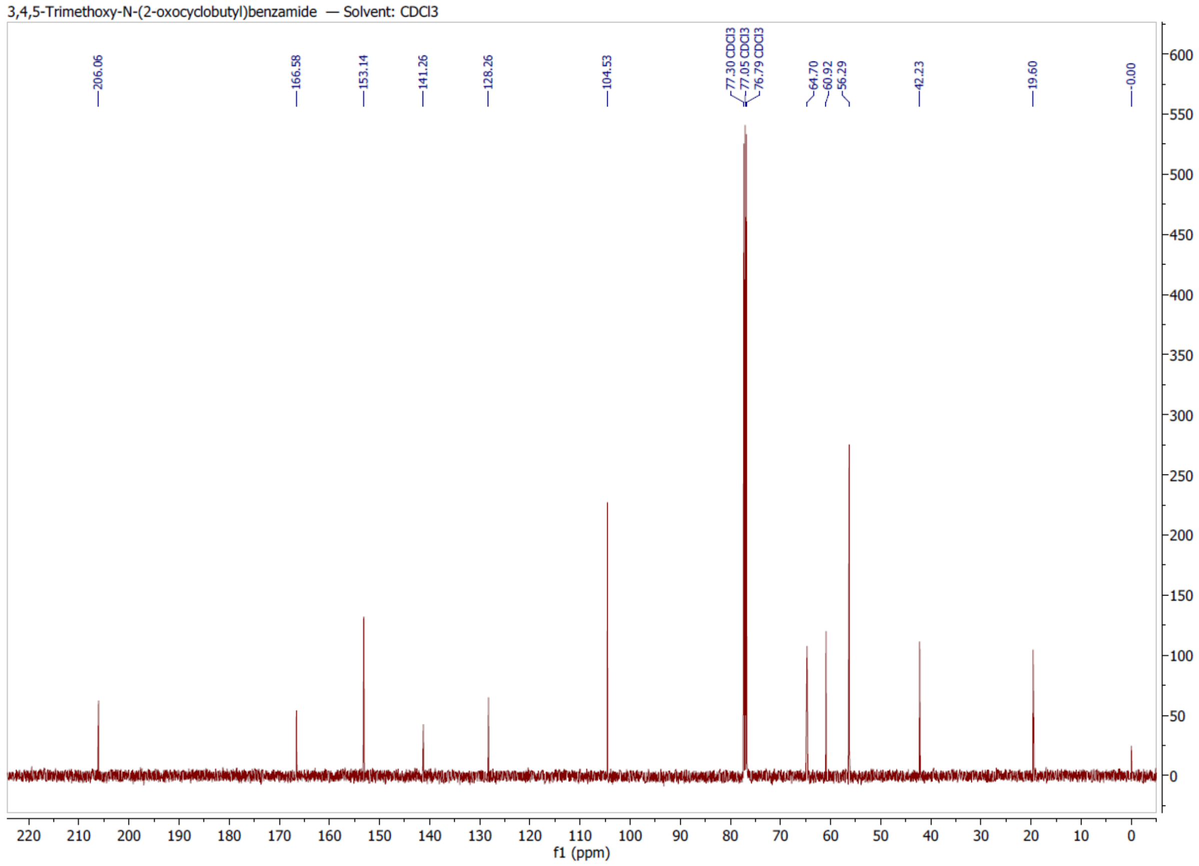

Figure S22. ^{13}C NMR (126 MHz, $CDCl_3$) of 3,4,5-trimethoxy-*N*-(2-oxocyclobutyl)benzamide (**3k**).

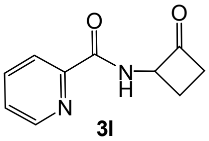

3l

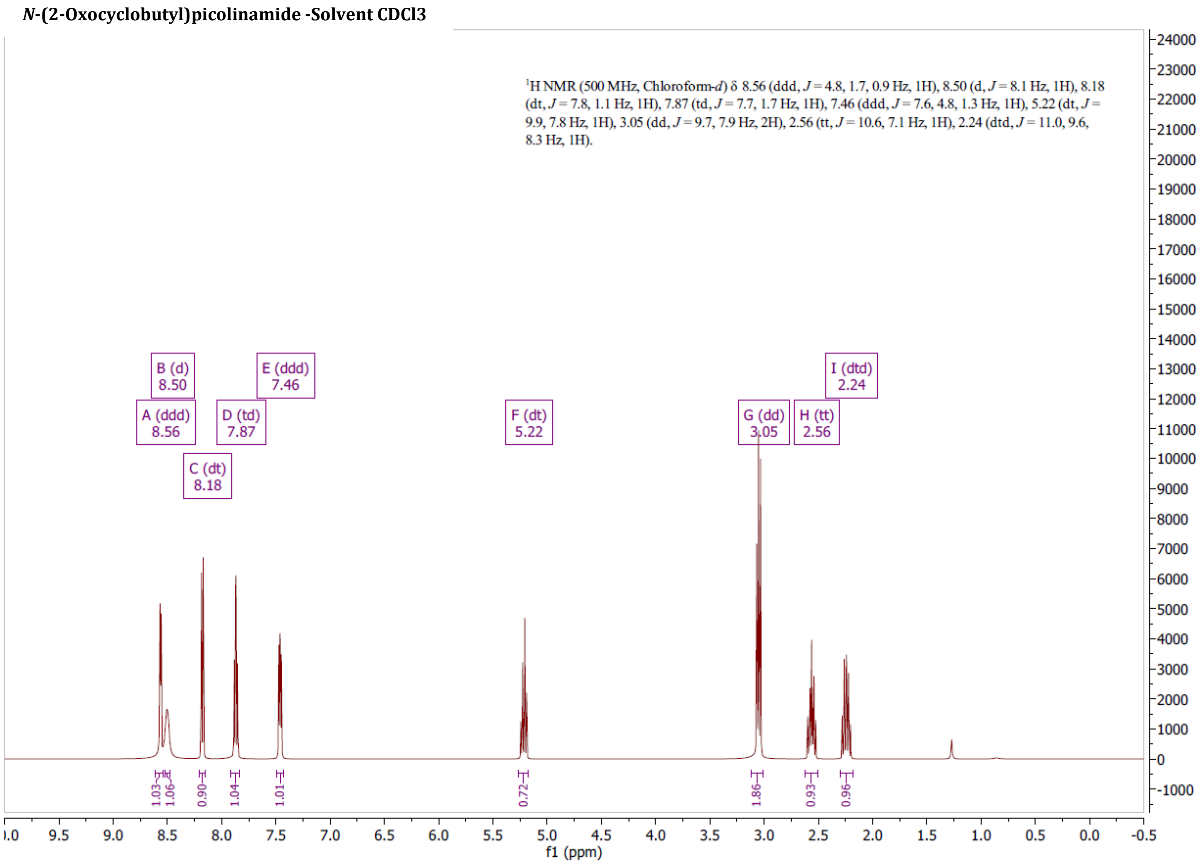

Figure S23. ^{1}H NMR (500 MHz $CDCl_3$) of *N*-(2-oxocyclobutyl)picolinamide (**3l**).

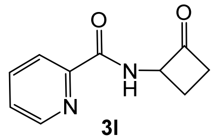

3l

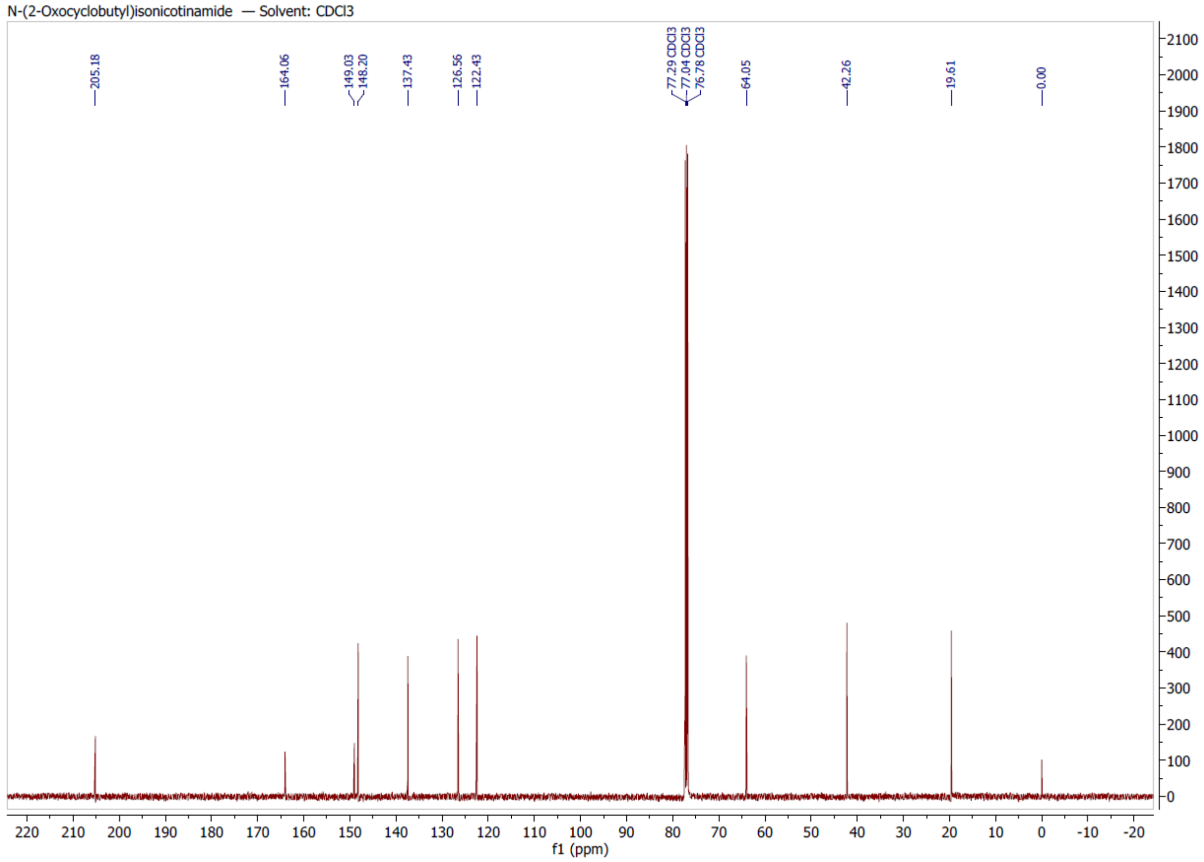

Figure S24. ^{13}C NMR (126 MHz, $CDCl_3$) of *N*-(2-oxocyclobutyl)picolinamide (**3l**).

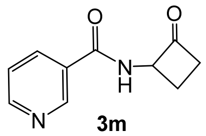

3m

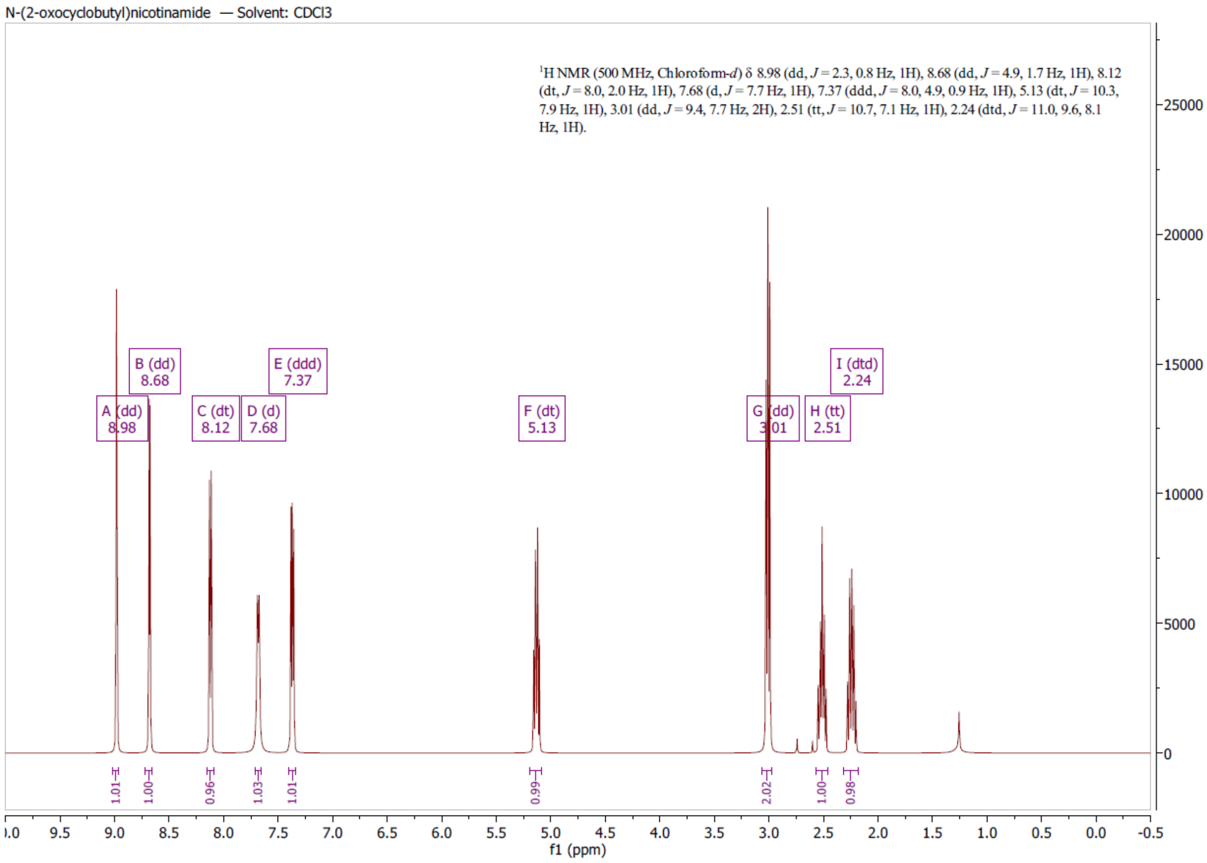

Figure S25. ^{1}H NMR (500 MHz $CDCl_3$) of *N*-(2-oxocyclobutyl)nicotinamide (**3m**).

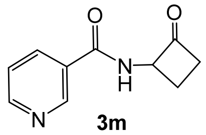

3m

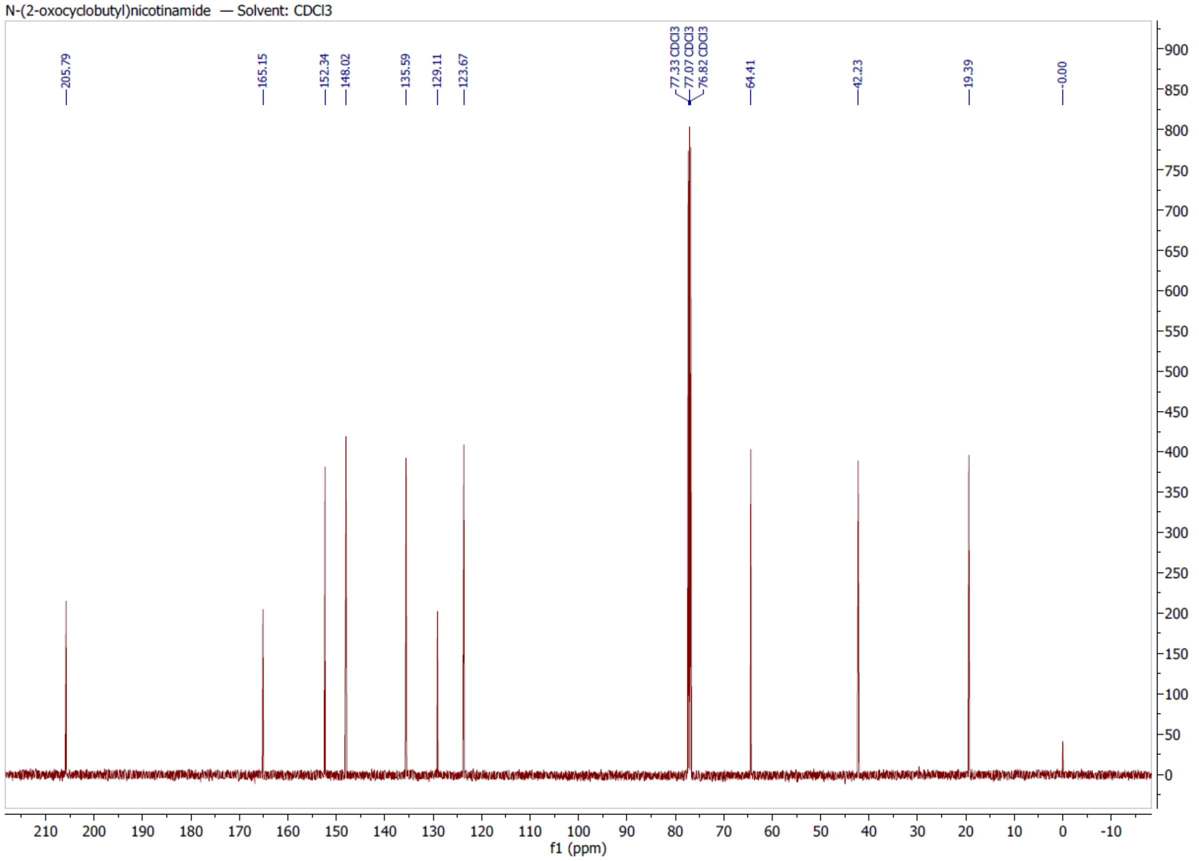

Figure S26. ^{13}C NMR (126 MHz, $CDCl_3$) of *N*-(2-oxocyclobutyl)nicotinamide (**3m**).

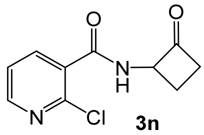

3n

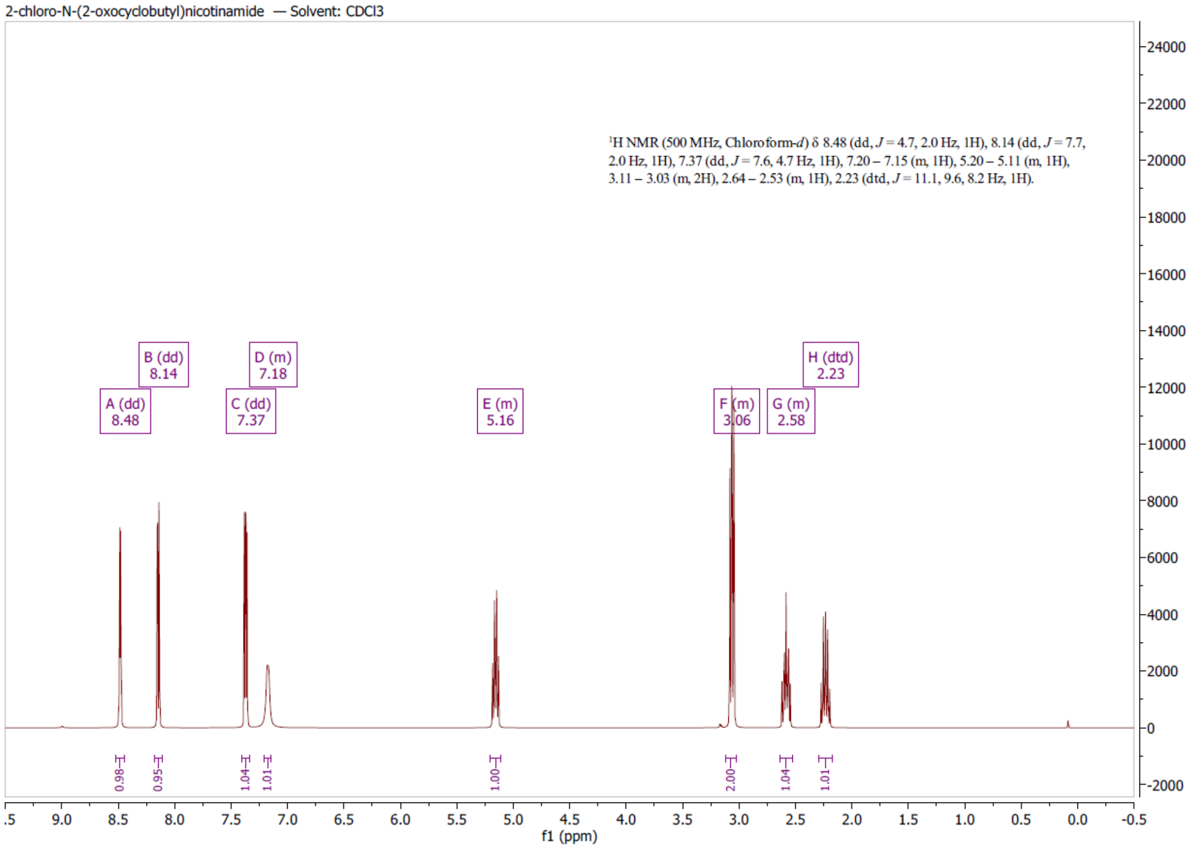

Figure S27. ^{1}H NMR (500 MHz $CDCl_3$) of 2-chloro-*N*-(2-oxocyclobutyl)nicotinamide (**3n**).

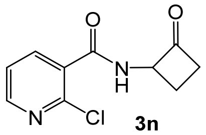

3n

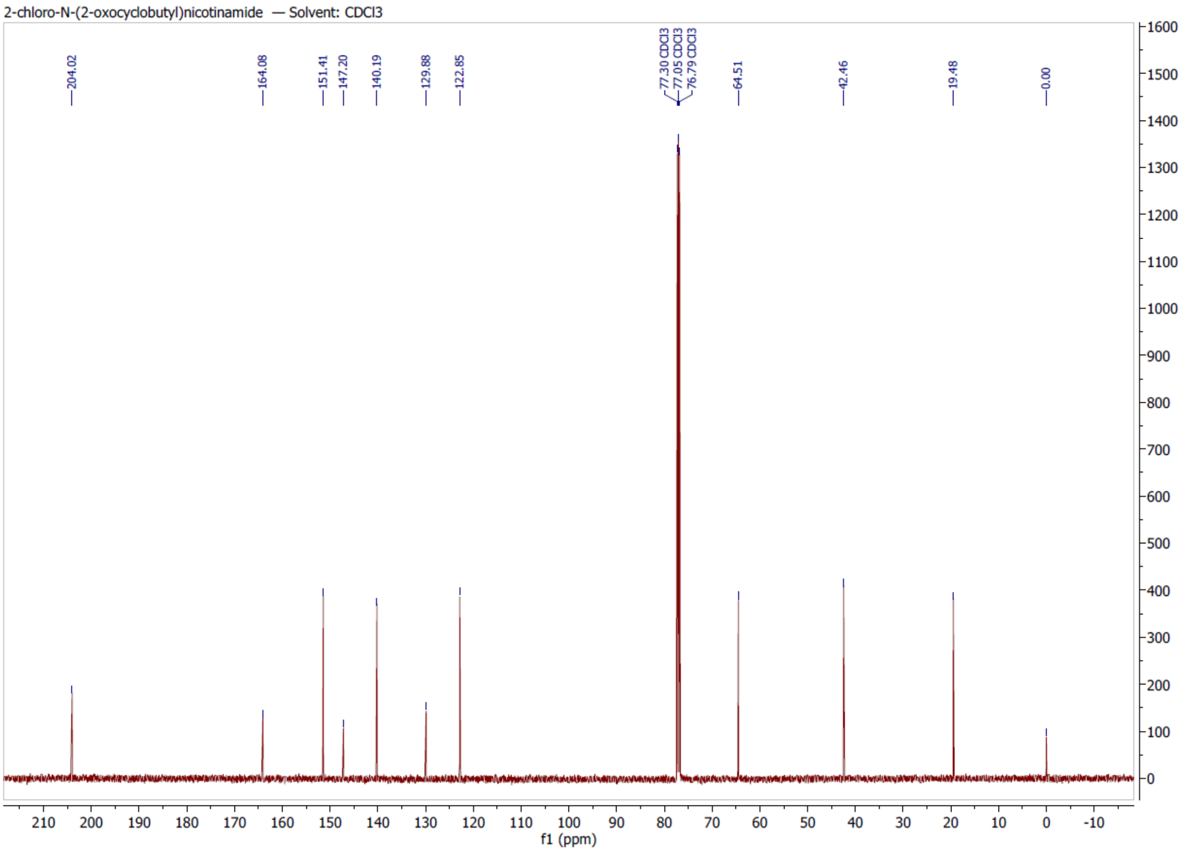

Figure 28. ^{13}C NMR (126 MHz, $CDCl_3$) of 2-chloro-*N*-(2-oxocyclobutyl)nicotinamide (**3n**).

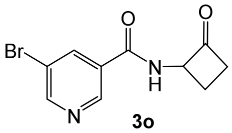

3o

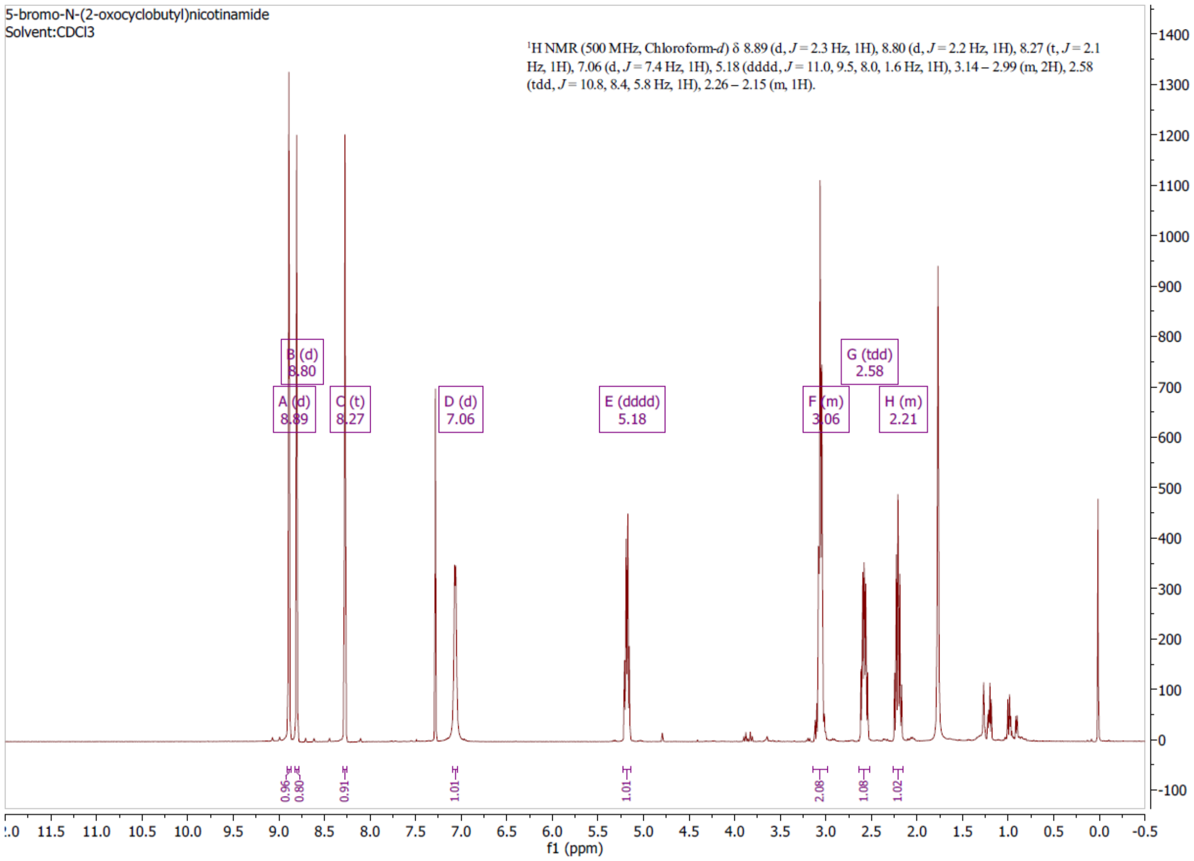

Figure S29. ¹H NMR (500 MHz $CDCl_3$) of 5-bromo-*N*-(2-oxocyclobutyl)nicotinamide (**3o**).

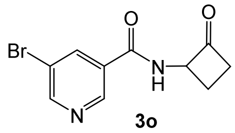

3o

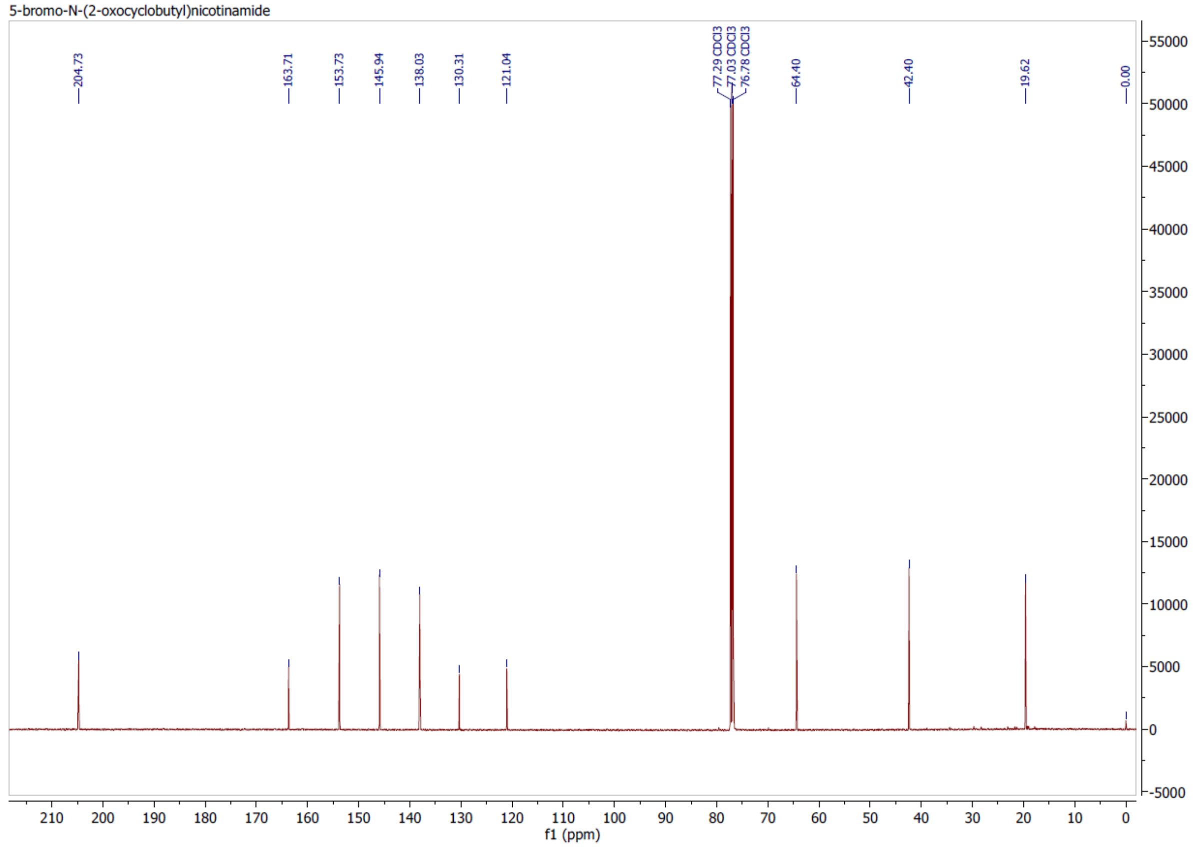

Figure S30. ^{13}C NMR (126 MHz, $CDCl_3$) of 5-bromo-*N*-(2-oxocyclobutyl)nicotinamide (**3o**).

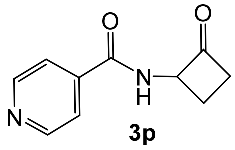

3p

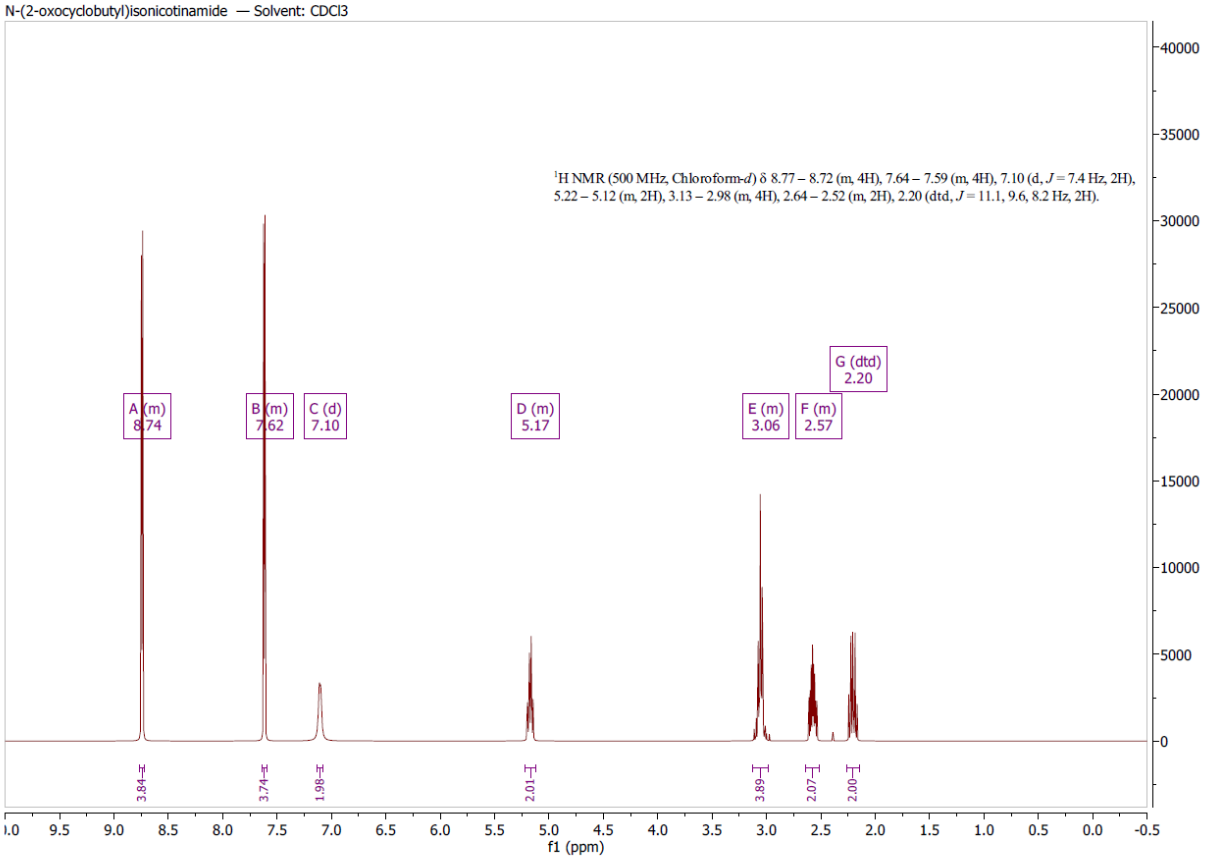

Figure S31. ^{1}H NMR (500 MHz $CDCl_3$) of *N*-(2-oxocyclobutyl)isonicotinamide (**3p**).

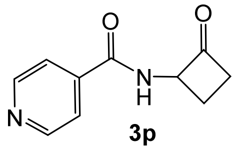

3p

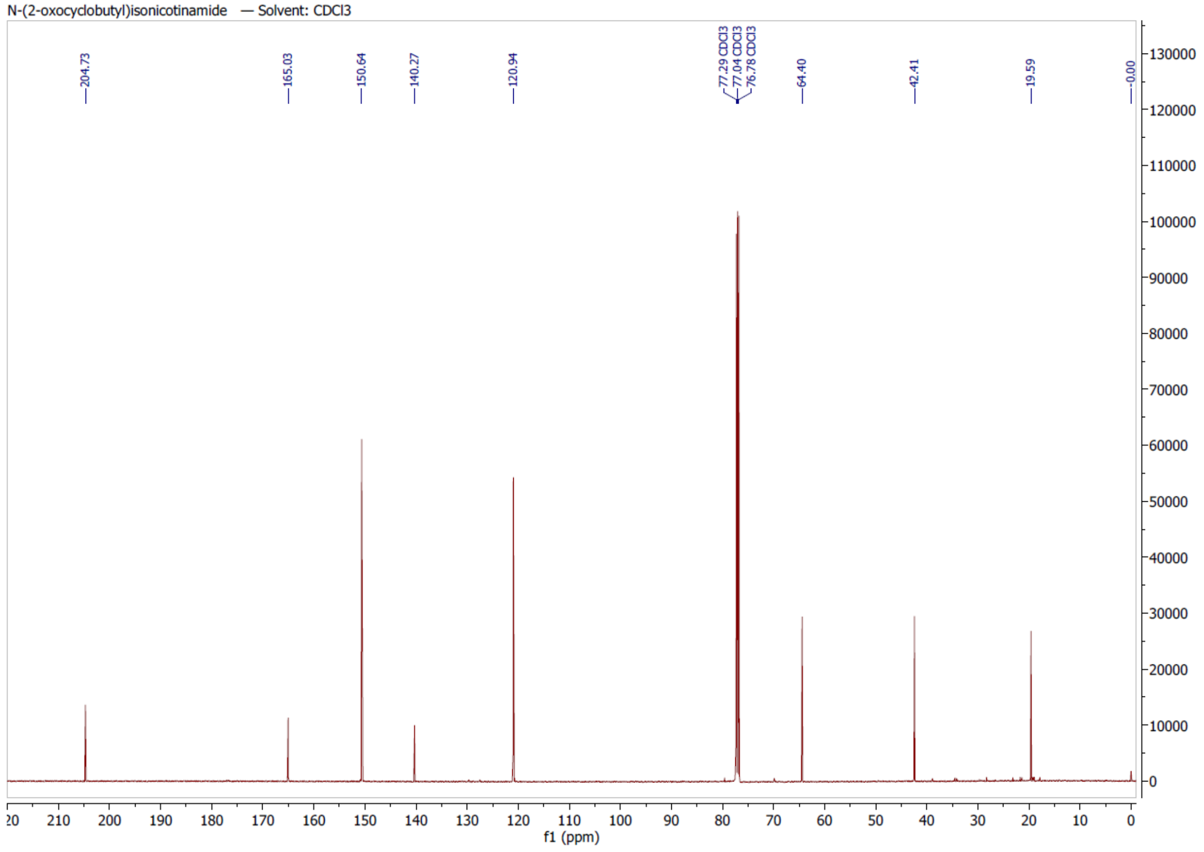

Figure S32. ^{13}C NMR (126 MHz, $CDCl_3$) of *N*-(2-oxocyclobutyl)isonicotinamide (**3p**).

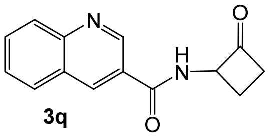

3q

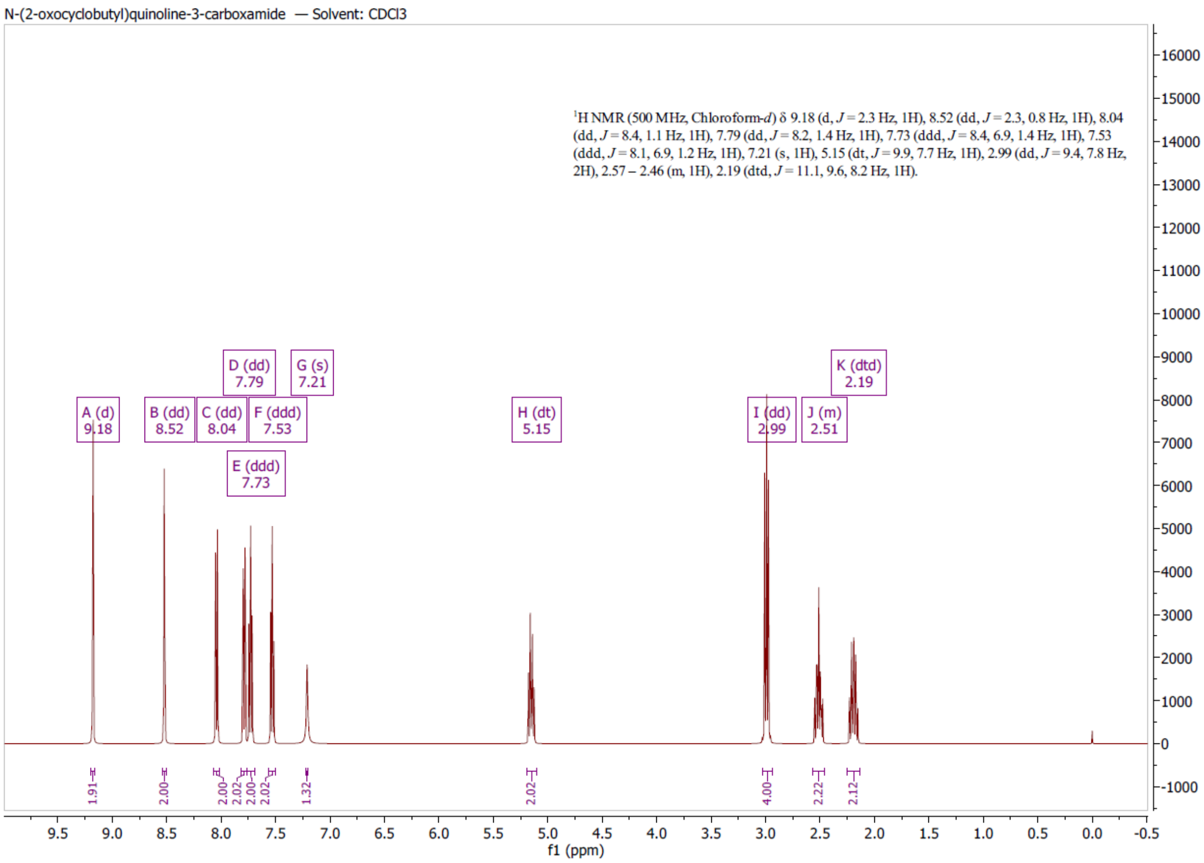

Figure S33. ^{1}H NMR (500 MHz $CDCl_3$) of *N*-(2-oxocyclobutyl)quinoline-3-carboxamide (**3q**).

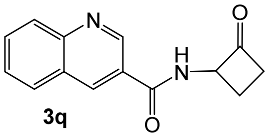

3q

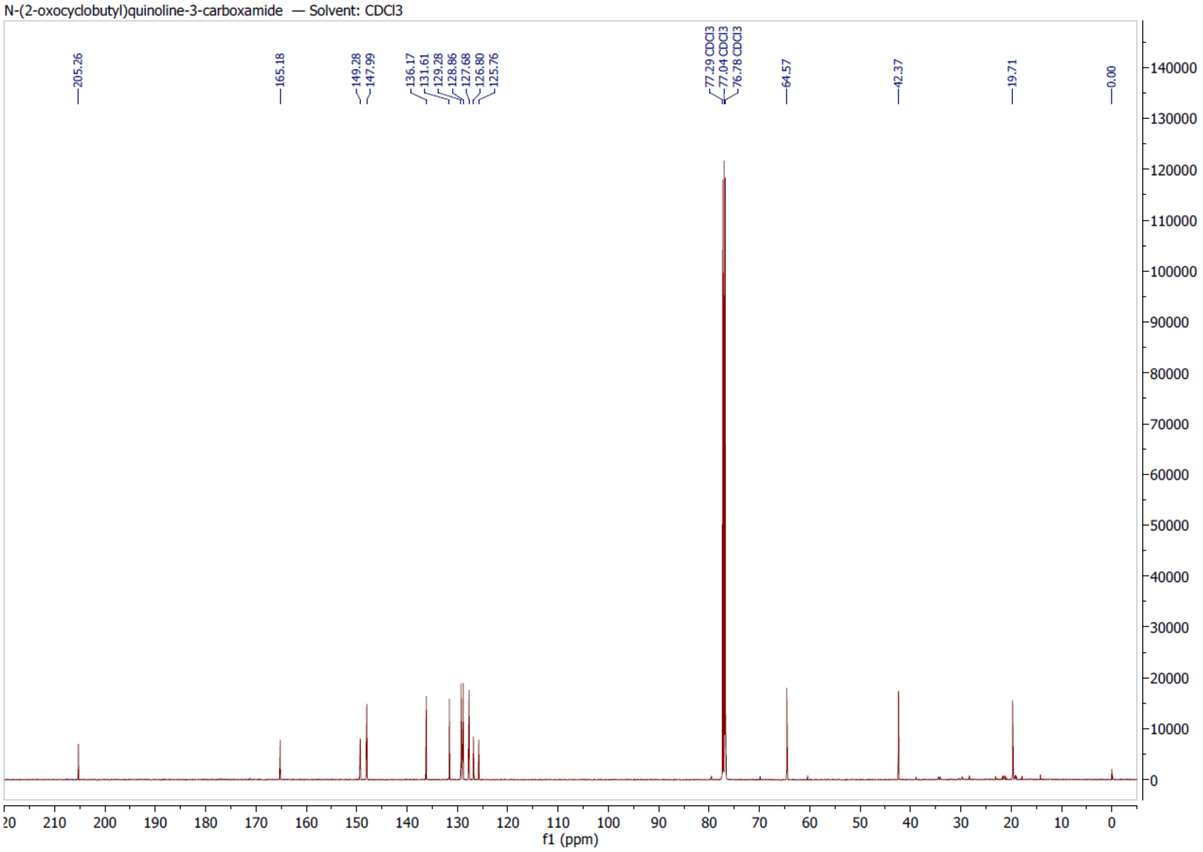

Figure S34. ^{13}C NMR (126 MHz, $CDCl_3$) of *N*-(2-oxocyclobutyl)quinoline-3-carboxamide (**3q**).

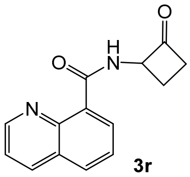

3r

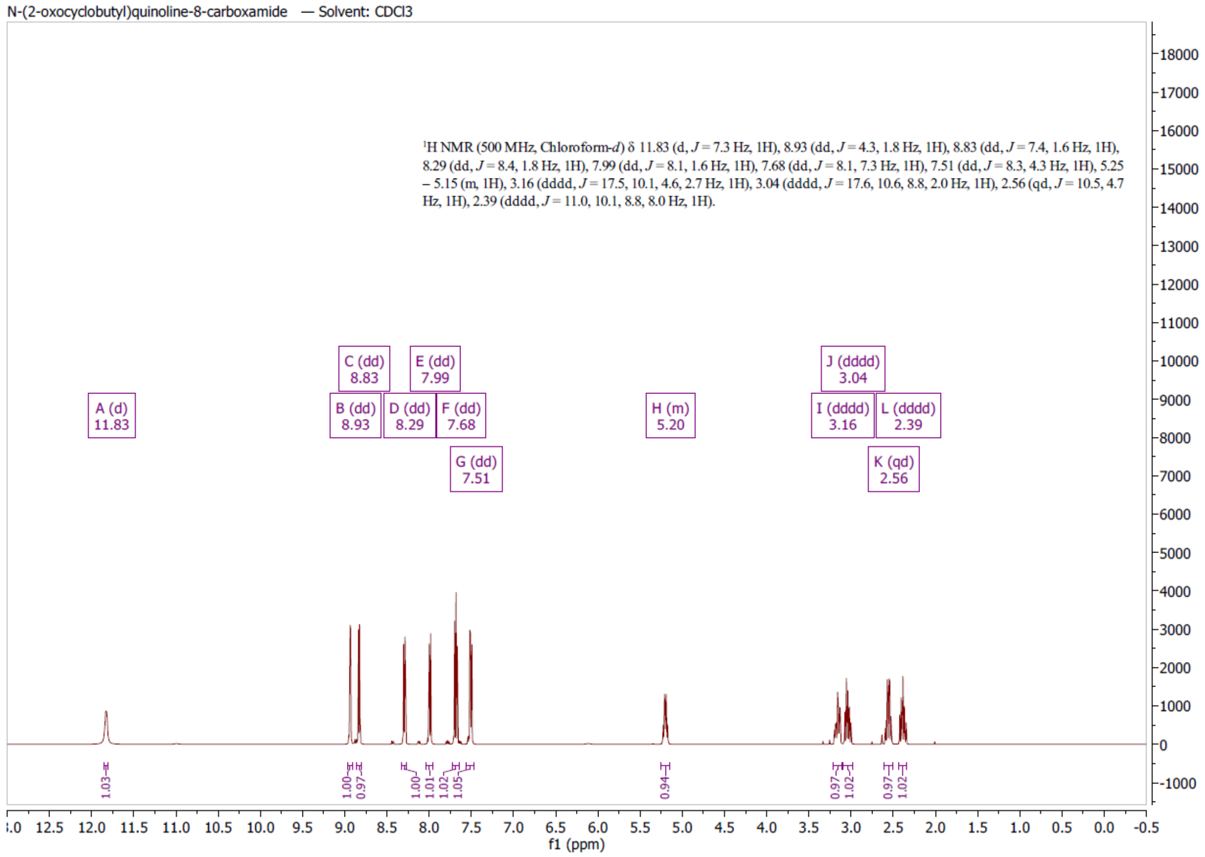

Figure S35. ^{1}H NMR (500 MHz $CDCl_3$) of *N*-(2-oxocyclobutyl)quinoline-8-carboxamide (**3r**).

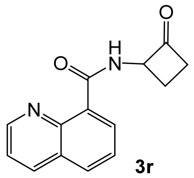

3r

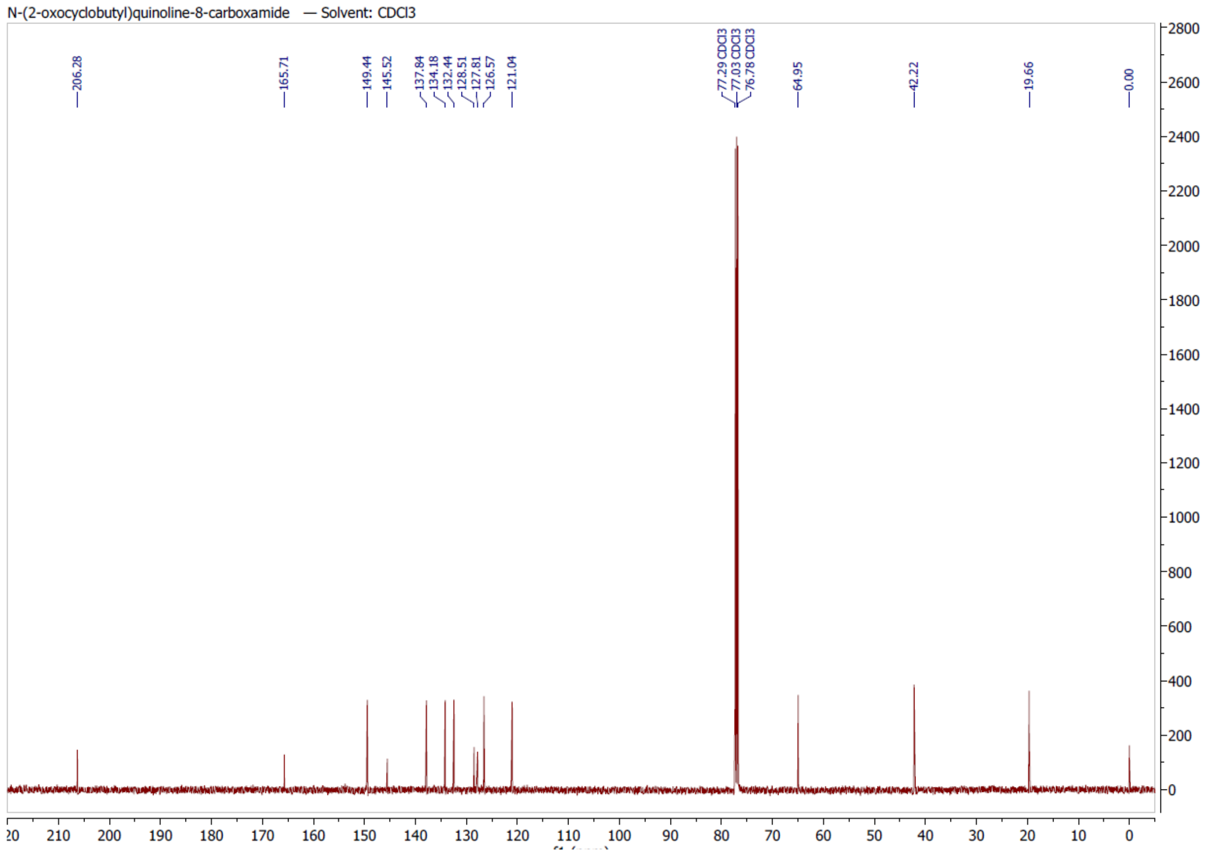

Figure S36. ^{13}C NMR (126 MHz, $CDCl_3$) of *N*-(2-oxocyclobutyl)quinoline-8-carboxamide (**3r**).

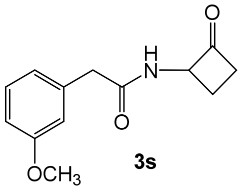

3s

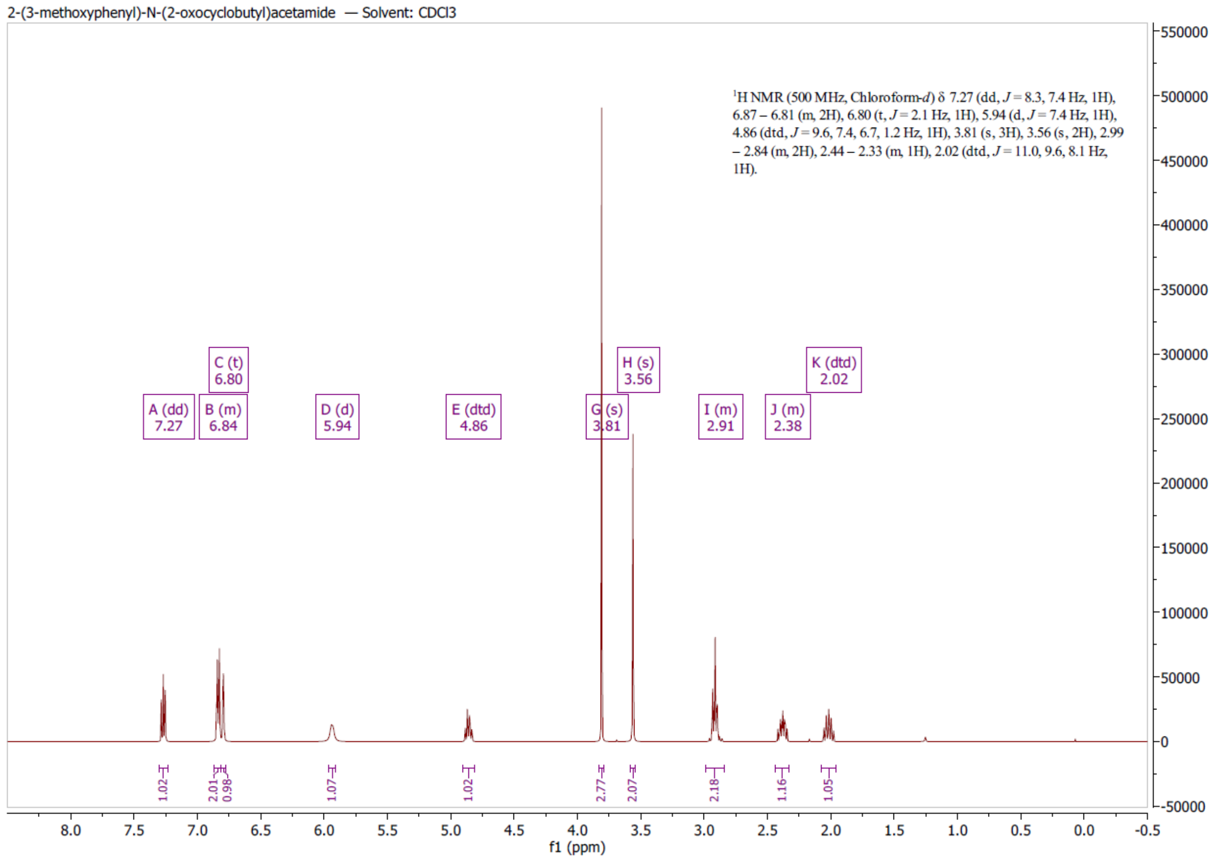

Figure S37. ^{1}H NMR (500 MHz $CDCl_3$) of 2-(3-methoxyphenyl)-*N*-(2-oxocyclobutyl)acetamide (**3s**).

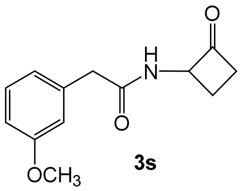

3s

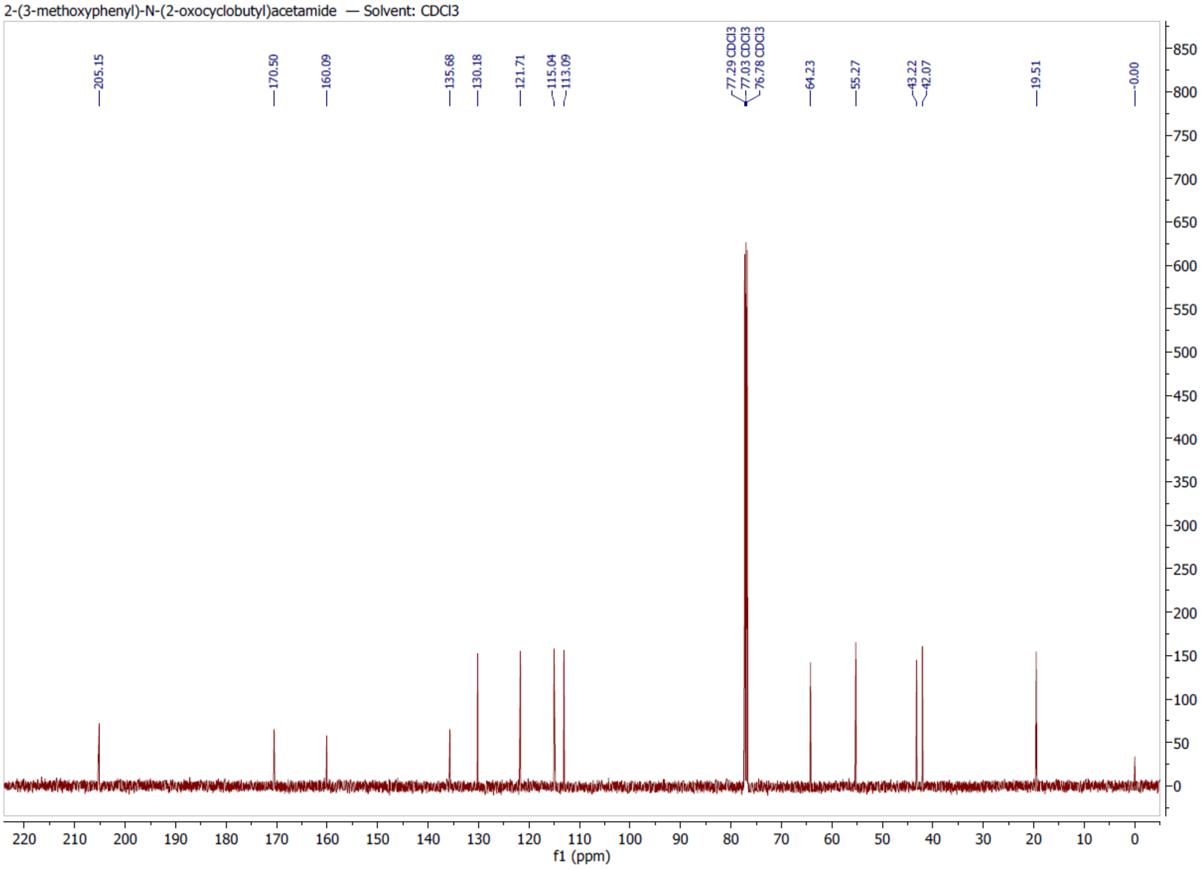

Figure S38. ^{13}C NMR (126 MHz, $CDCl_3$) of 2-(3-methoxyphenyl)-*N*-(2-oxocyclobutyl)acetamide (**3s**).

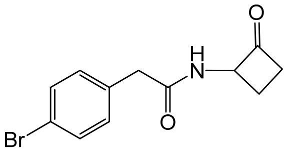

3t

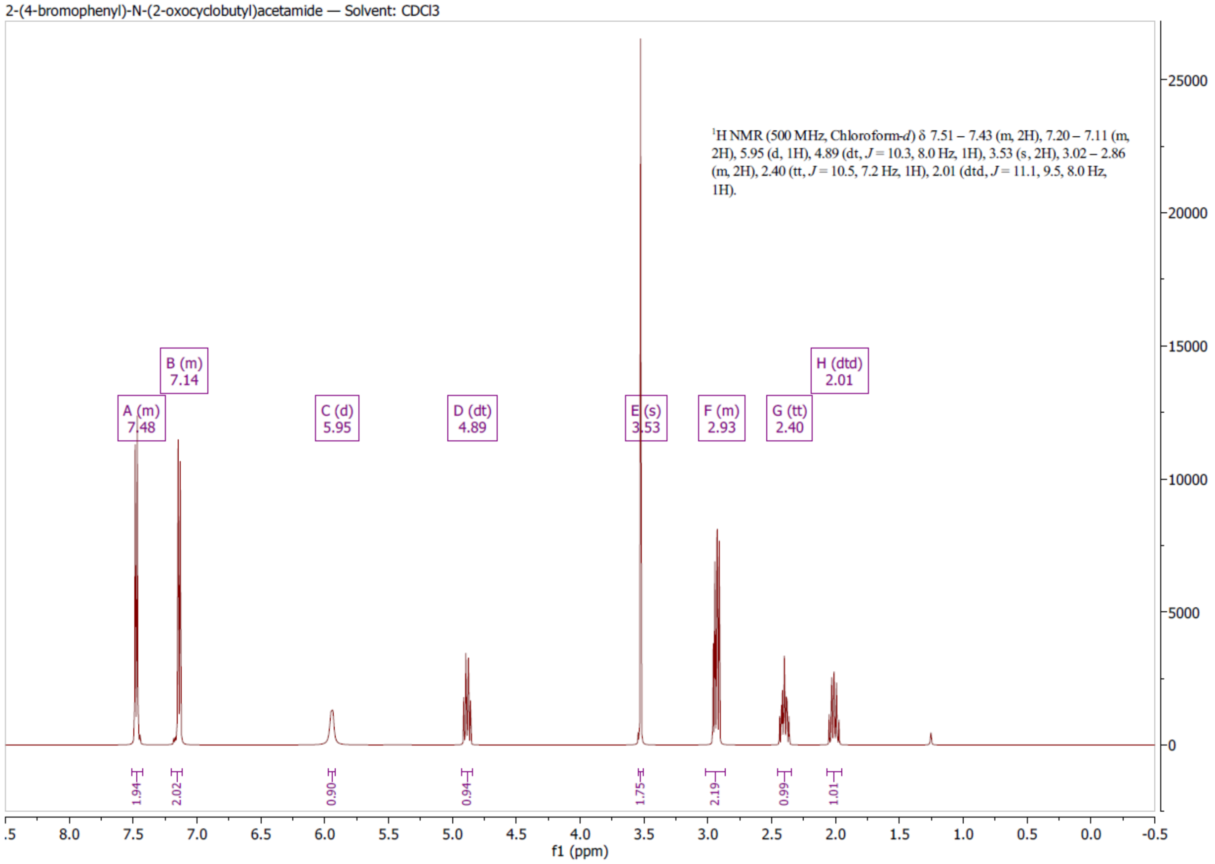

Figure S39. ^{1}H NMR (500 MHz $CDCl_3$) of 2-(4-bromophenyl)-*N*-(2-oxocyclobutyl)acetamide (**3t**).

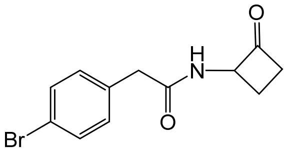

3t

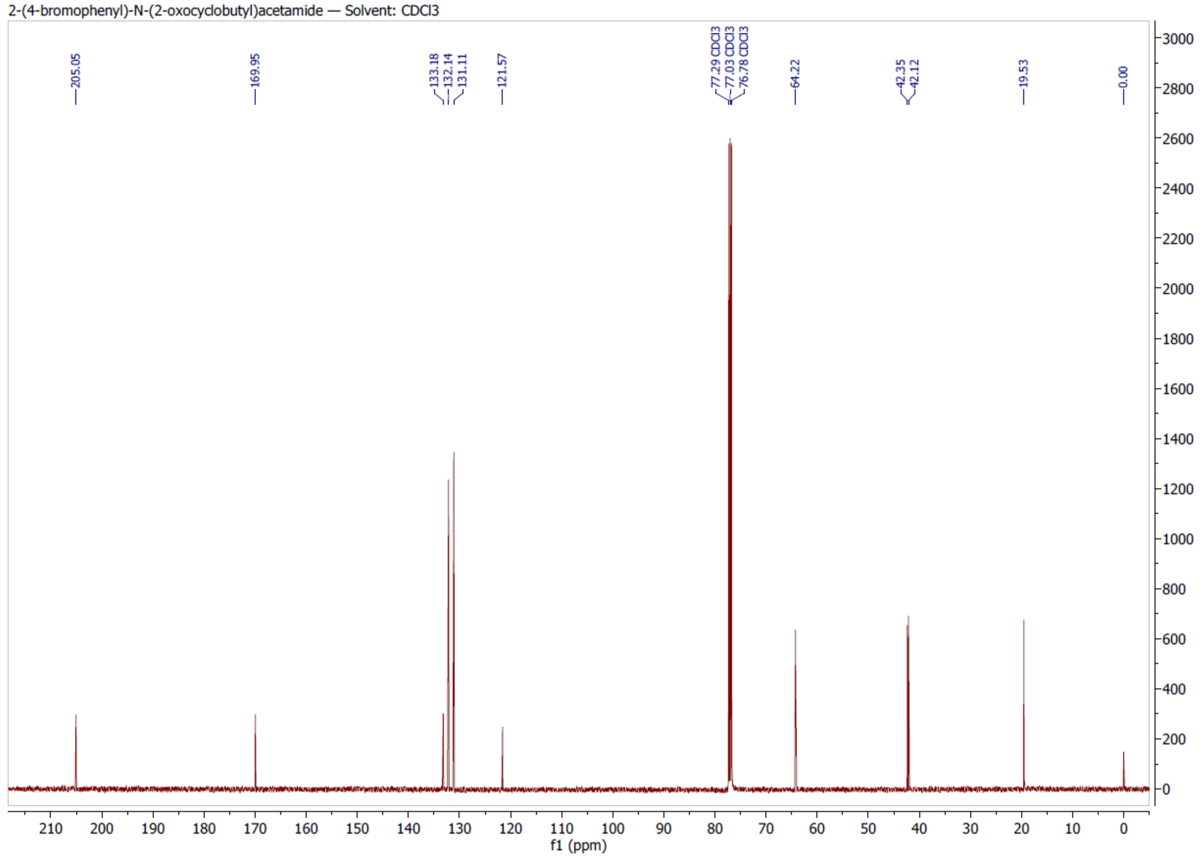

Figure S40. ^{13}C NMR (126 MHz, $CDCl_3$) of 2-(4-bromophenyl)-*N*-(2-oxocyclobutyl)acetamide (**3t**).

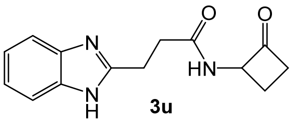

3u

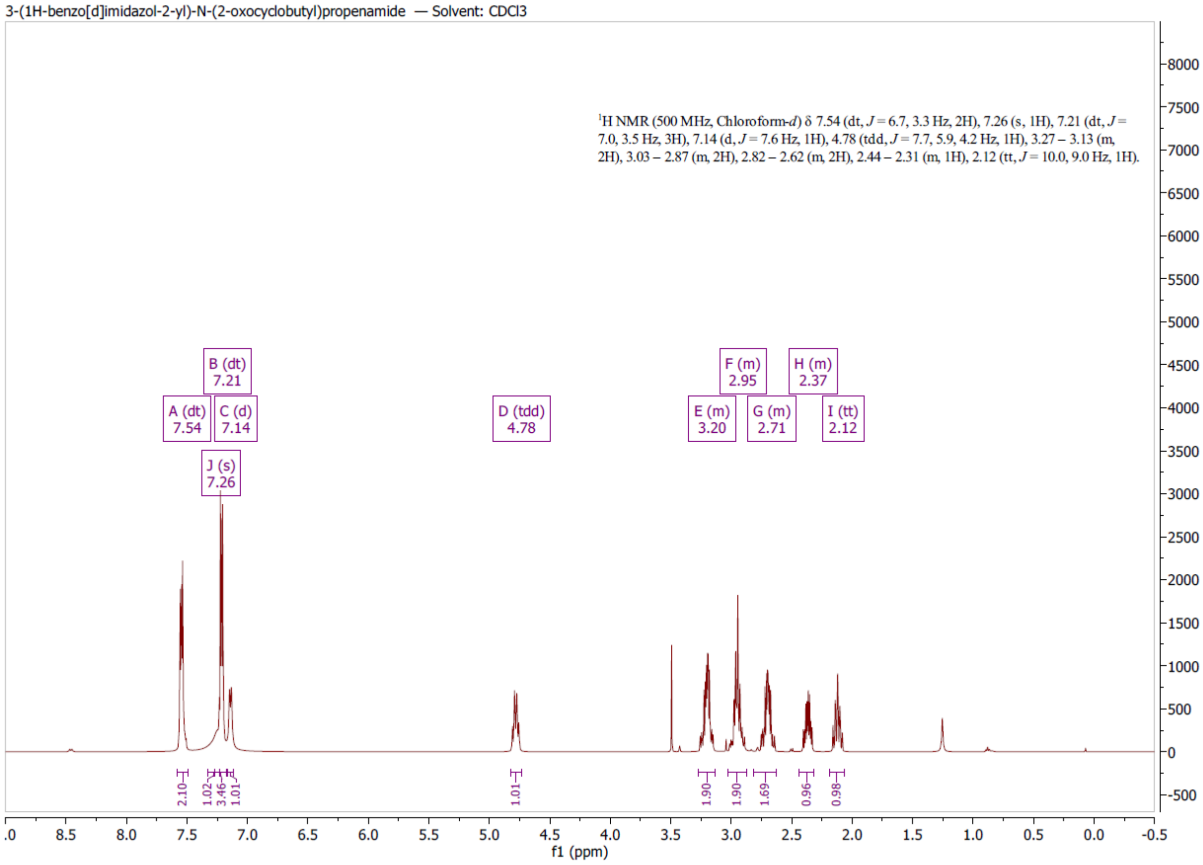

Figure S41. ^{1}H NMR (500 MHz $CDCl_3$) of 3-(*1H*-benzo[*d*]imidazol-2-yl)-*N*-(2-oxocyclobutyl)propenamide (**3u**).

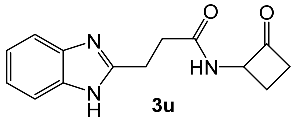

3u

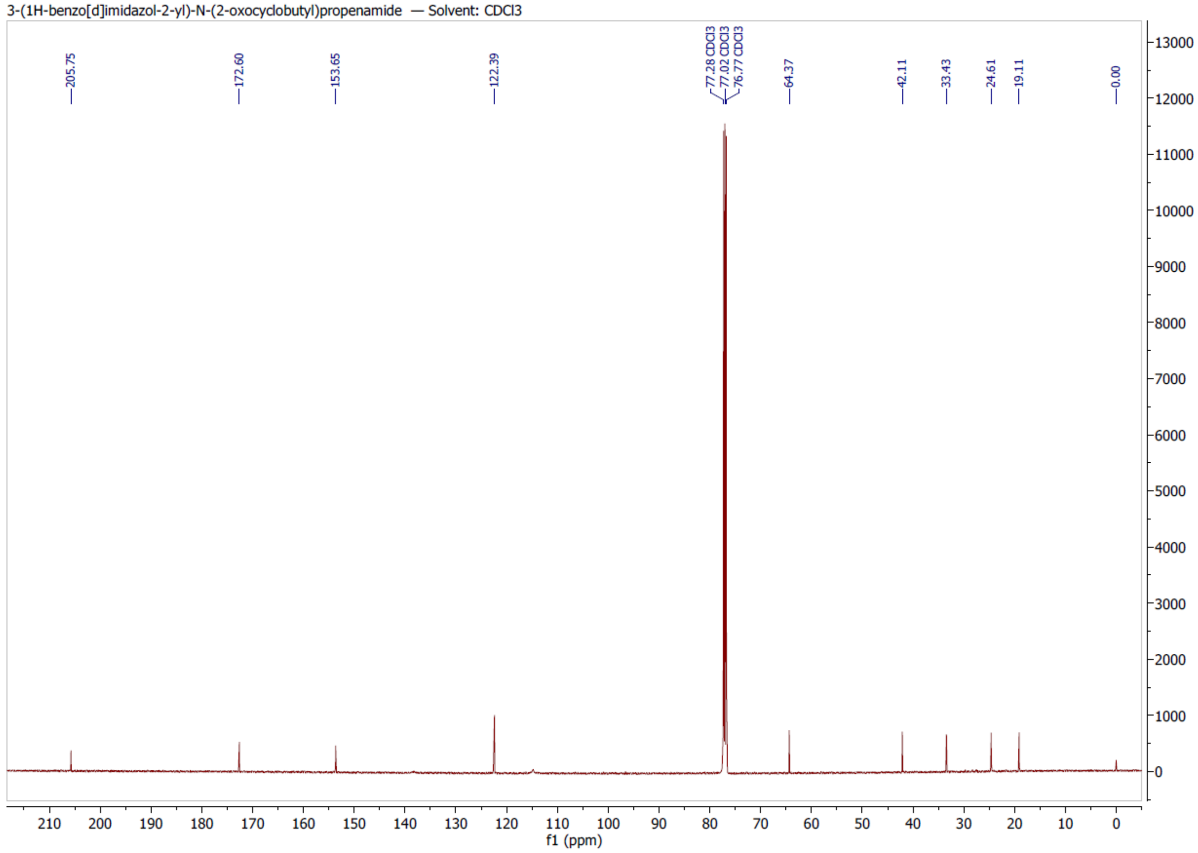

Figure S42. ^{13}C NMR (126 MHz, $CDCl_3$) of 3-(*1H*-benzo[*d*]imidazol-2-yl)-*N*-(2-oxocyclobutyl)propenamide (**3u**).

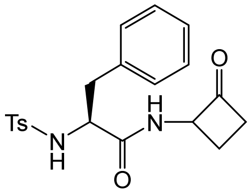

3v

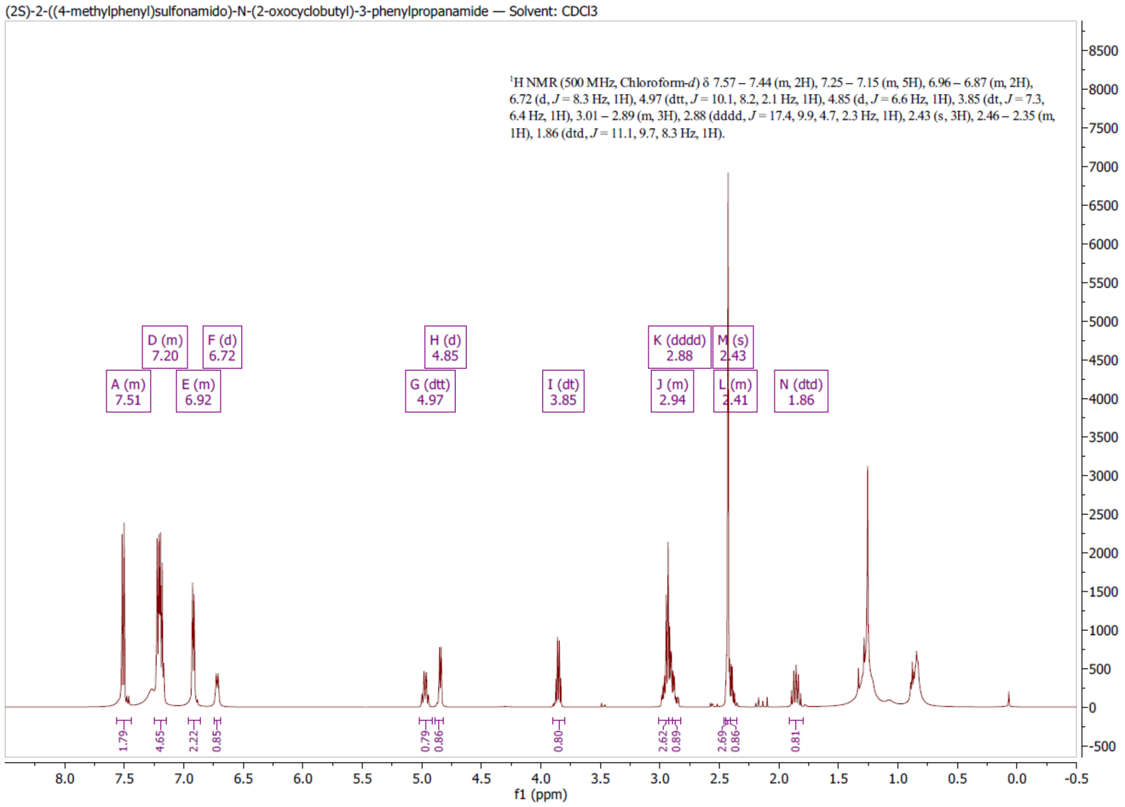

Figure S43. ^{1}H NMR (500 MHz $CDCl_3$) of (2*S*)-2-((4-methylphenyl)sulfonamido)-*N*-(2-oxocyclobutyl)-3-phenylpropanamide (**3v**).

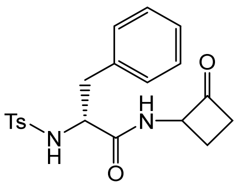

3w

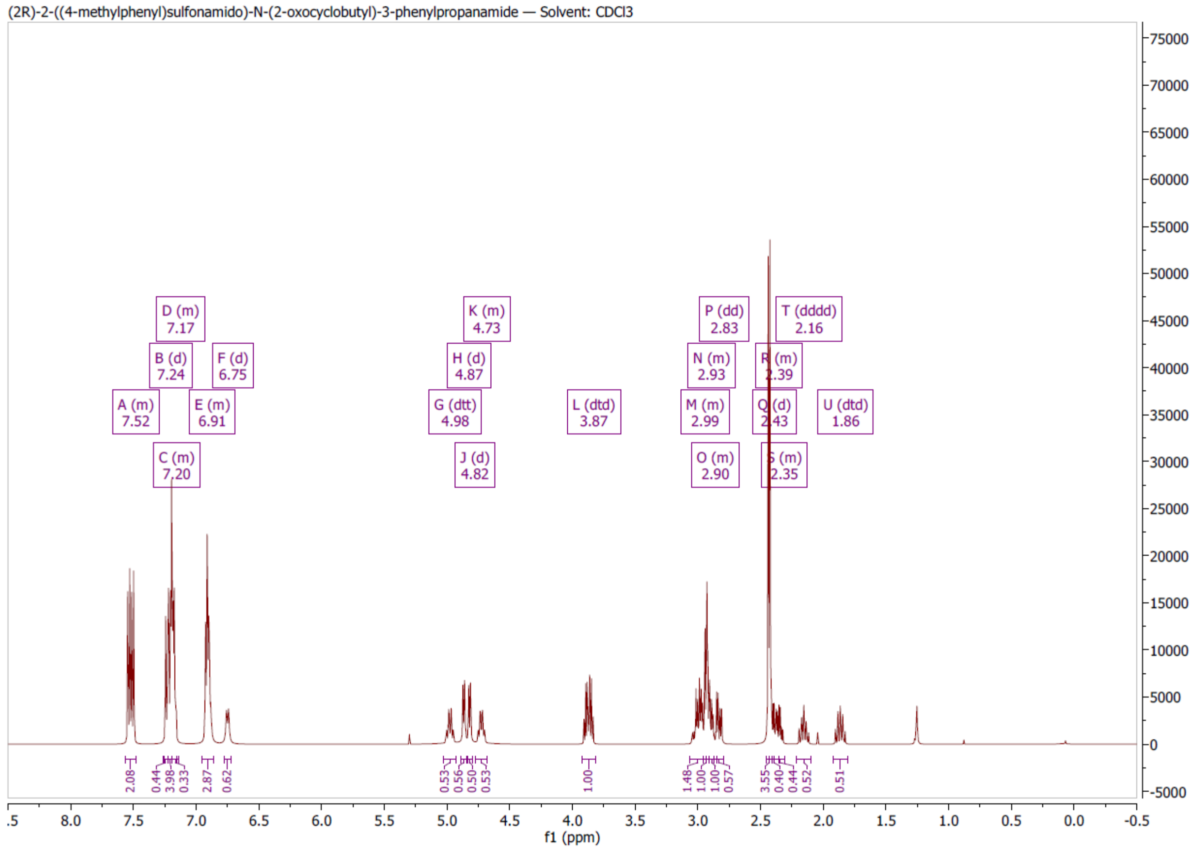

Figure S44. ^{1}H NMR (500 MHz $CDCl_3$) of (*2R*)-2-((4-methylphenyl)sulfonamido)-*N*-(2-oxocyclobutyl)-3-phenylpropanamide (**3w**).

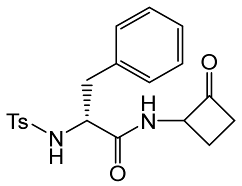

3w

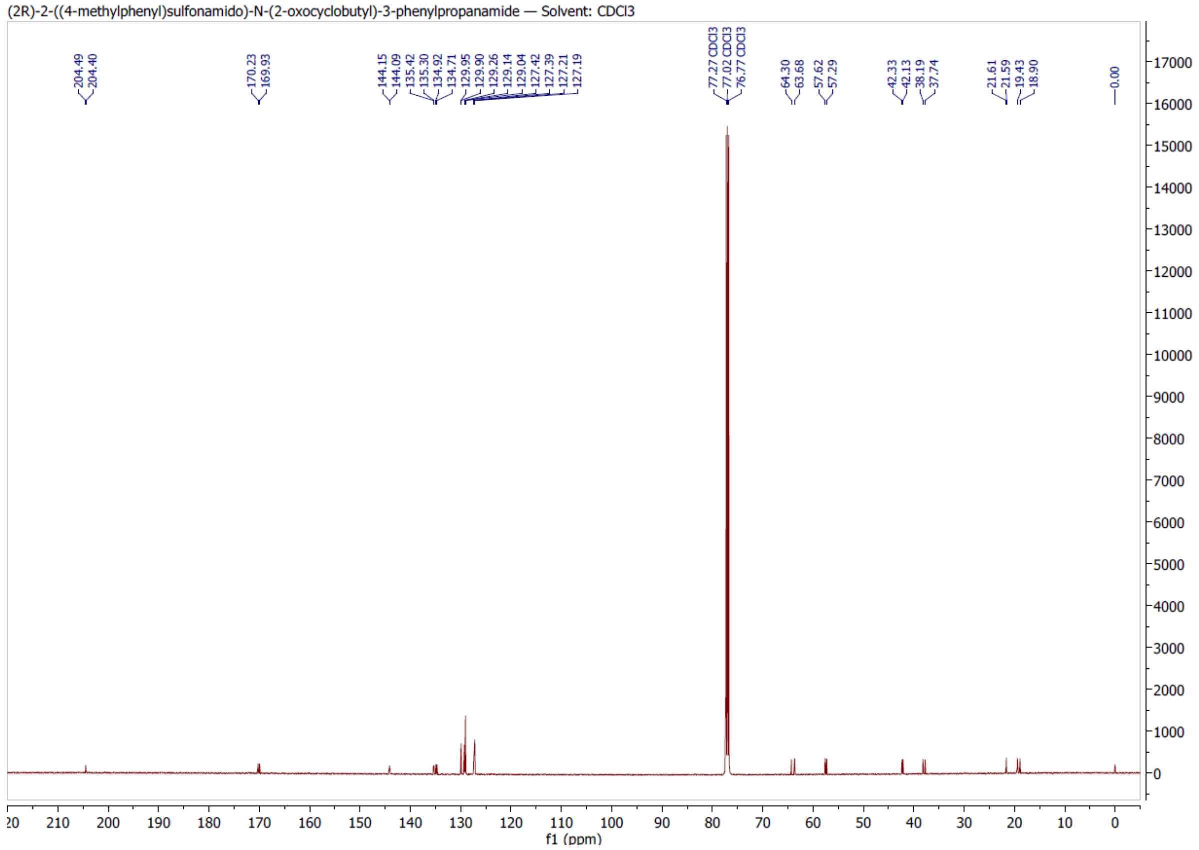

Figure S45. ^{13}C NMR (126 MHz, $CDCl_3$) of (*2R*)-2-((4-methylphenyl)sulfonamido)-*N*-(2-oxocyclobutyl)-3-phenylpropanamide (**3w**).

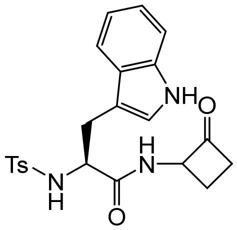

3x

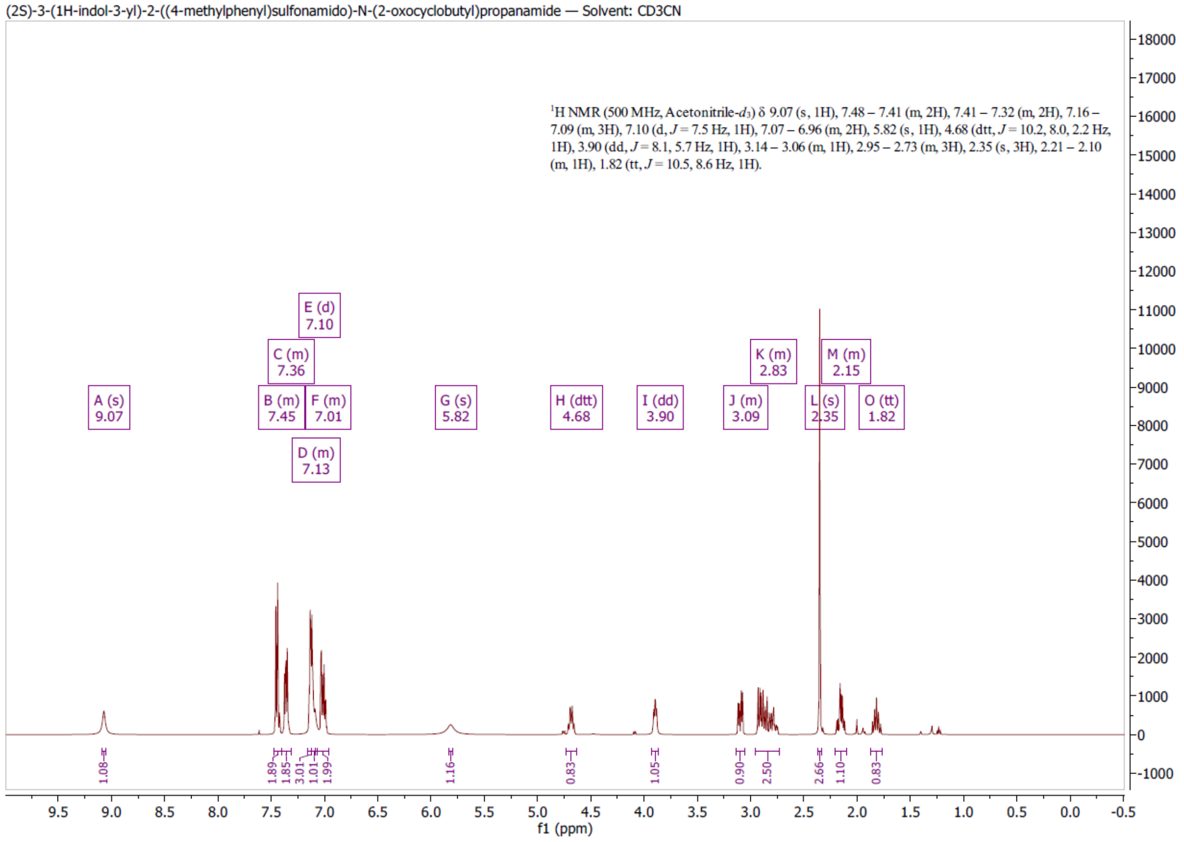

Figure S46. ^{1}H NMR (500 MHz $CDCl_3$) of (2*S*)-3-(1*H*-indol-3-yl)-2-((4-methylphenyl)sulfonamido)-*N*-(2-oxocyclobutyl)propanamide (**3x**).

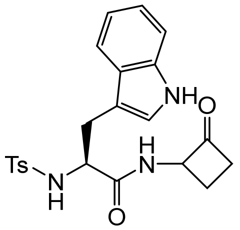

3x

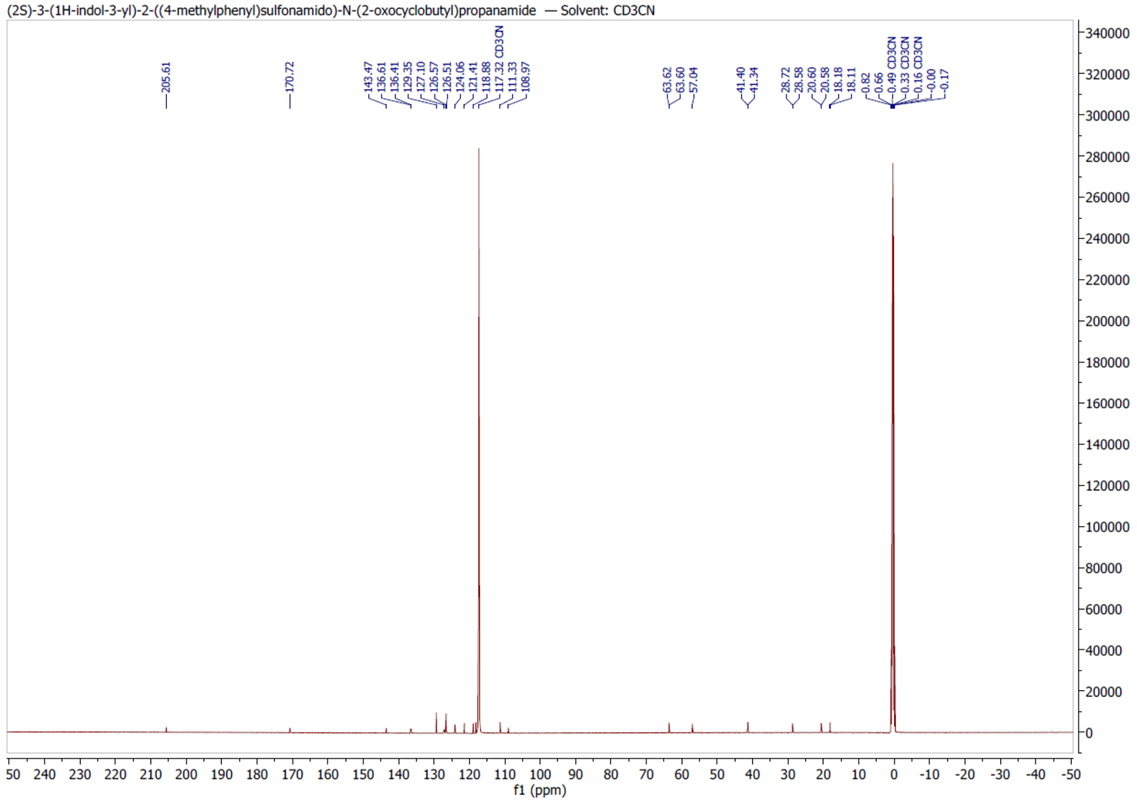

Figure S47. ^{13}C NMR (126 MHz, $CDCl_3$) of (2*S*)-3-(1*H*-indol-3-yl)-2-((4-methylphenyl)sulfonamido)-*N*-(2-oxocyclobutyl)propanamide (**3x**).

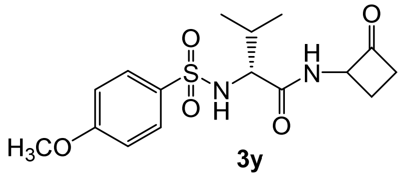

3y

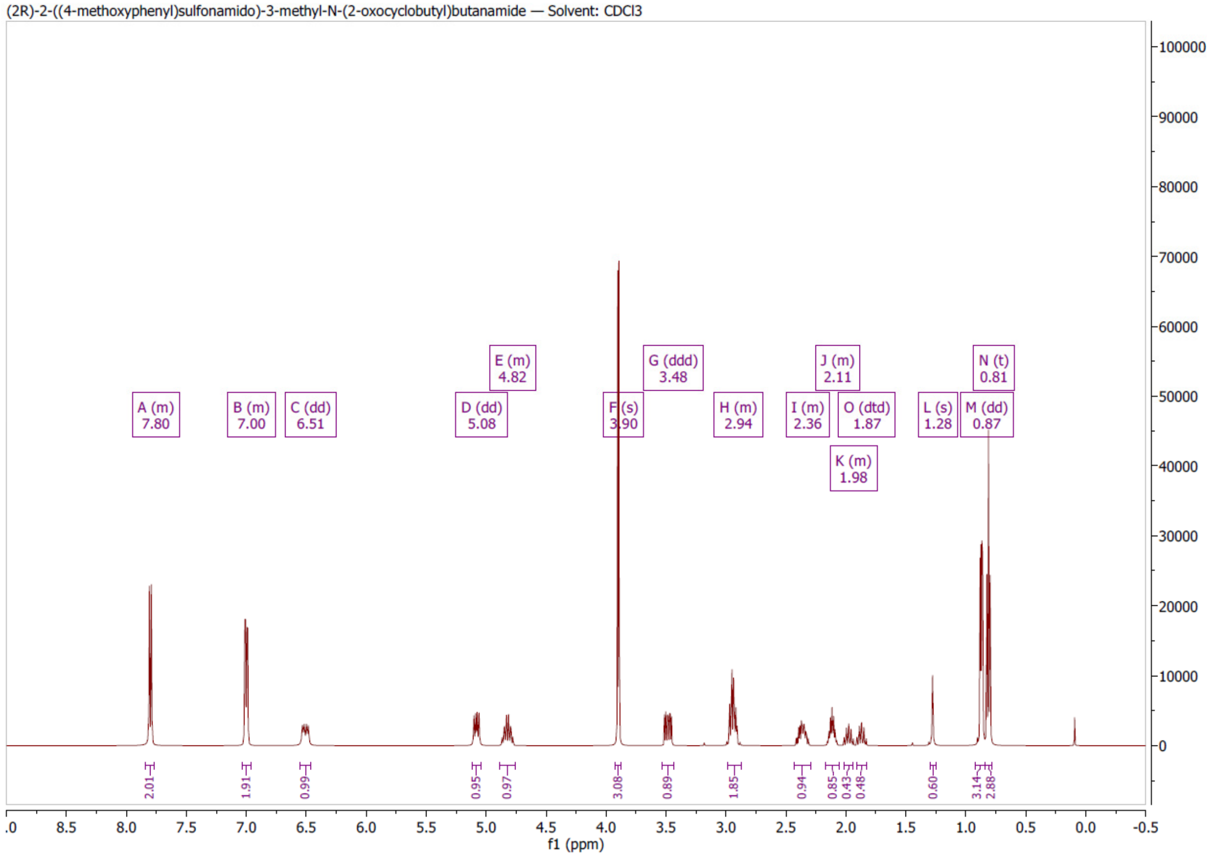

Figure S48. ^{1}H NMR (500 MHz $CDCl_3$) of (2*R*)-2-((4-methoxyphenyl)sulfonamido)-3-methyl-*N*-(2-oxocyclobutyl)butanamide (**3y**).

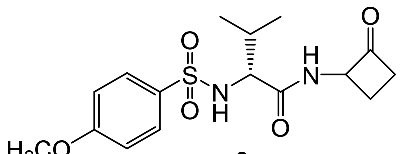

3y

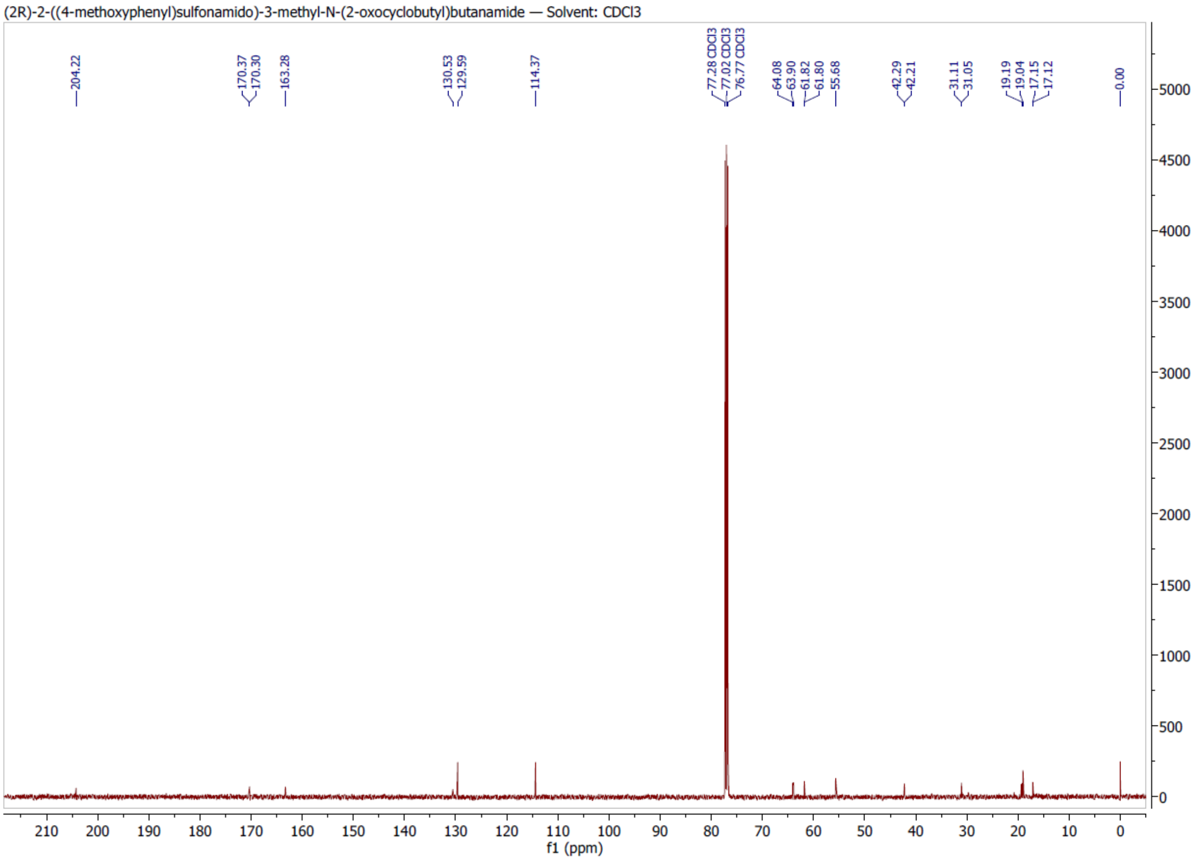

Figure S49. ^{13}C NMR (126 MHz, $CDCl_3$) of (*2R*)-2-((4-methoxyphenyl)sulfonamido)-3-methyl-*N*-(2-oxocyclobutyl)butanamide (**3y**).

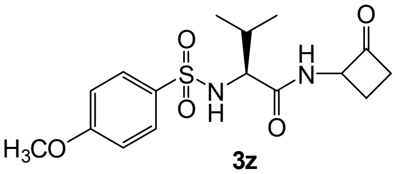

3z

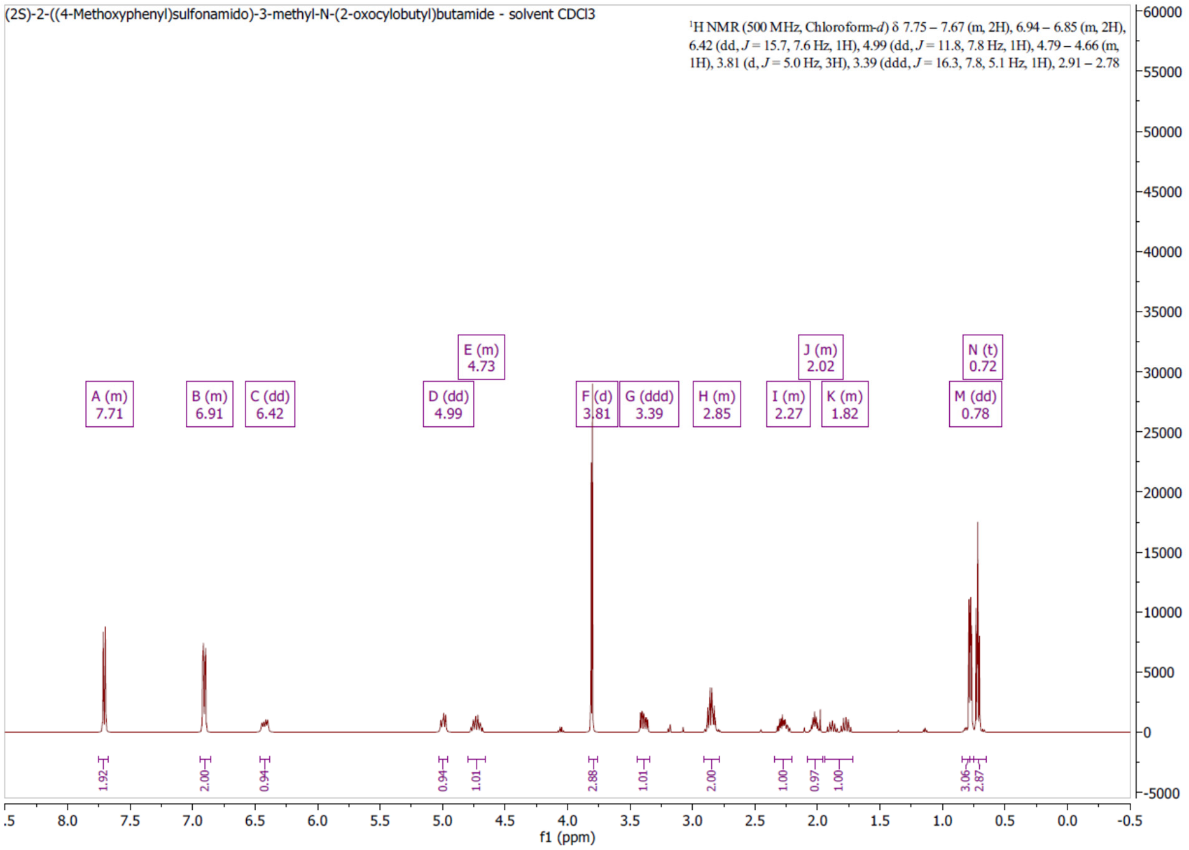

Figure S50. ^{1}H NMR (500 MHz $CDCl_3$) of (*2S*)-2-((4-methoxyphenyl)sulfonamido)-3-methyl-*N*-(2-oxocyclobutyl)butanamide (**3z**).

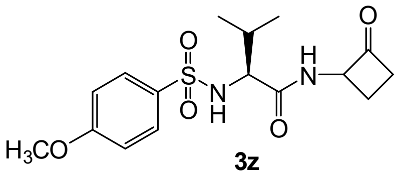

3z

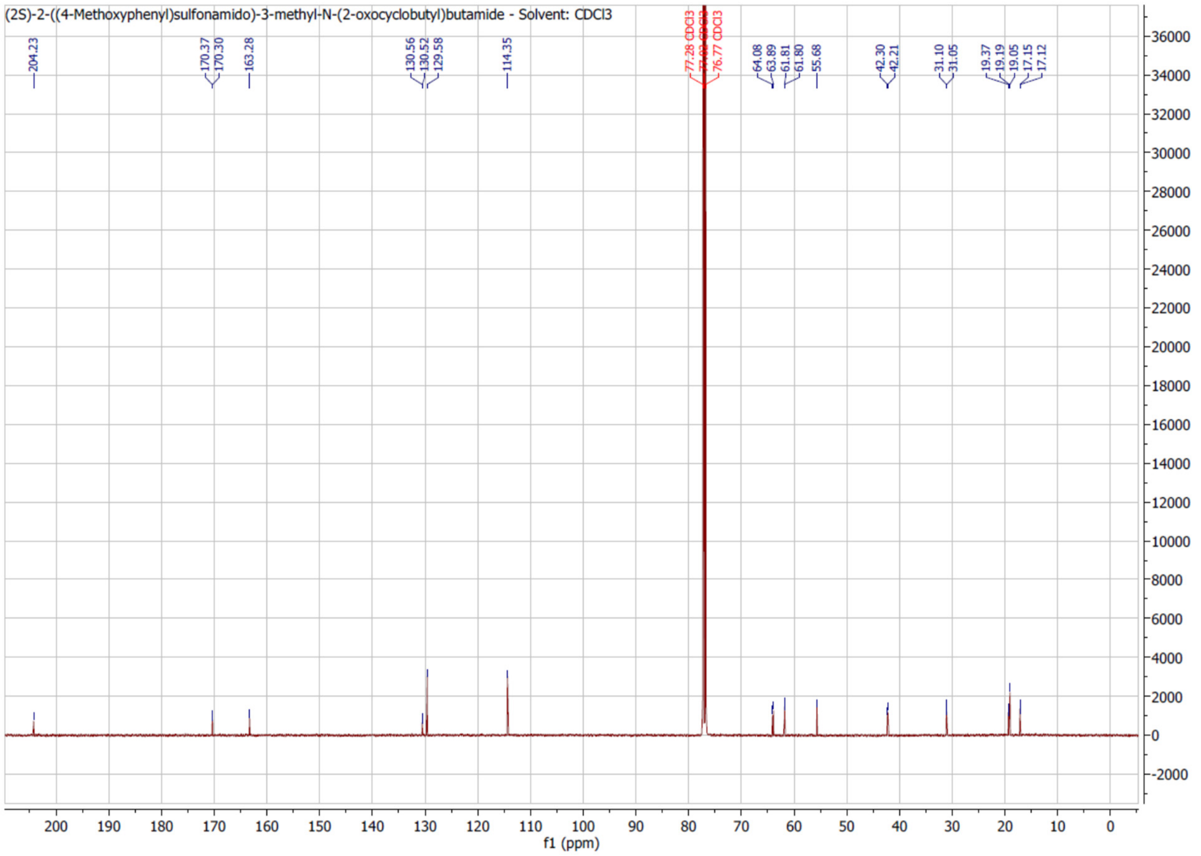

Figure S51. ^{13}C NMR (126 MHz, $CDCl_3$) of (2*S*)-2-((4-methoxyphenyl)sulfonamido)-3-methyl-*N*-(2-oxocyclobutyl)butanamide (**3z**).

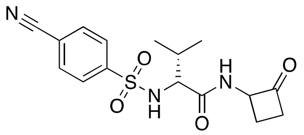

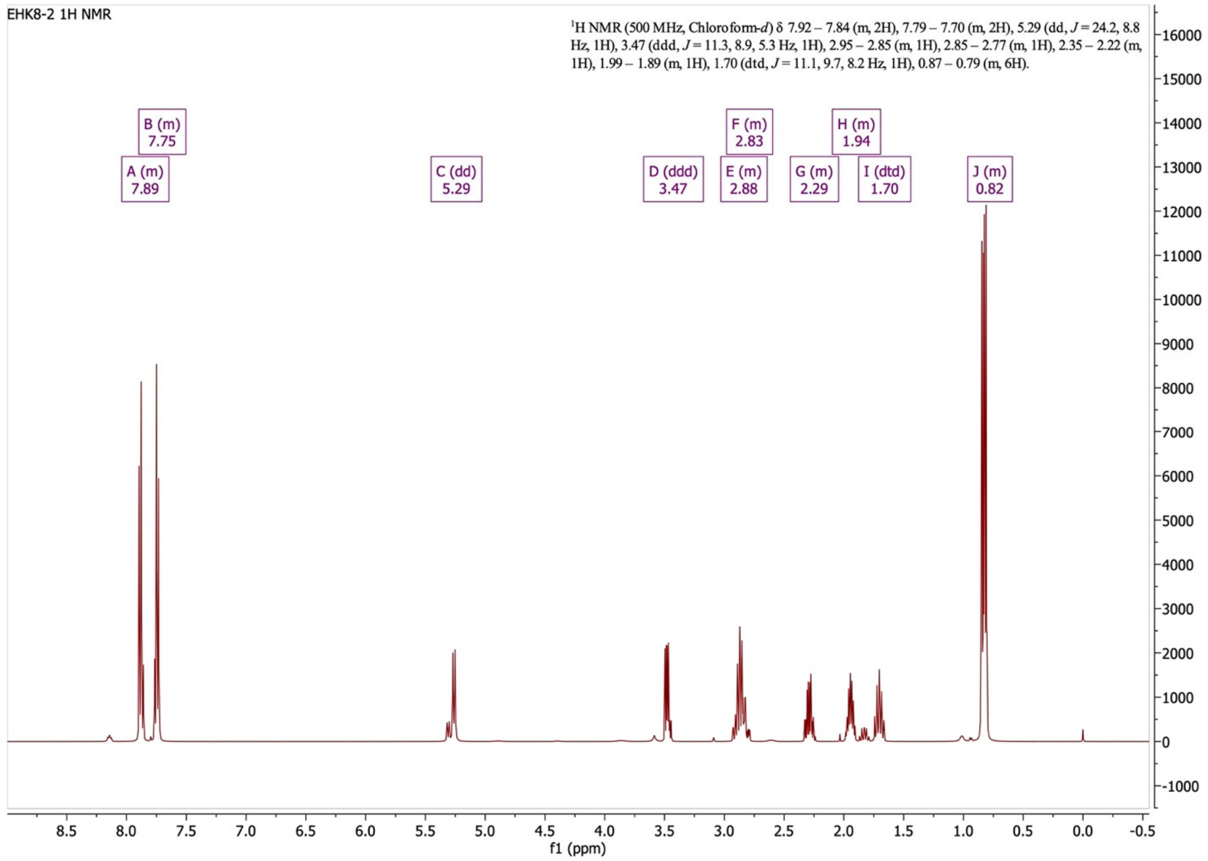

Figure S52. ^{1}H NMR (500 MHz $CDCl_3$) of (2R)-2-((4-cyanophenyl)sulfonamido)-3-methyl-N-(2-oxocyclobutyl)butanamide **(3aa).**

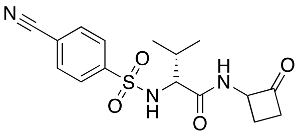

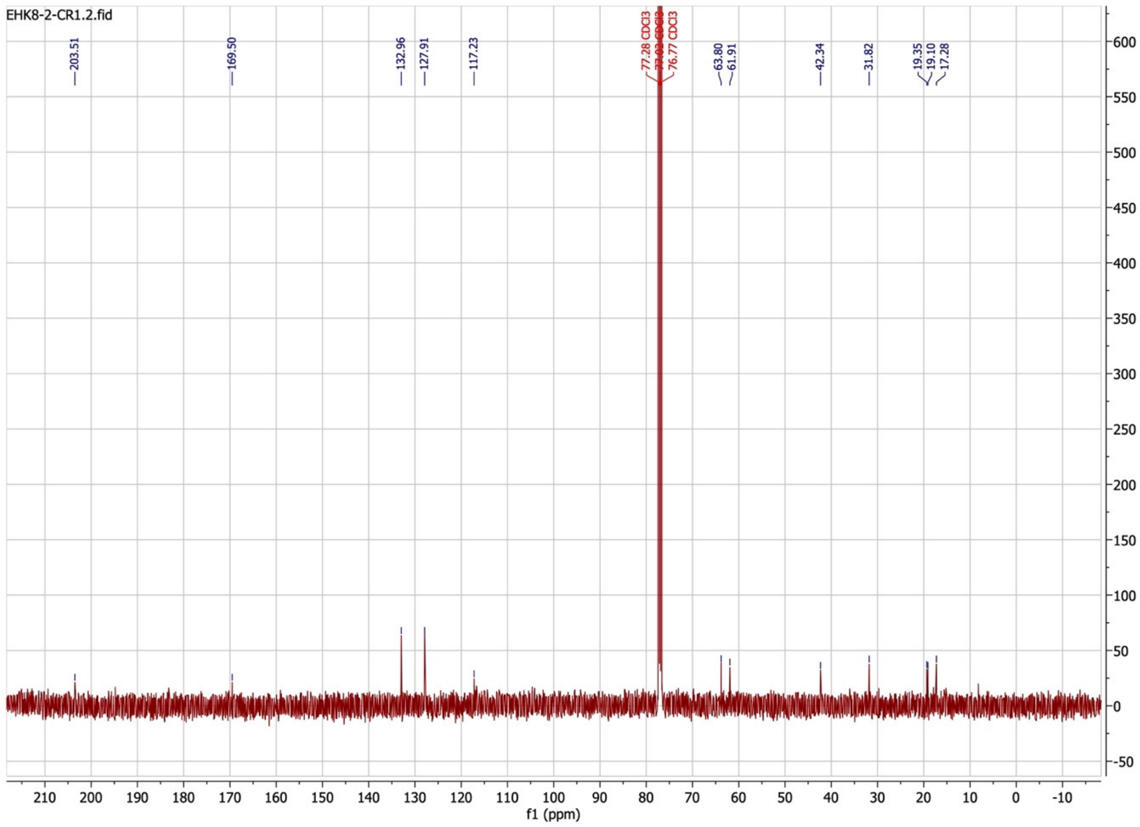

Figure S53. ^{13}C NMR (126 MHz, $CDCl_3$) of (2R)-2-((4-cyanophenyl)sulfonamido)-3-methyl-N-(2-oxocyclobutyl)butanamide **(3aa).**

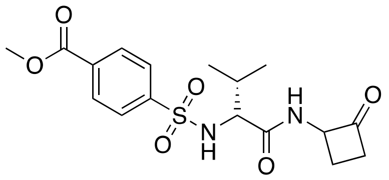

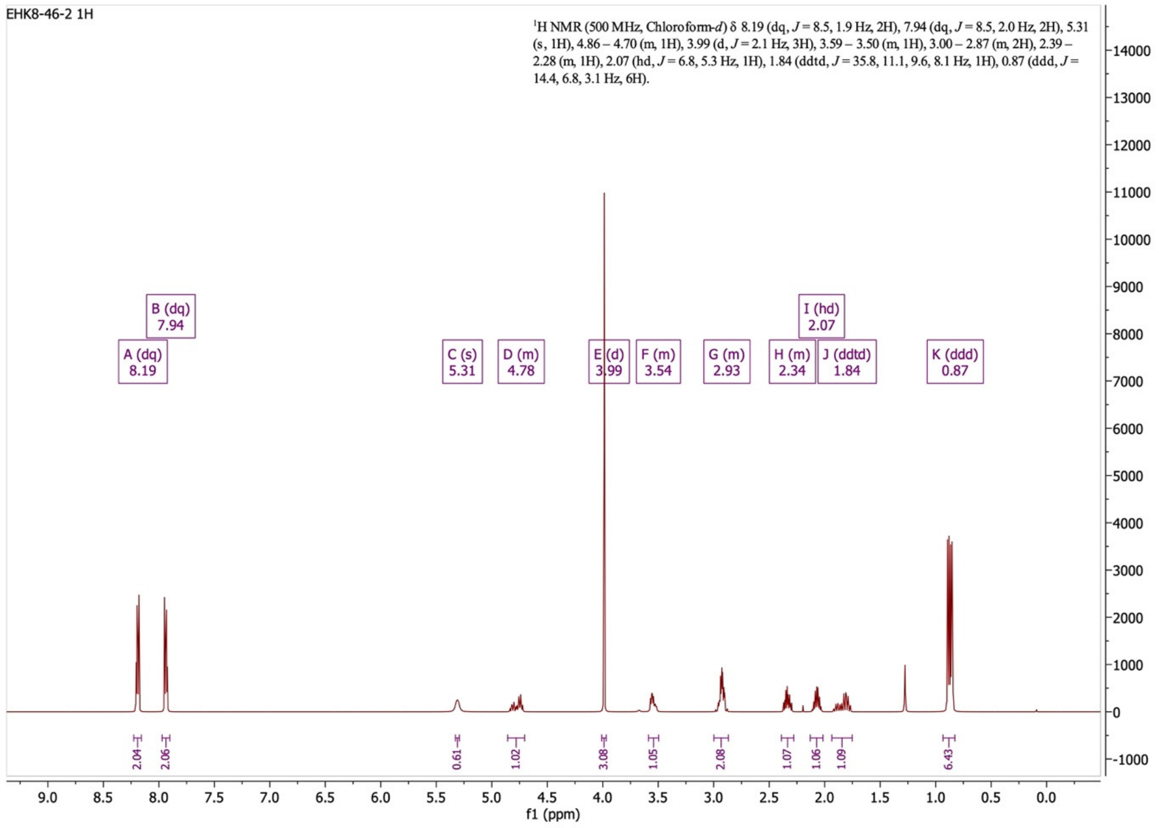

Figure S54. ^{1}H NMR (500 MHz $CDCl_3$) of methyl 4-(N-((2R)-3-methyl-1-oxo-1-((2-oxocyclobutyl)amino)butan-2-yl)sulfamoyl)benzoate **(3ab)**.

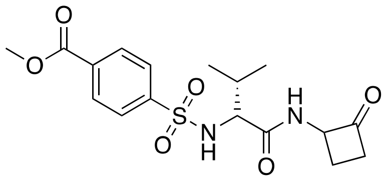

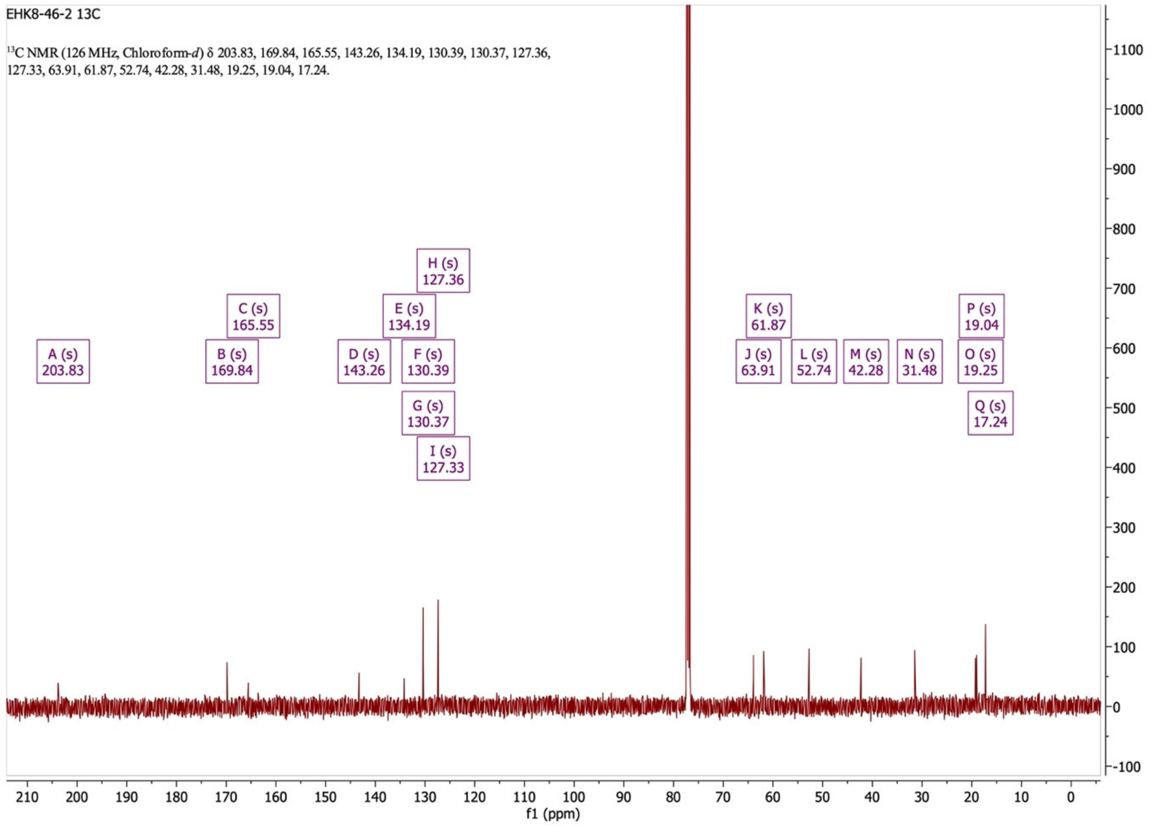

Figure S55. ^{13}C NMR (126 MHz, $CDCl_3$) of methyl 4-(N-((2R)-3-methyl-1-oxo-1-((2-oxocyclobutyl)amino)butan-2-yl)sulfamoyl)benzoate **(3ab)**.

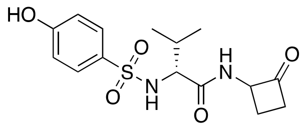

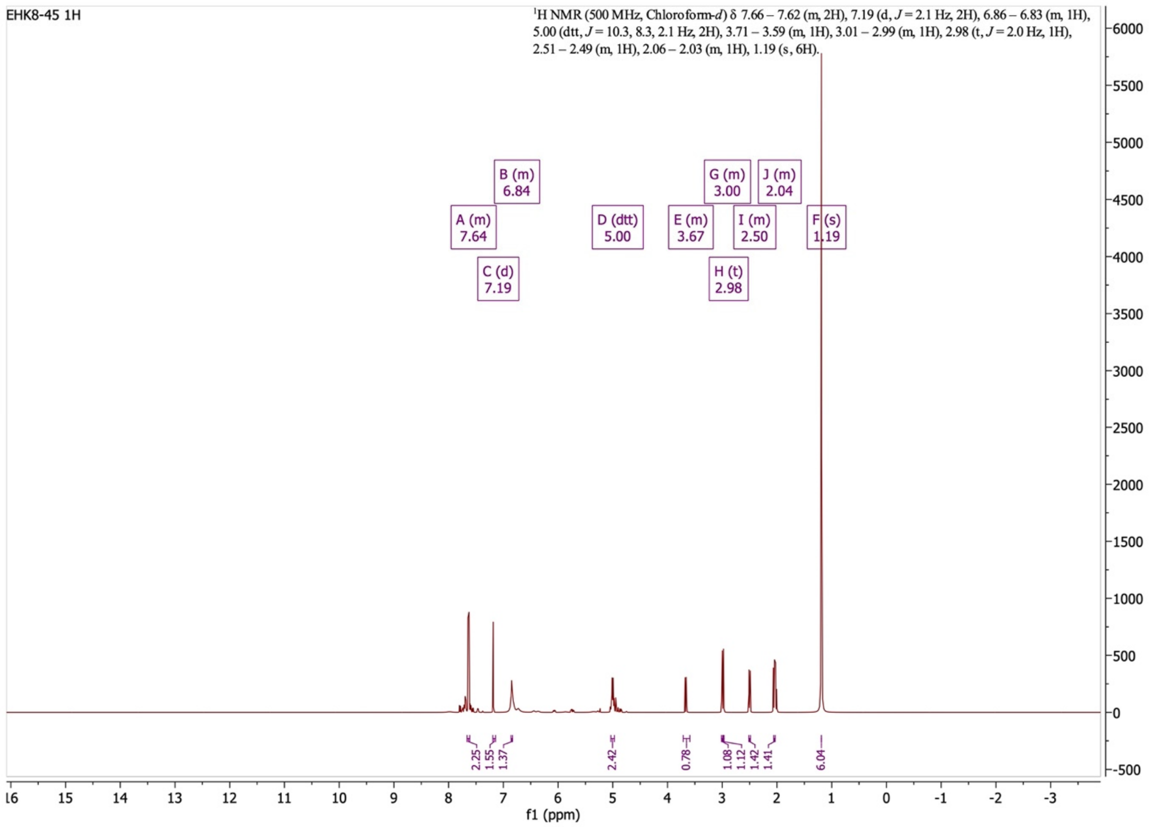

Fig, S56. ^{1}H NMR (500 MHz CDCl3) of (2R)-2-((4-hydroxyphenyl)sulfonamido)-3-methyl-N-(2-oxocyclobutyl)butanamide **(3ac)**.

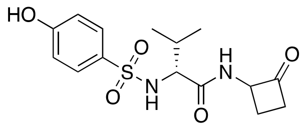

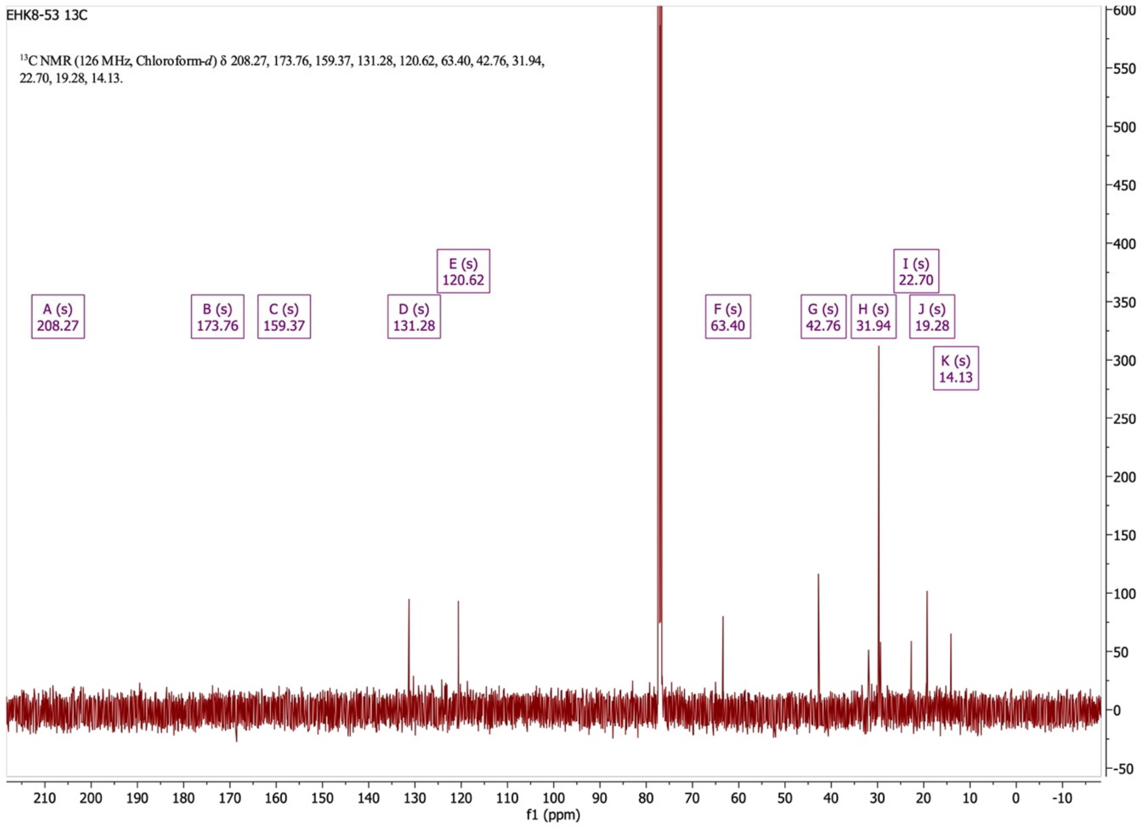

Figure S57. ^{13}C NMR (126 MHz, $CDCl_3$) of (2R)-2-((4-hydroxyphenyl)sulfonamido)-3-methyl-N-(2-oxocyclobutyl)butanamide **(3ac)**.

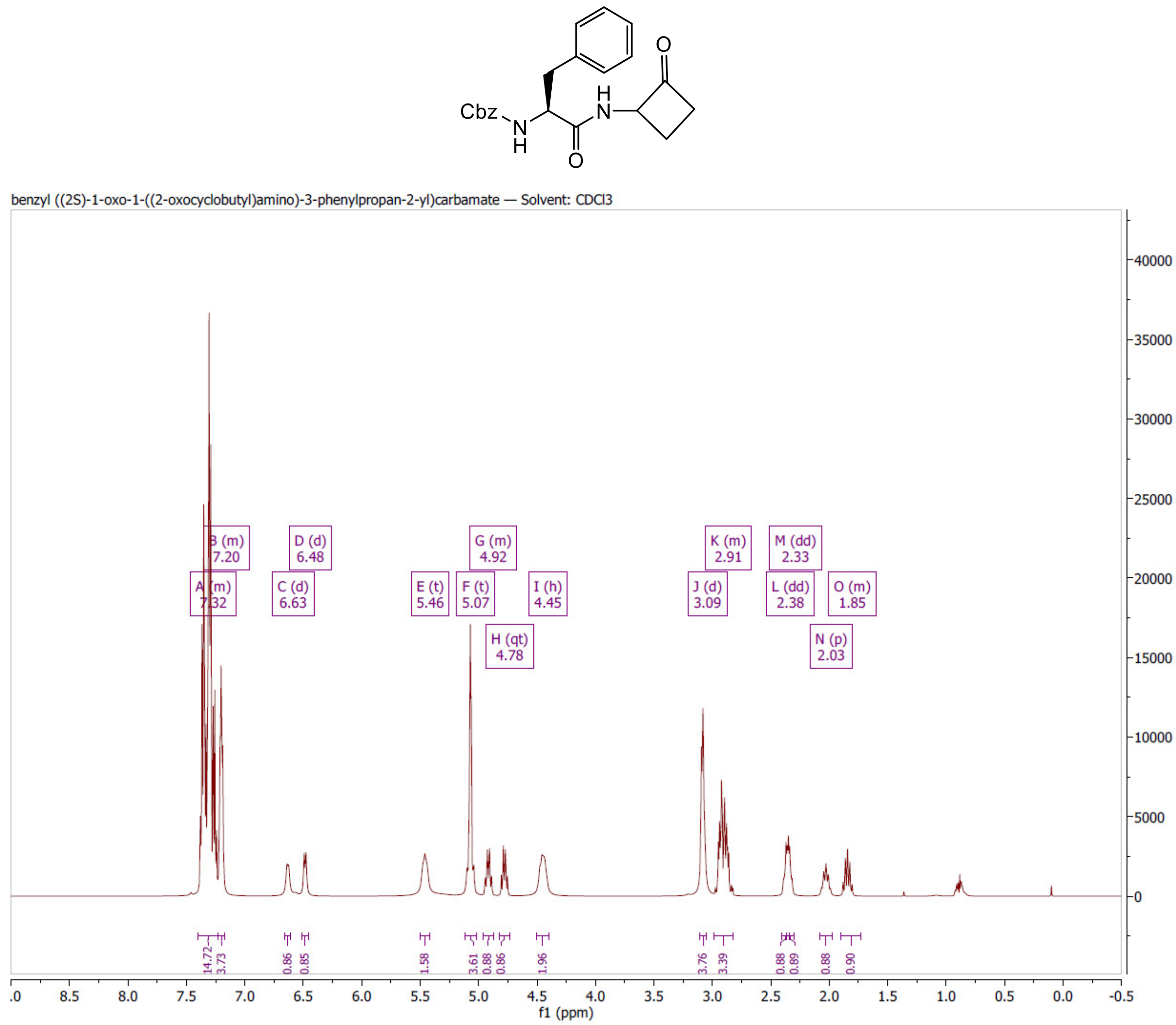

Figure S58. ^{1}H NMR (500 MHz $CDCl_3$) of benzyl ((2*S*)-1-oxo-1-((2-oxocyclobutyl)amino)-3-phenylpropan-2-yl)carbamate (**3ad**).

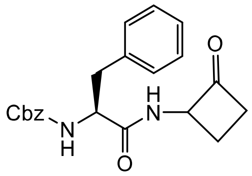

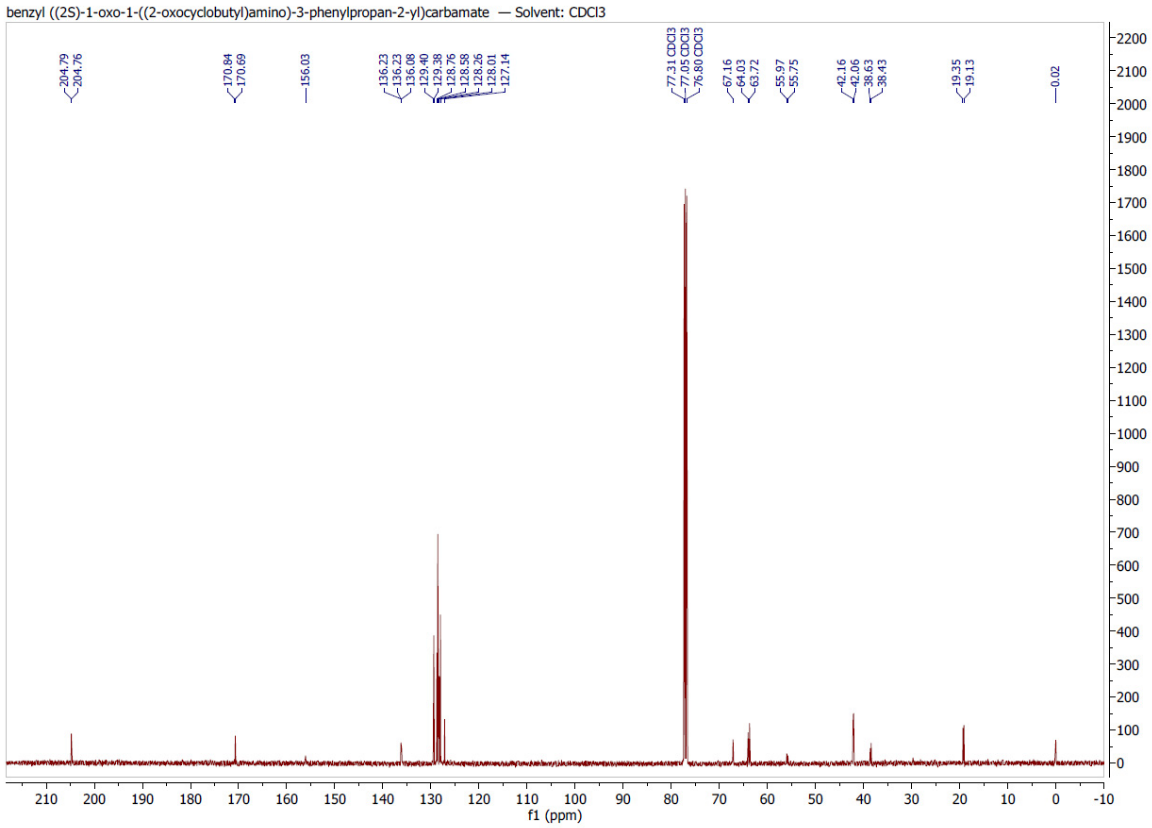

Figure S59. ^{13}C NMR (126 MHz, $CDCl_3$) of benzyl ((2*S*)-1-oxo-1-((2-oxocyclobutyl)amino)-3-phenylpropan-2-yl)carbamate (**3ad**).

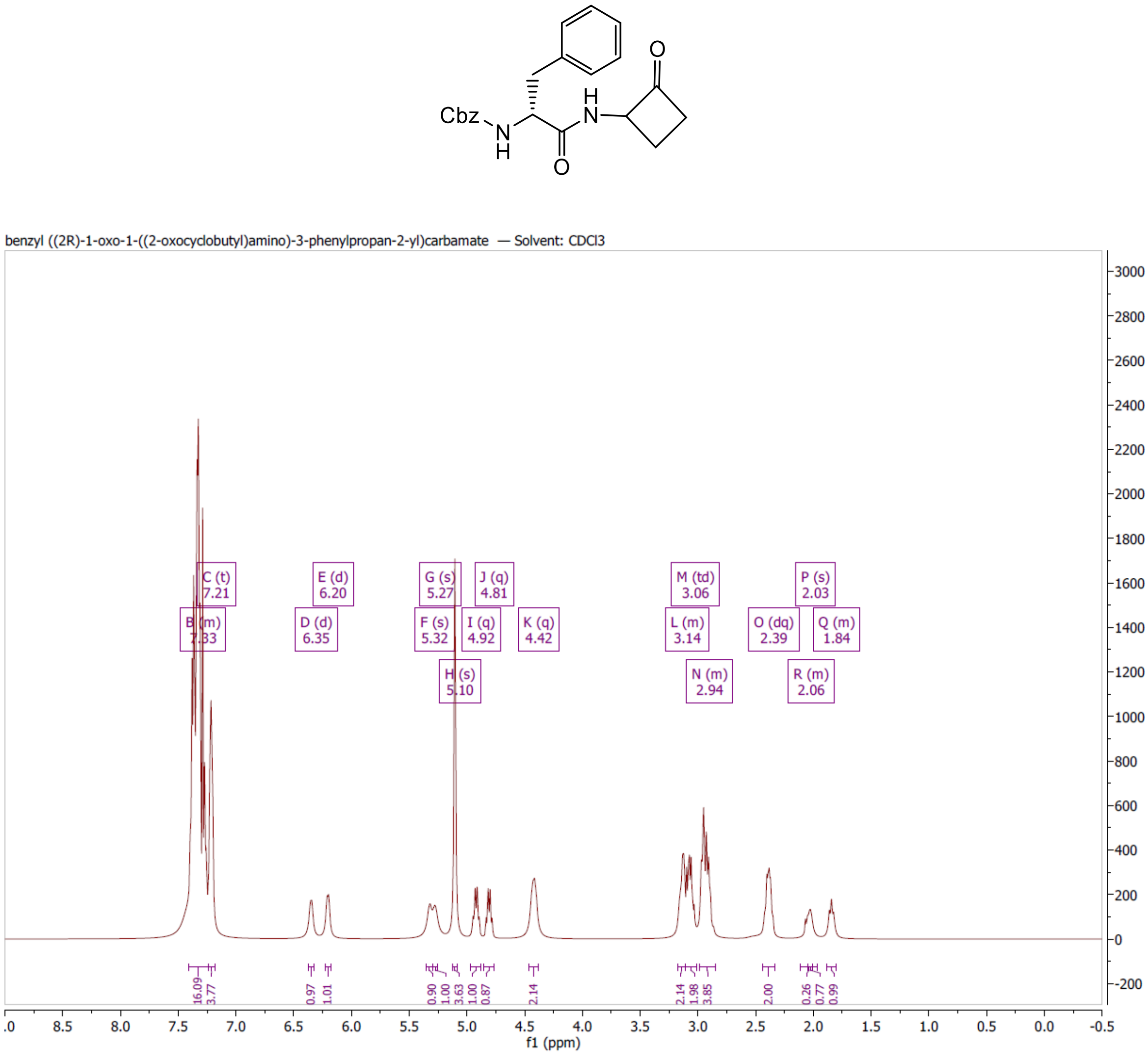

Figure S60. ^{1}H NMR (500 MHz $CDCl_3$) of benzyl ((2*R*)-1-oxo-1-((2-oxocyclobutyl)amino)-3-phenylpropan-2-yl)carbamate (**3ae**).

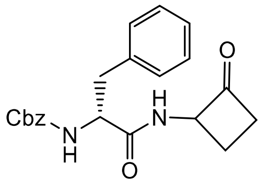

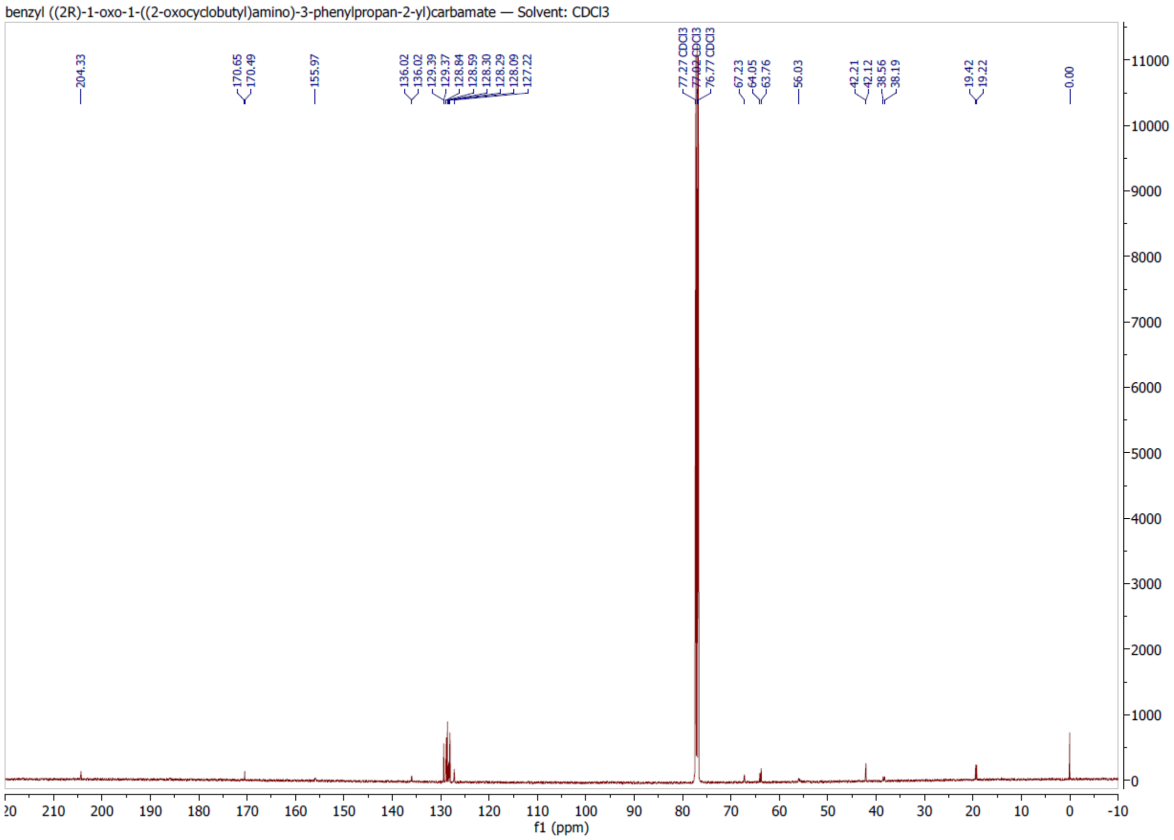

Figure S61. ^{13}C NMR (126 MHz, $CDCl_3$) of benzyl ((2*R*)-1-oxo-1-((2-oxocyclobutyl)amino)-3-phenylpropan-2-yl)carbamate (**3ae**).

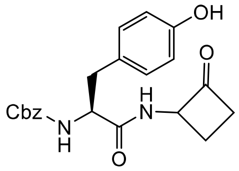

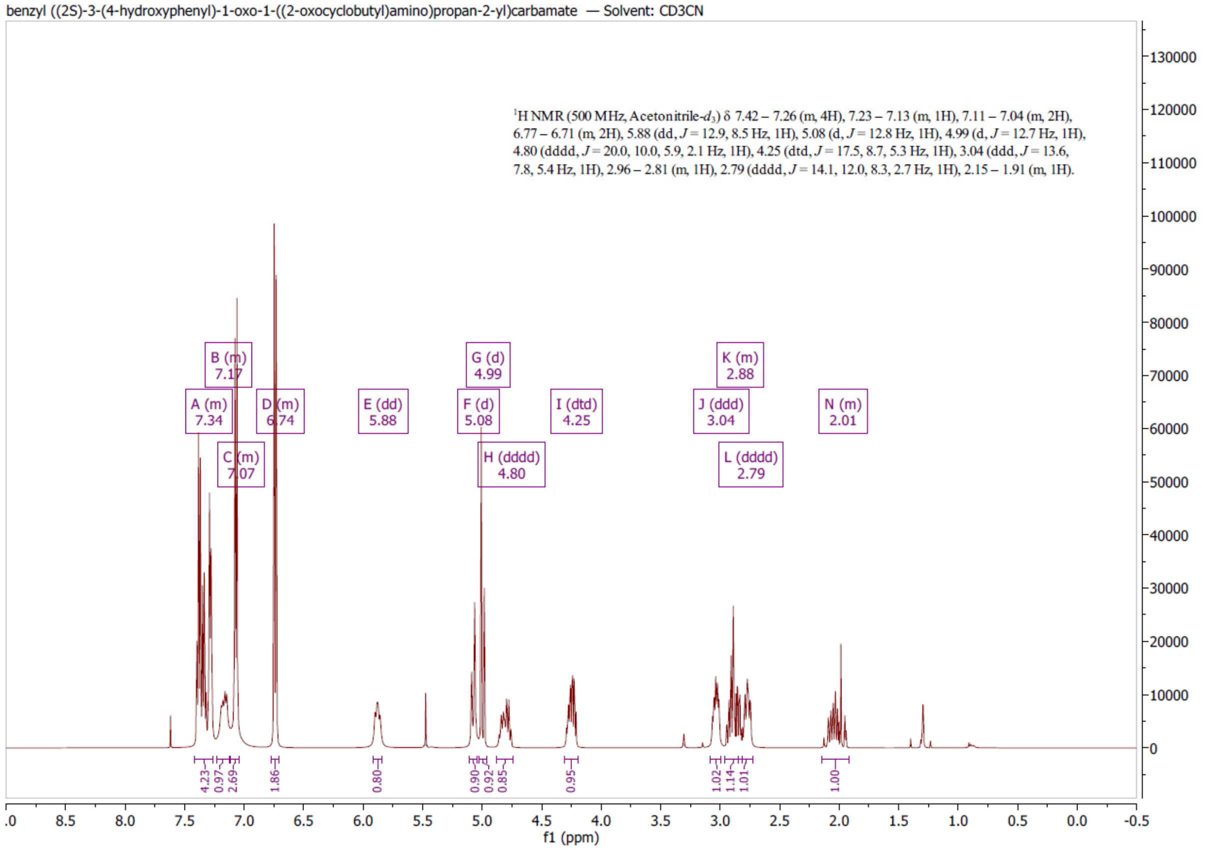

Figure S62. ^{1}H NMR (500 MHz $CDCl_3$) of benzyl ((*2S*)-3-(4-hydroxyphenyl)-1-oxo-1-((2-oxocyclobutyl)amino)propan-2-yl)carbamate (**3af**).

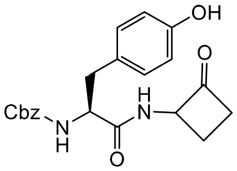

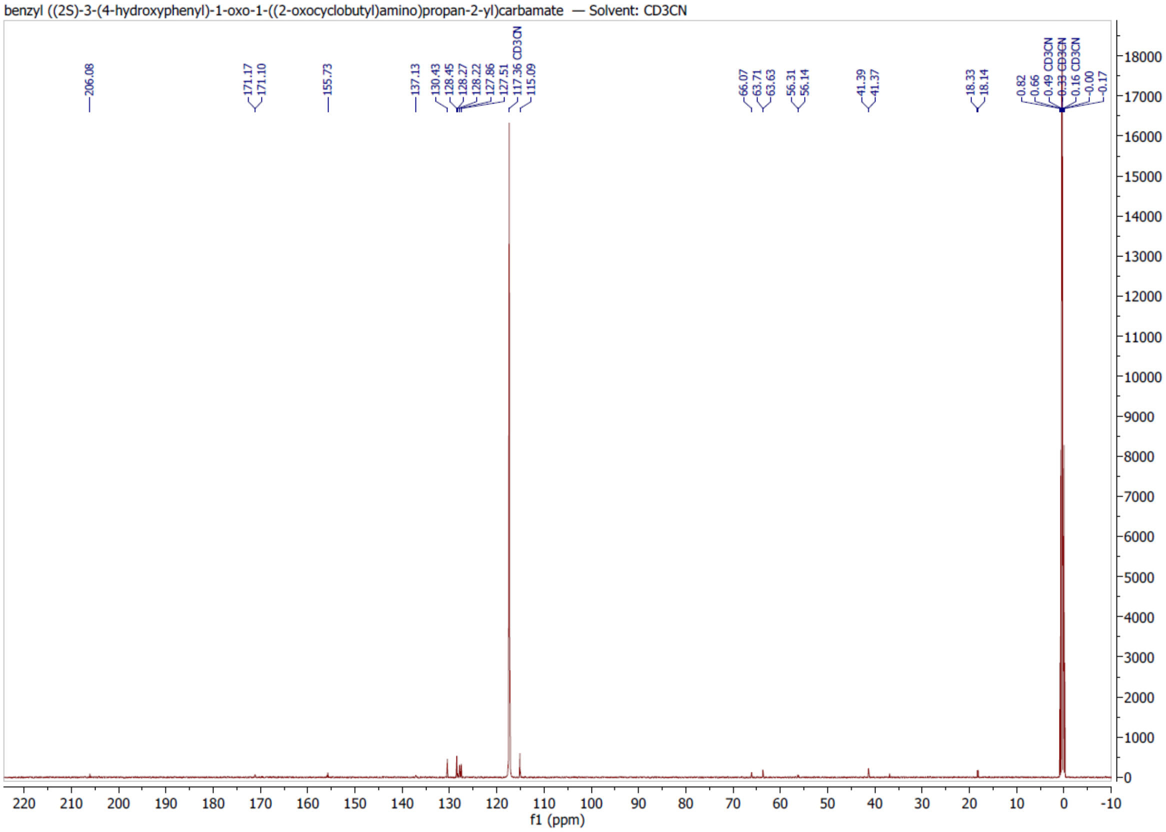

Figure S63. ^{13}C NMR (126 MHz, $CDCl_3$) of benzyl ((*2S*)-3-(4-hydroxyphenyl)-1-oxo-1-((2-oxocyclobutyl)amino)propan-2-yl)carbamate (**3af**).

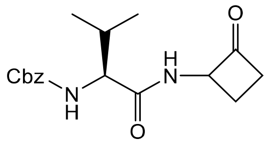

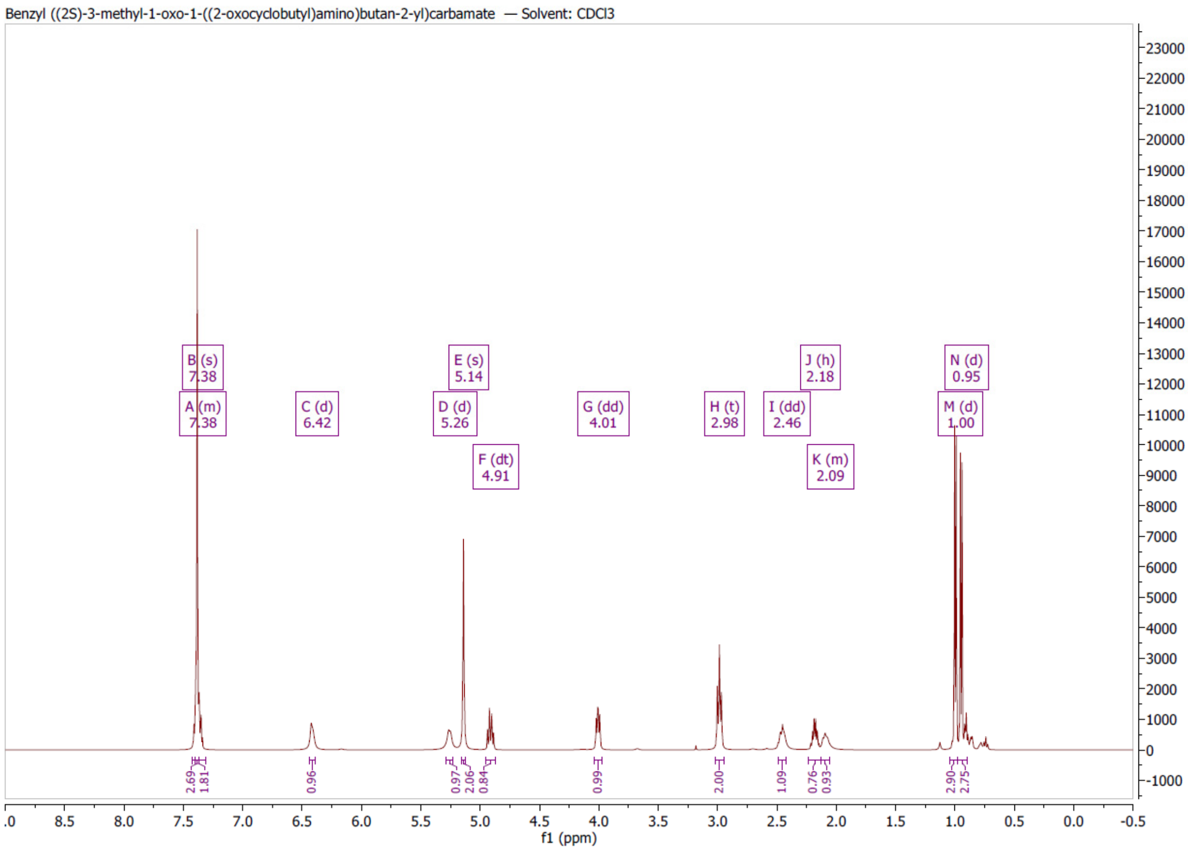

Figure S64. ^{1}H NMR (500 MHz $CDCl_3$) of benzyl ((2*S*)-3-methyl-1-oxo-1-((2-oxocyclobutyl)amino)butan-2-yl)carbamate (**3ag**).

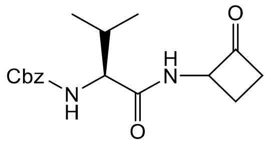

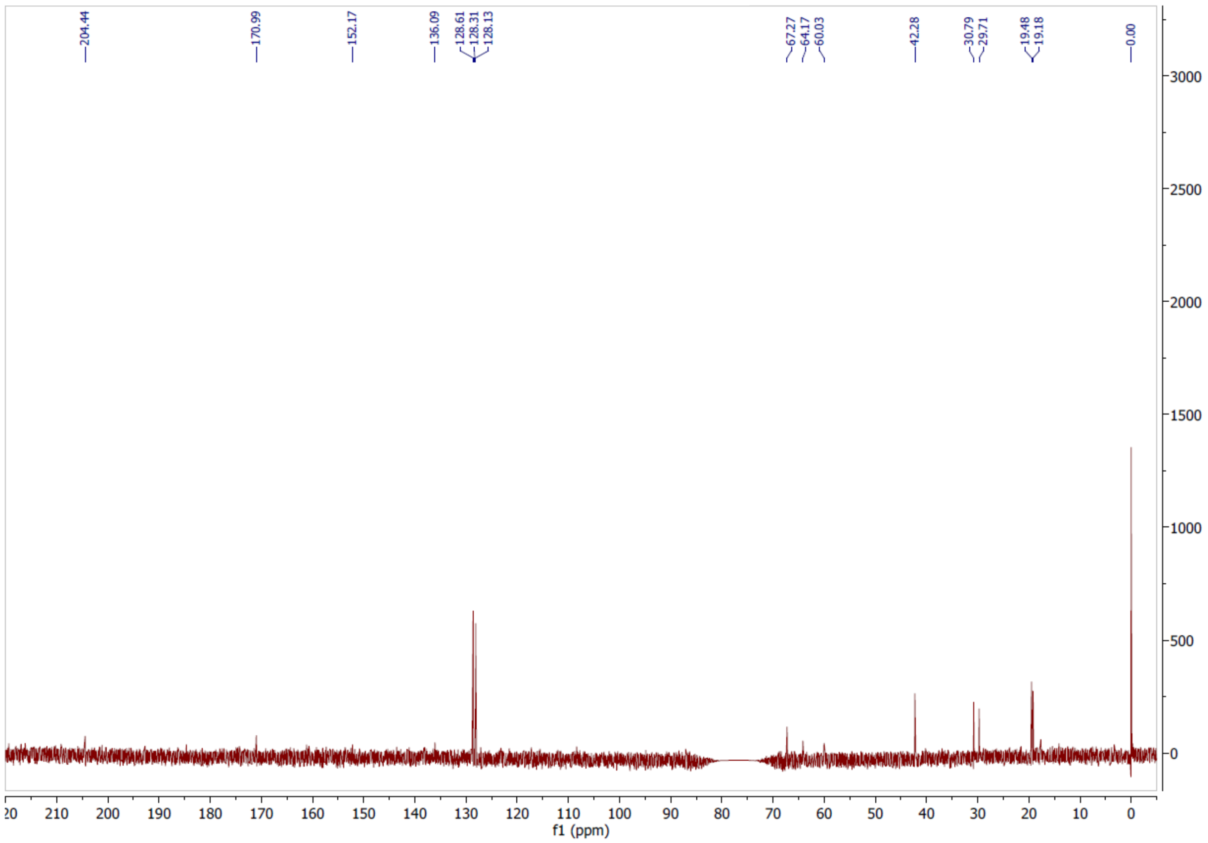

Figure S65. ^{13}C NMR (126 MHz, $CDCl_3$) of benzyl ((*2S*)-3-methyl-1-oxo-1-((2-oxocyclobutyl)amino)butan-2-yl)carbamate (**3ag**).

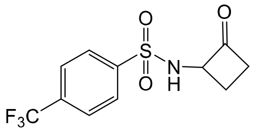

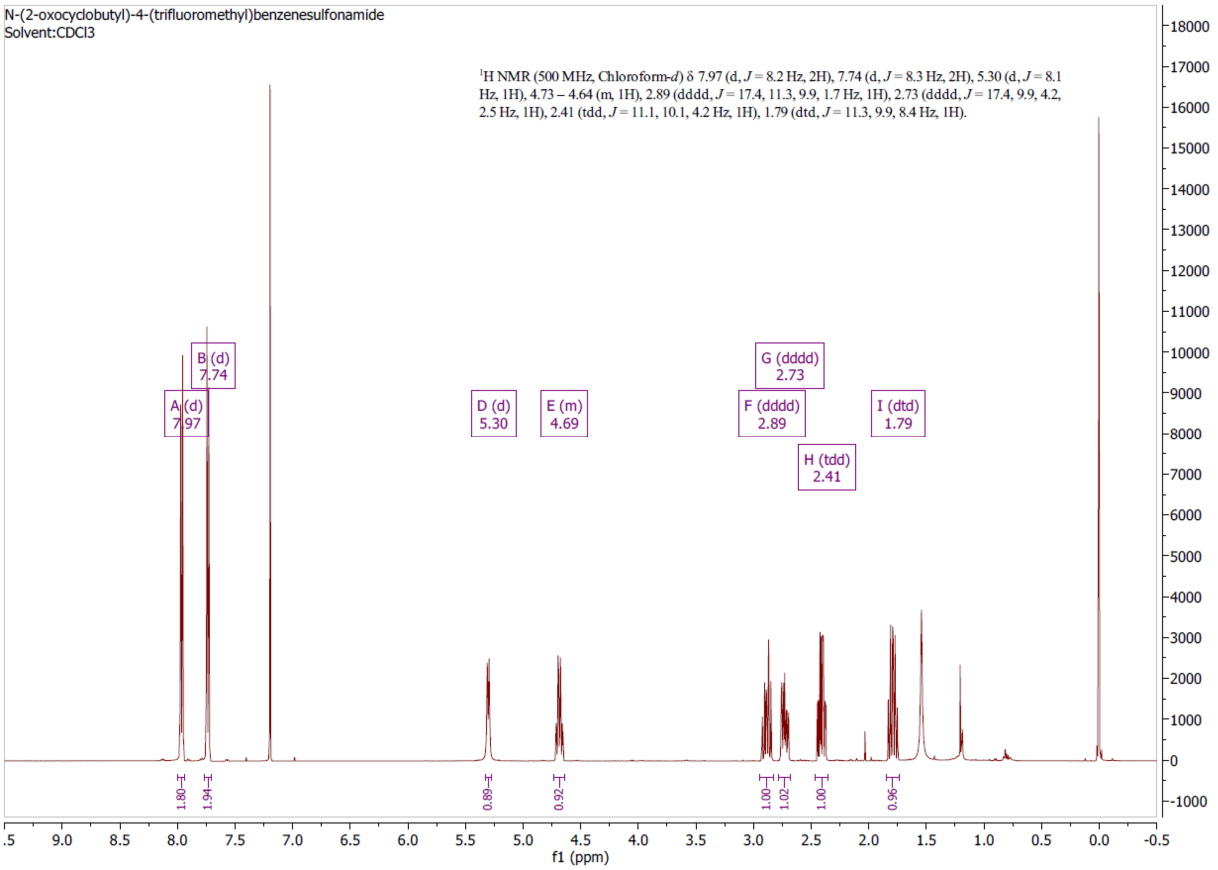

Figure S66. ^{1}H NMR (500 MHz $CDCl_3$) of *N*-(2-oxocyclobutyl)-4-(trifluoromethyl)benzenesulfonamide (**3ah**).

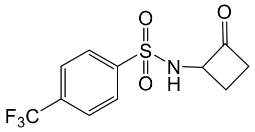

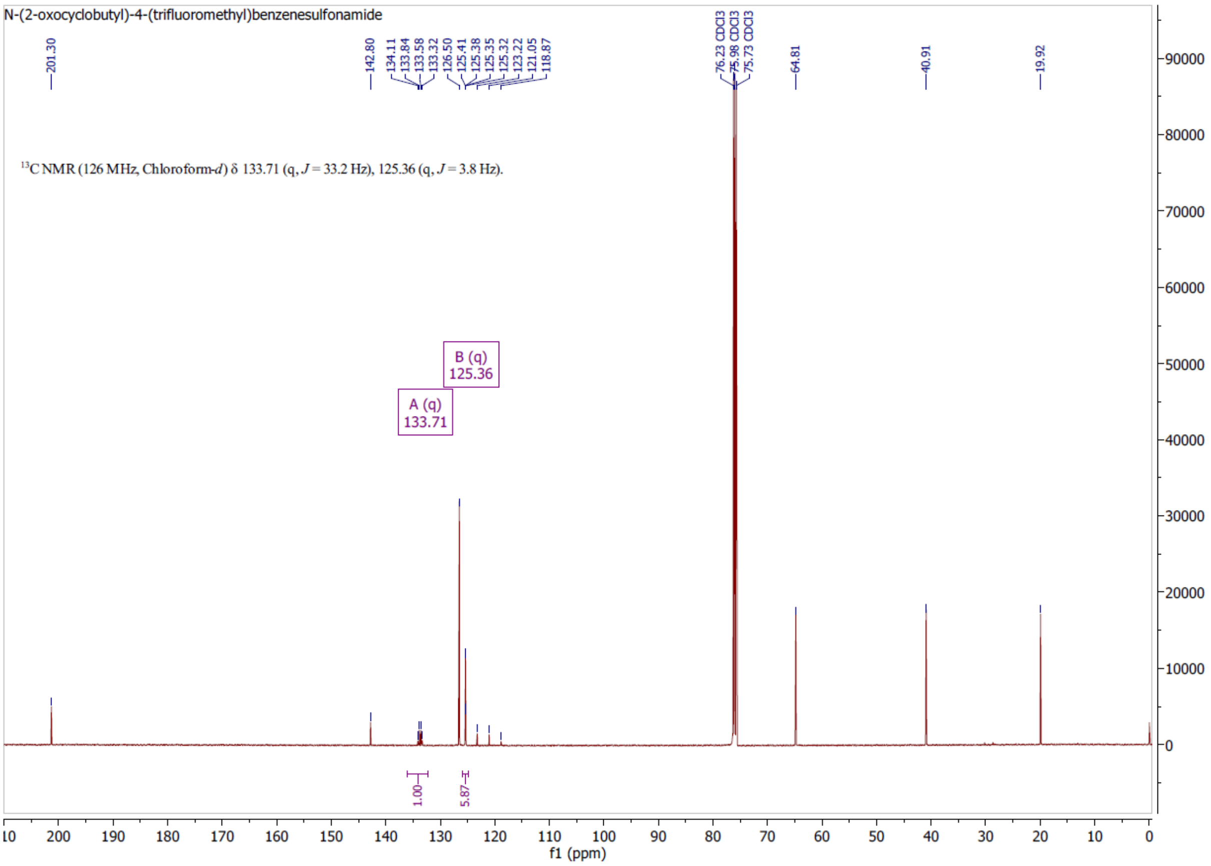

Figure S67. ^{13}C NMR (126 MHz, $CDCl_3$) of *N*-(2-oxocyclobutyl)-4-(trifluoromethyl)benzenesulfonamide (**3ah**).

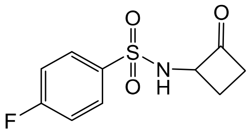

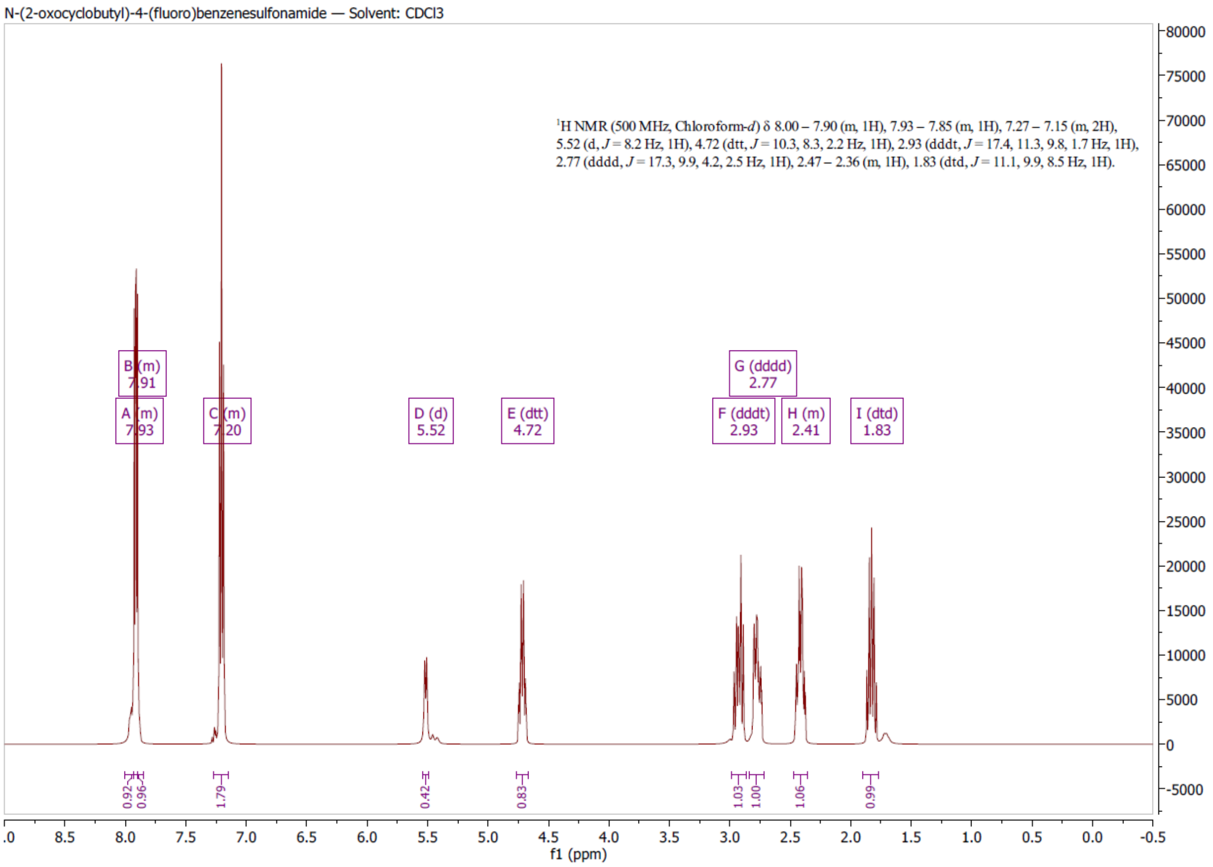

Figure S68. ^{1}H NMR (500 MHz $CDCl_3$) of *N*-(2-oxocyclobutyl)-4-(fluoro)benzenesulfonamide (**3aj**).

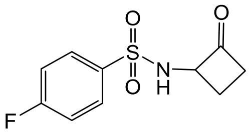

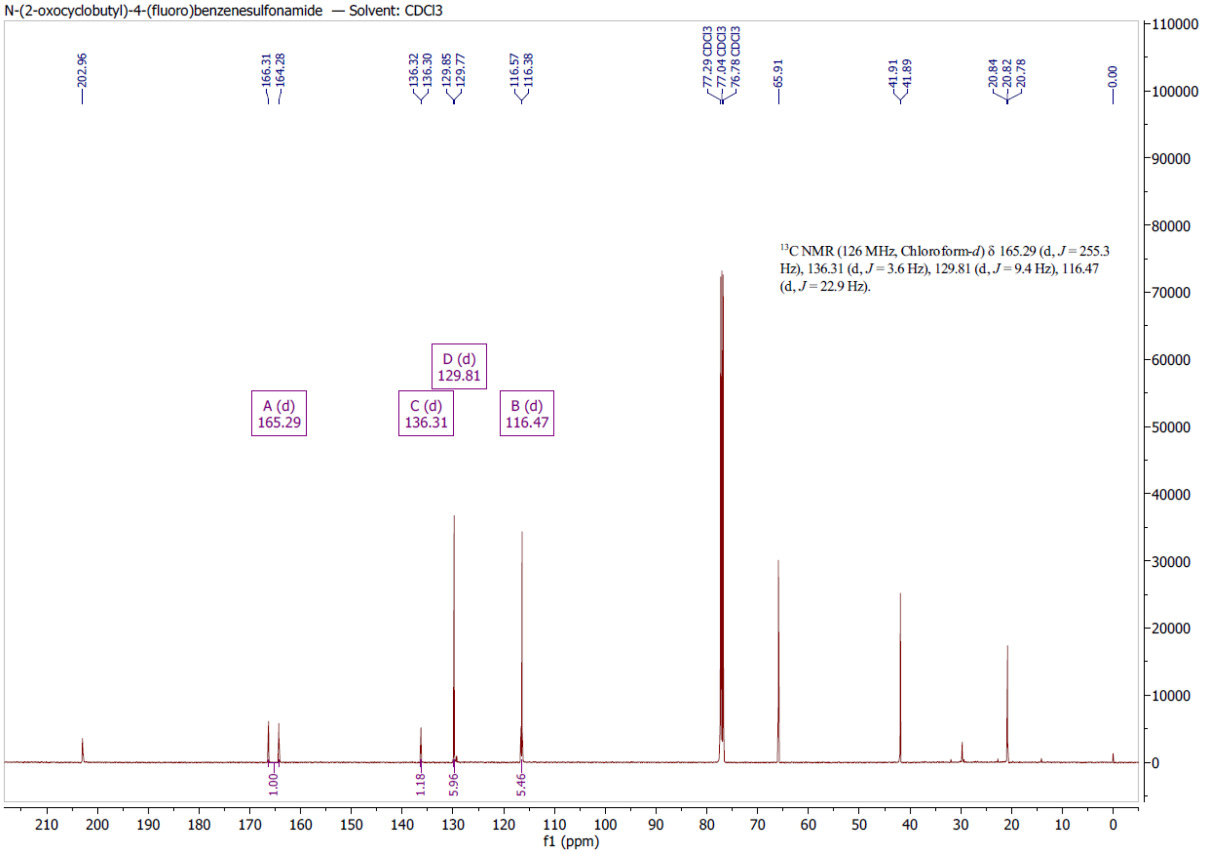

Figure S69. ^{13}C NMR (126 MHz, $CDCl_3$) of *N*-(2-oxocyclobutyl)-4-(fluoro)benzenesulfonamide (**3aj**).

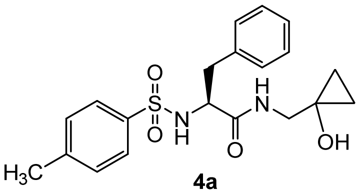

4a

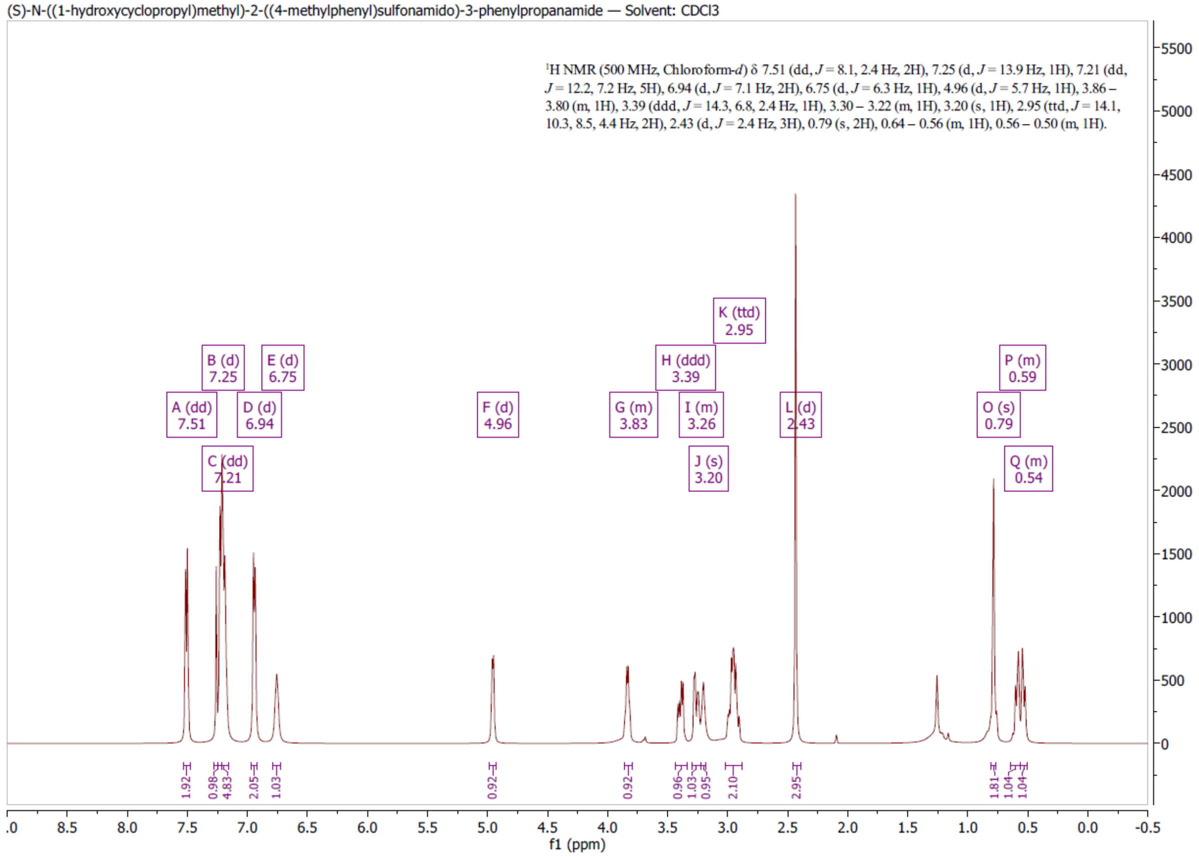

Figure S70. ^{1}H NMR (500 MHz $CDCl_3$) of (S)-*N*-((1-hydroxycyclopropyl)methyl)-2-((4-methylphenyl)sulfonamido)-3-phenylpropanamide (**4a**).

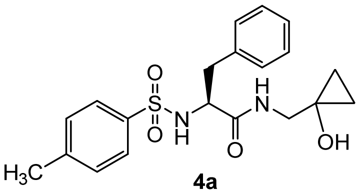

4a

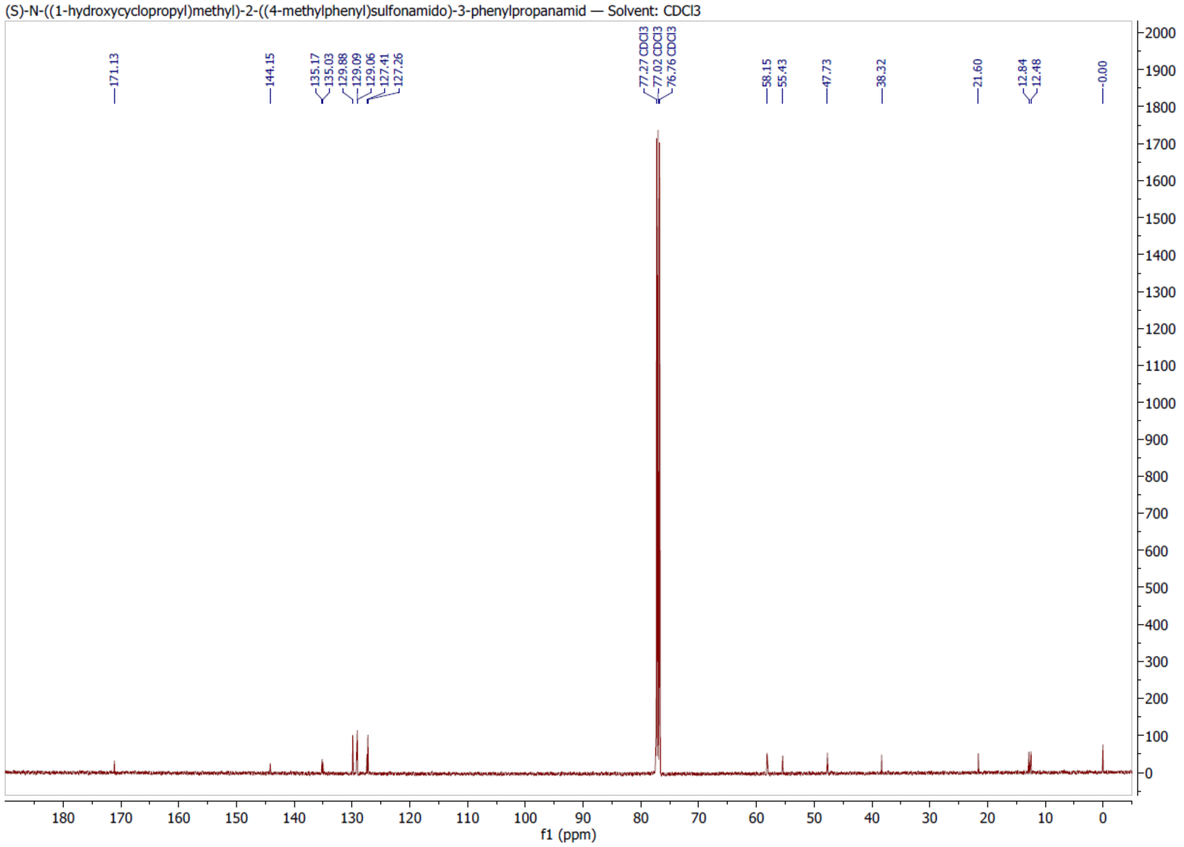

Figure S71. ^{13}C NMR (126 MHz, $CDCl_3$) of (S)-*N*-((1-hydroxycyclopropyl)methyl)-2-((4-methylphenyl)sulfonamido)-3-phenylpropanamide (**4a**).

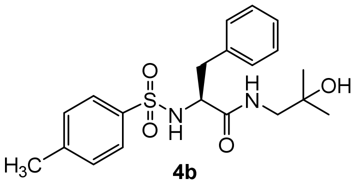

4b

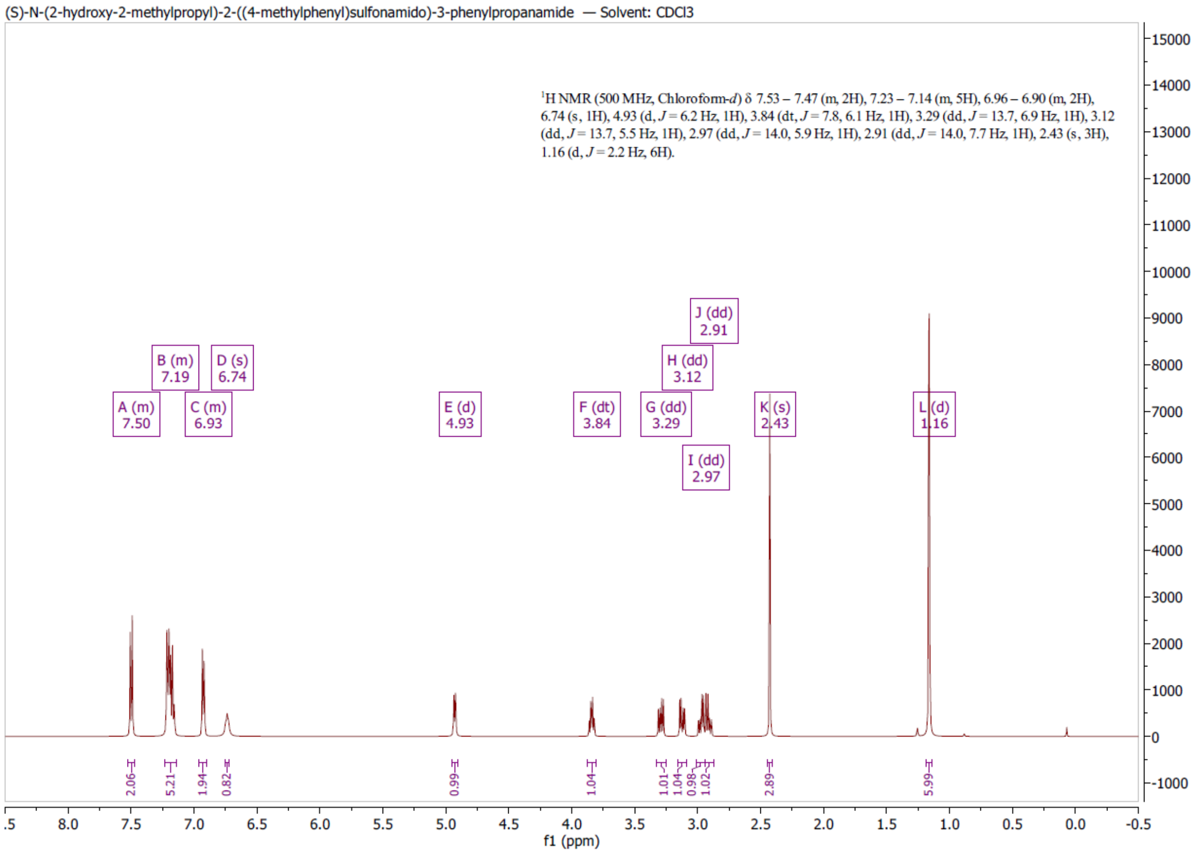

Figure S72. ^{1}H NMR (500 MHz $CDCl_3$) of (S)-*N*-(2-hydroxy-2-methylpropyl)-2-((4-methylphenyl)sulfonamido)-3-phenylpropanamide (**4b**).

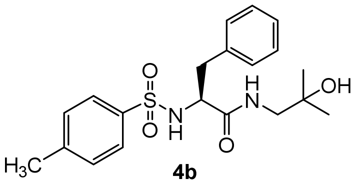

4b

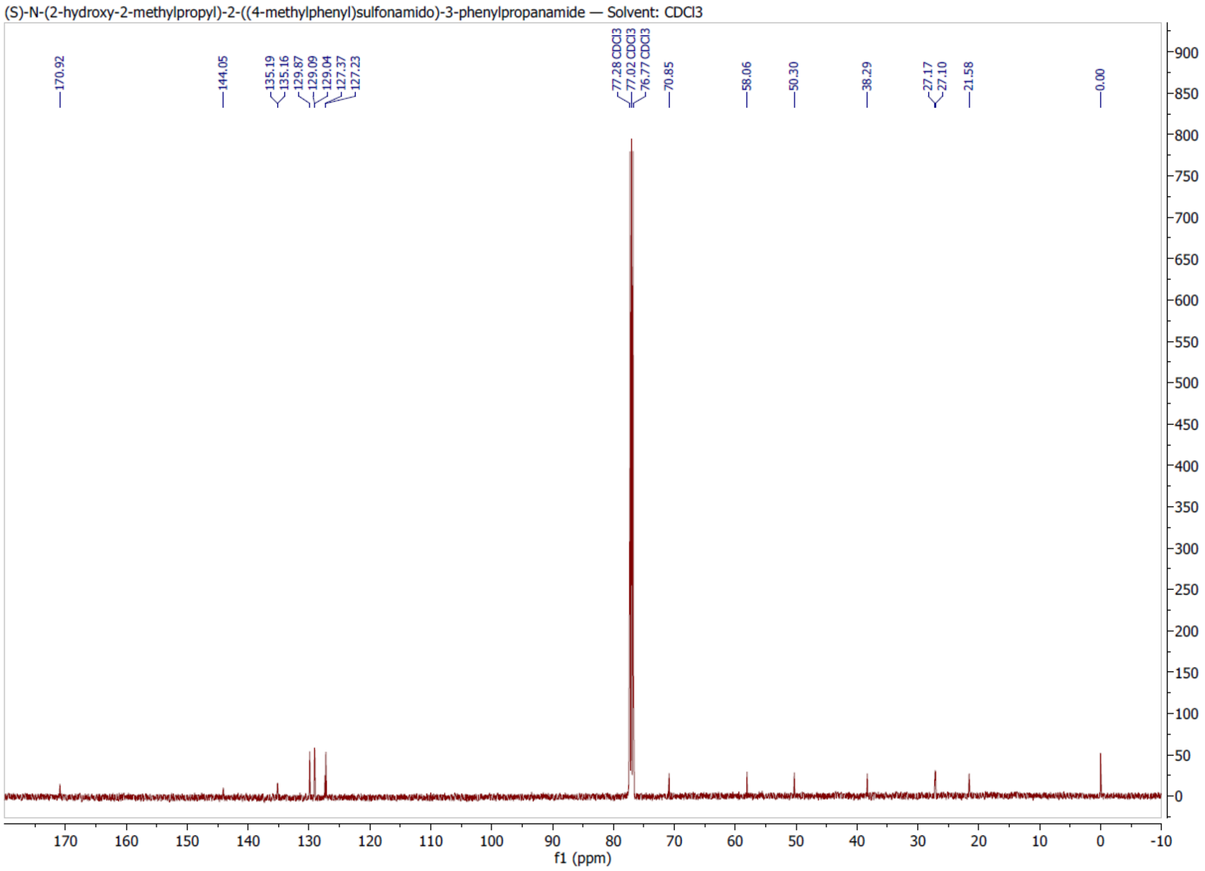

Figure S73. ^{13}C NMR (126 MHz, $CDCl_3$) of (S)-*N*-(2-hydroxy-2-methylpropyl)-2-((4-methylphenyl)sulfonamido)-3-phenylpropanamide (**4b**).

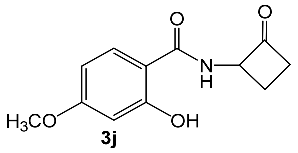

3j

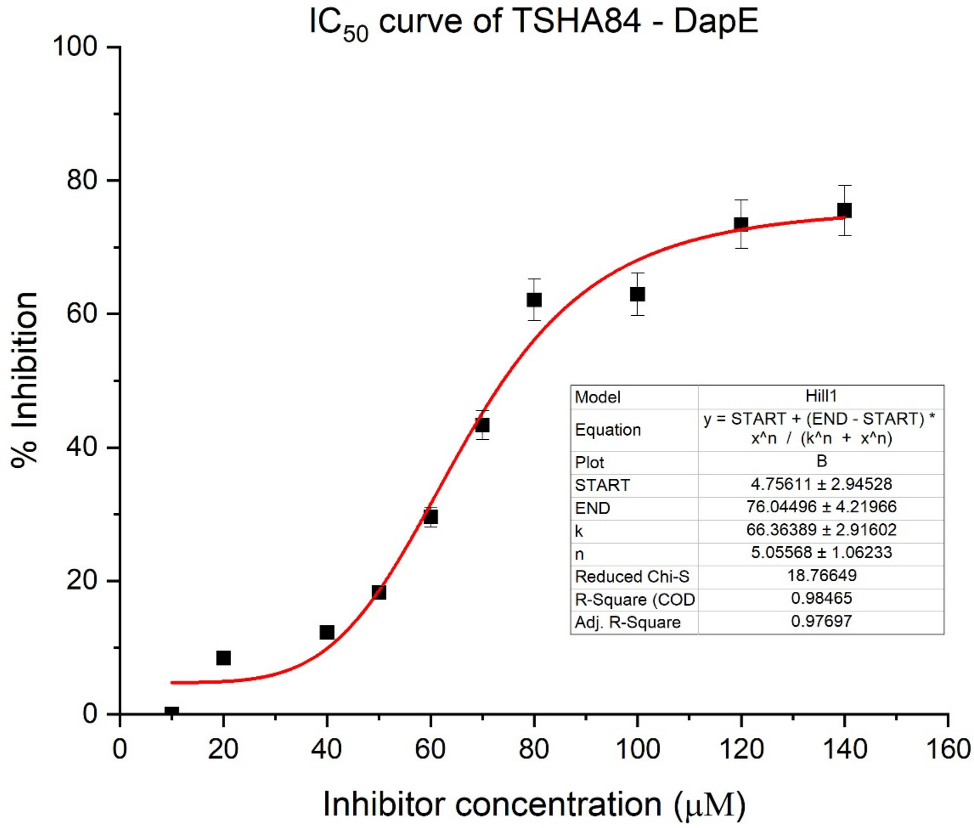

Figure S74. IC_{50} plot of 2-hydroxy-4-methoxy-*N*-(2-oxocyclobutyl)benzamide (**3j**).

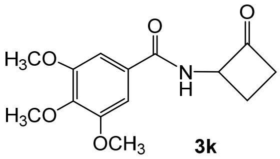

3k

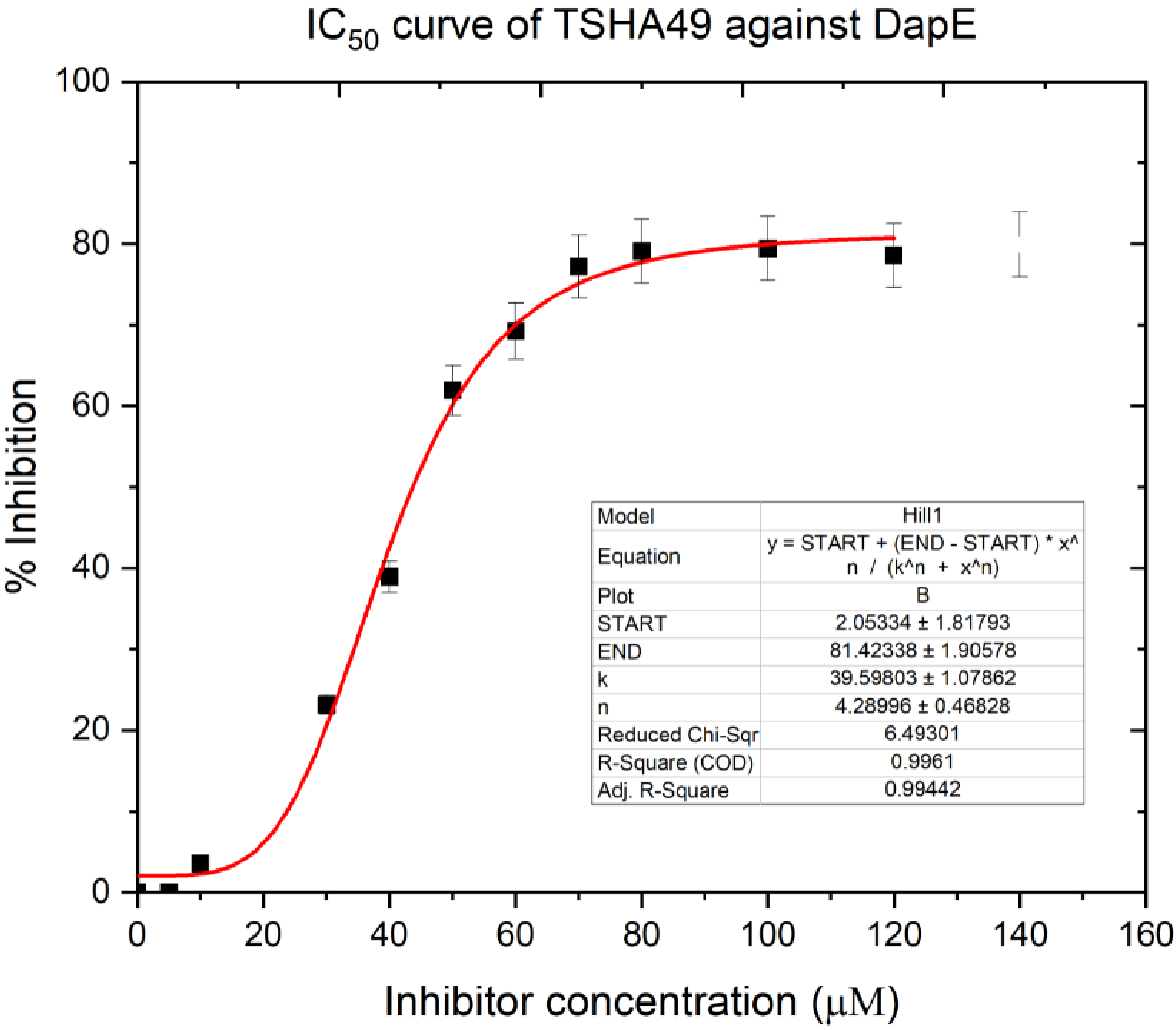

Figure S75. IC_{50} plot of 3,4,5-trimethoxy-*N*-(2-oxocyclobutyl)benzamide (**3k**).

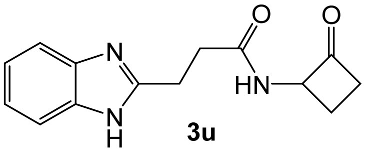

3u

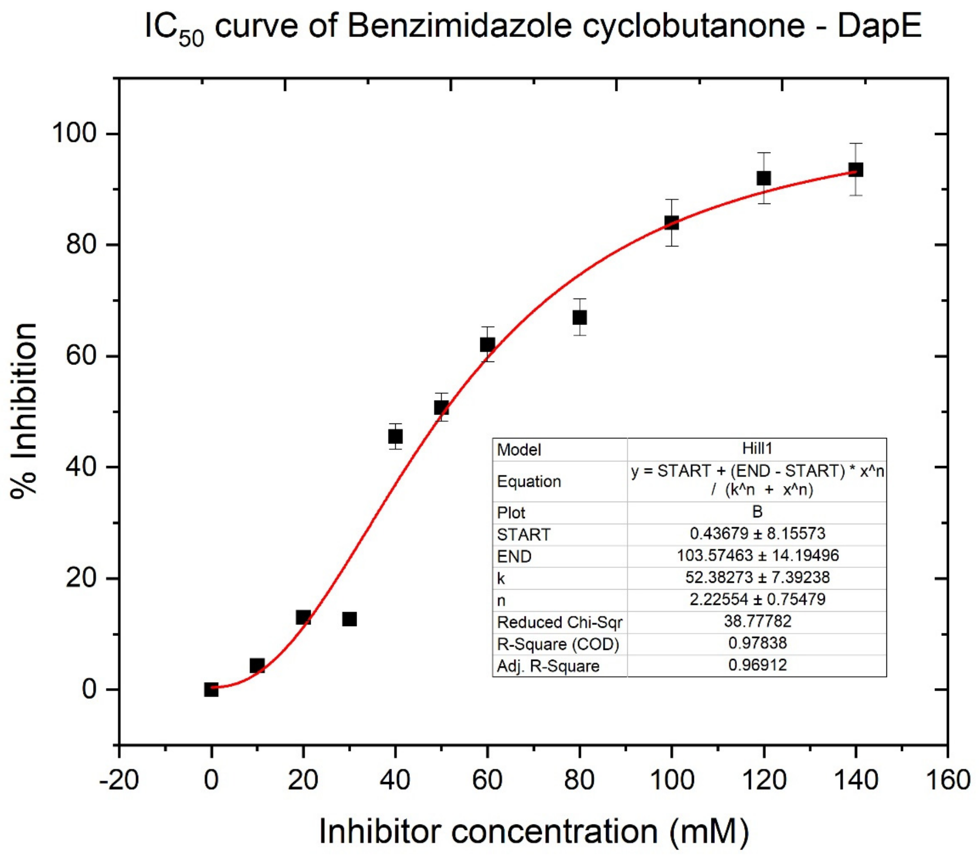

Figure S76. IC_{50} plot of 3-(*1H*-benzo[*d*]imidazol-2-yl)-*N*-(2-oxocyclobutyl)propenamide (**3u**).

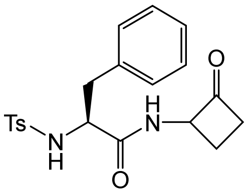

3v

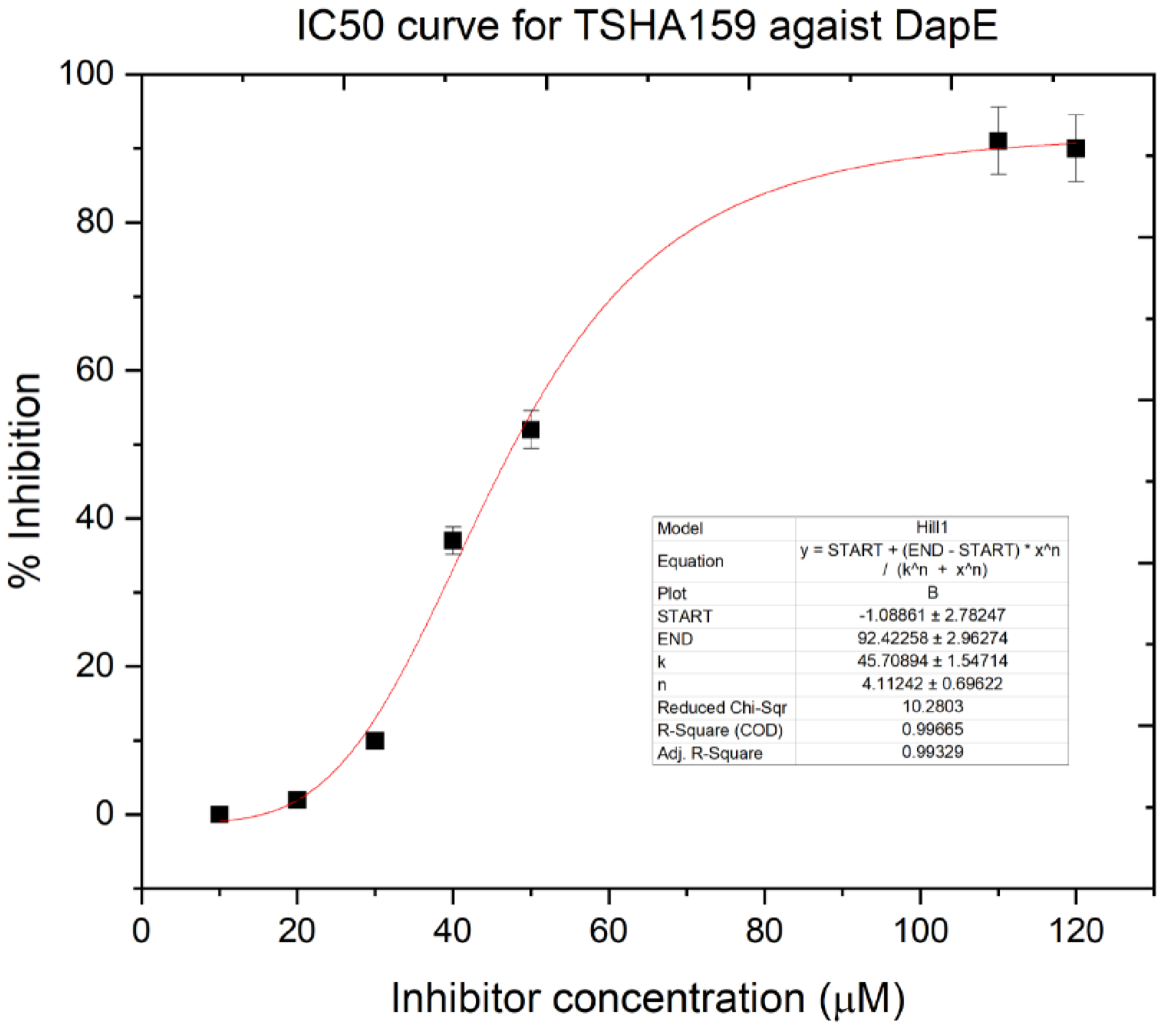

Figure S77. IC_{50} plot of (*2S*)-2-((4-methylphenyl)sulfonamido)-*N*-(2-oxocyclobutyl)-3-phenylpropanamide (**3v**).

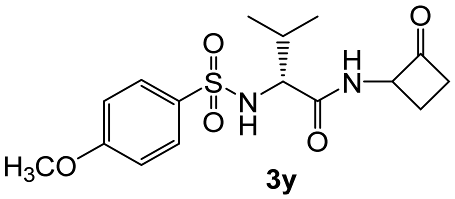

3y

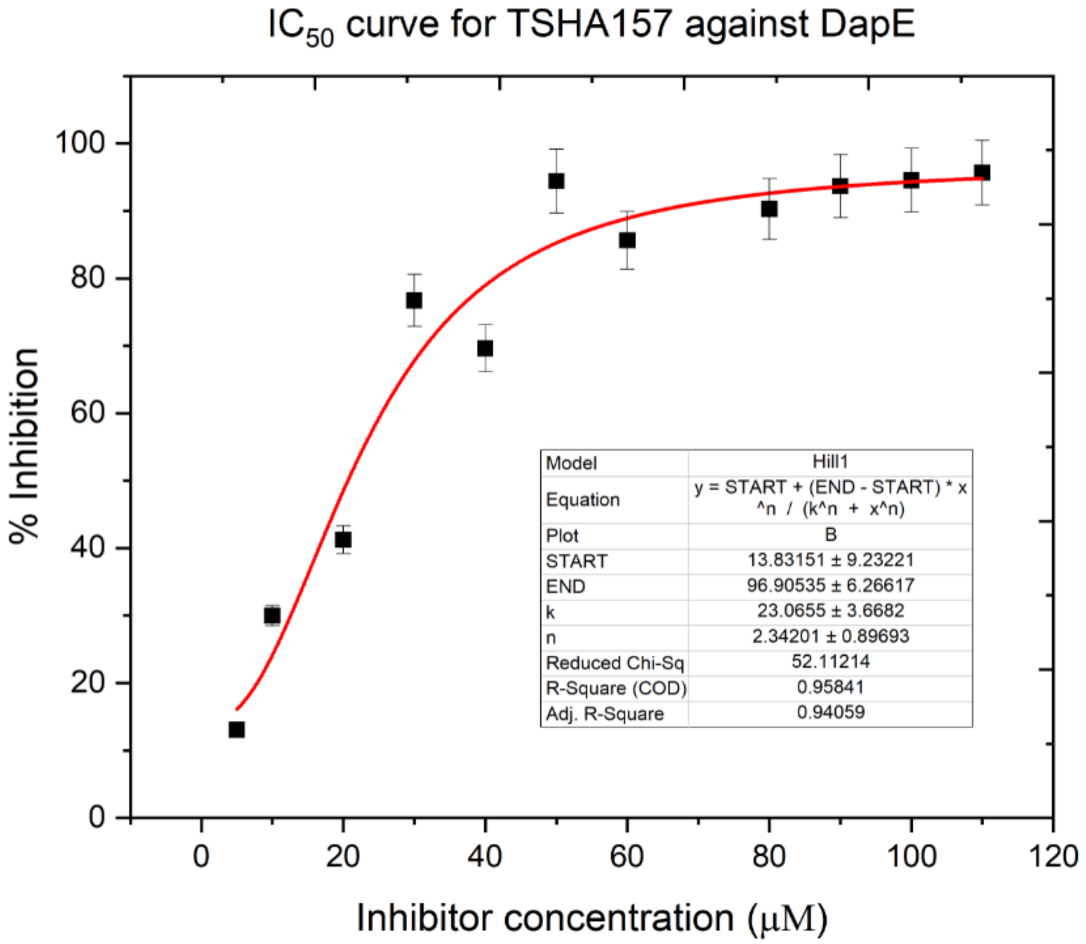

Figure S78. IC_{50} plot of (*2R*)-2-((4-methoxyphenyl)sulfonamido)-3-methyl-*N*-(2-oxocyclobutyl)butanamide (**3y**).

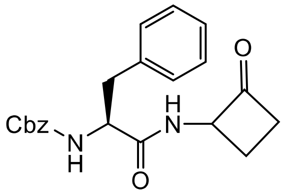

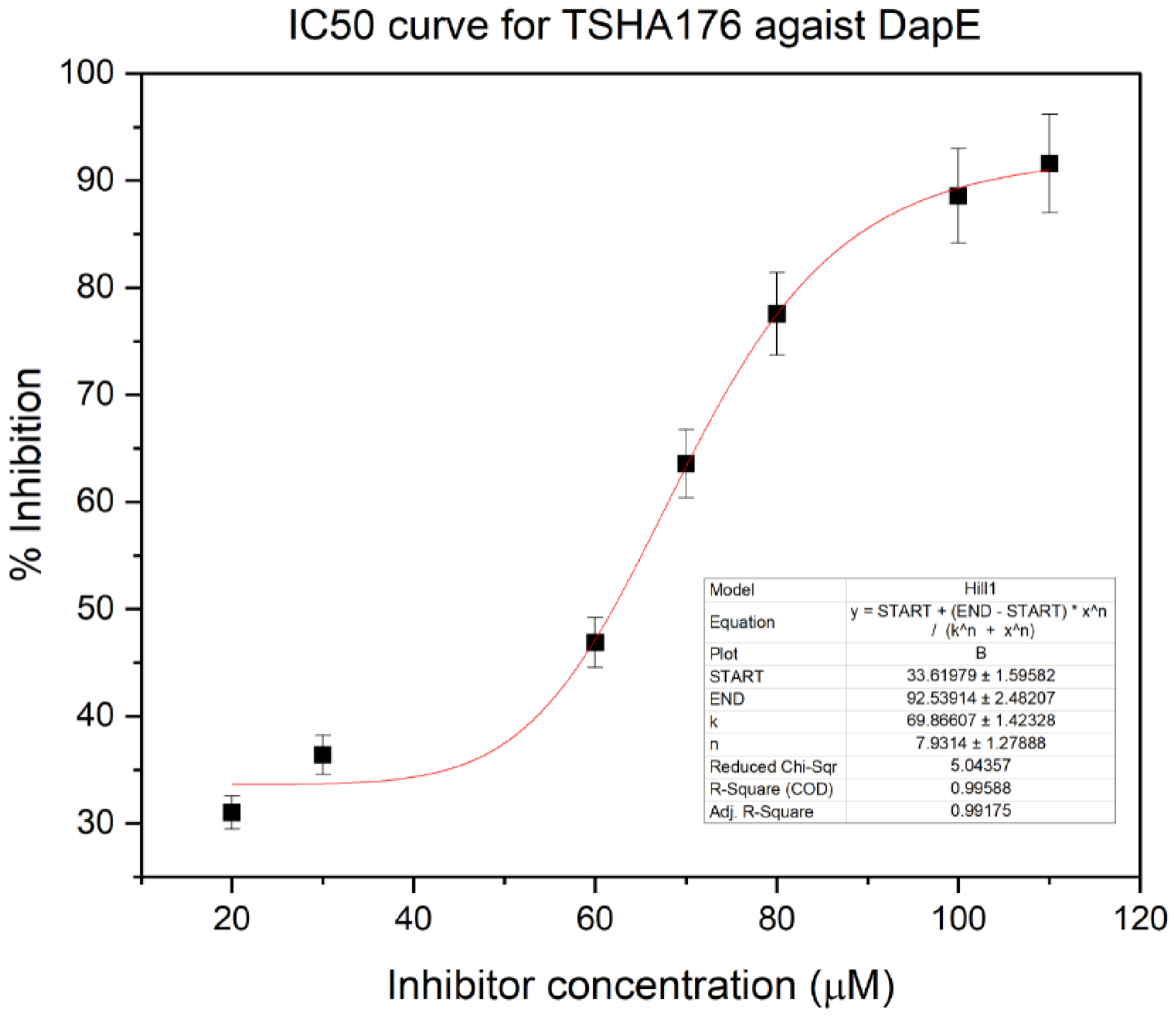

Figure S79. IC_{50} plot of benzyl ((2*R*)-1-oxo-1-((2-oxocyclobutyl)amino)-3-phenylpropan-2-yl)carbamate (**3ad**).

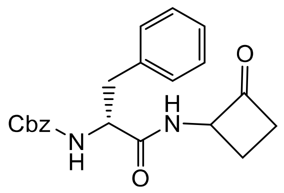

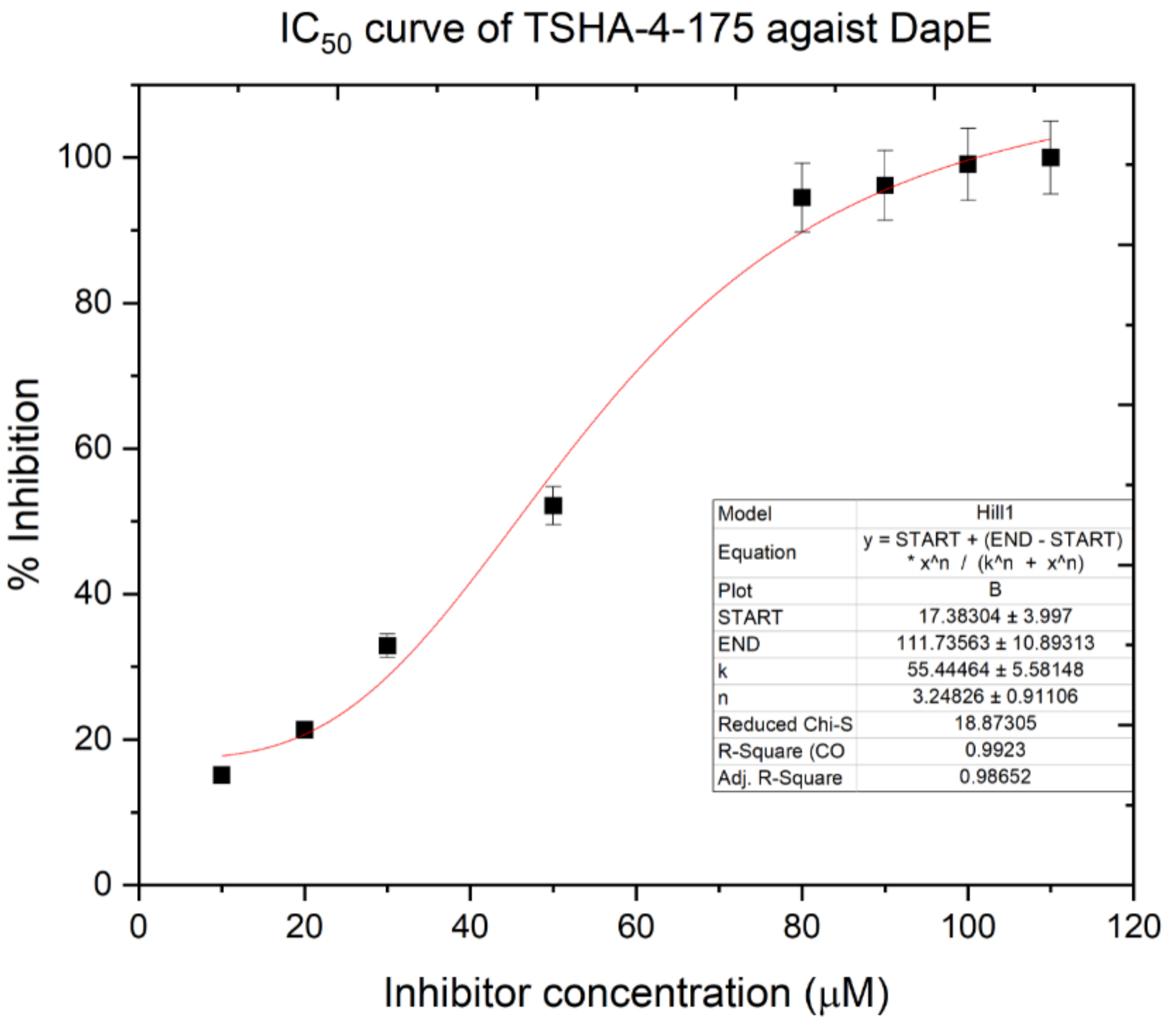

Figure S80. IC_{50} plot of benzyl ((*2R*)-1-oxo-1-((2-oxocyclobutyl)amino)-3-phenylpropan-2-yl)carbamate (**3ae**).

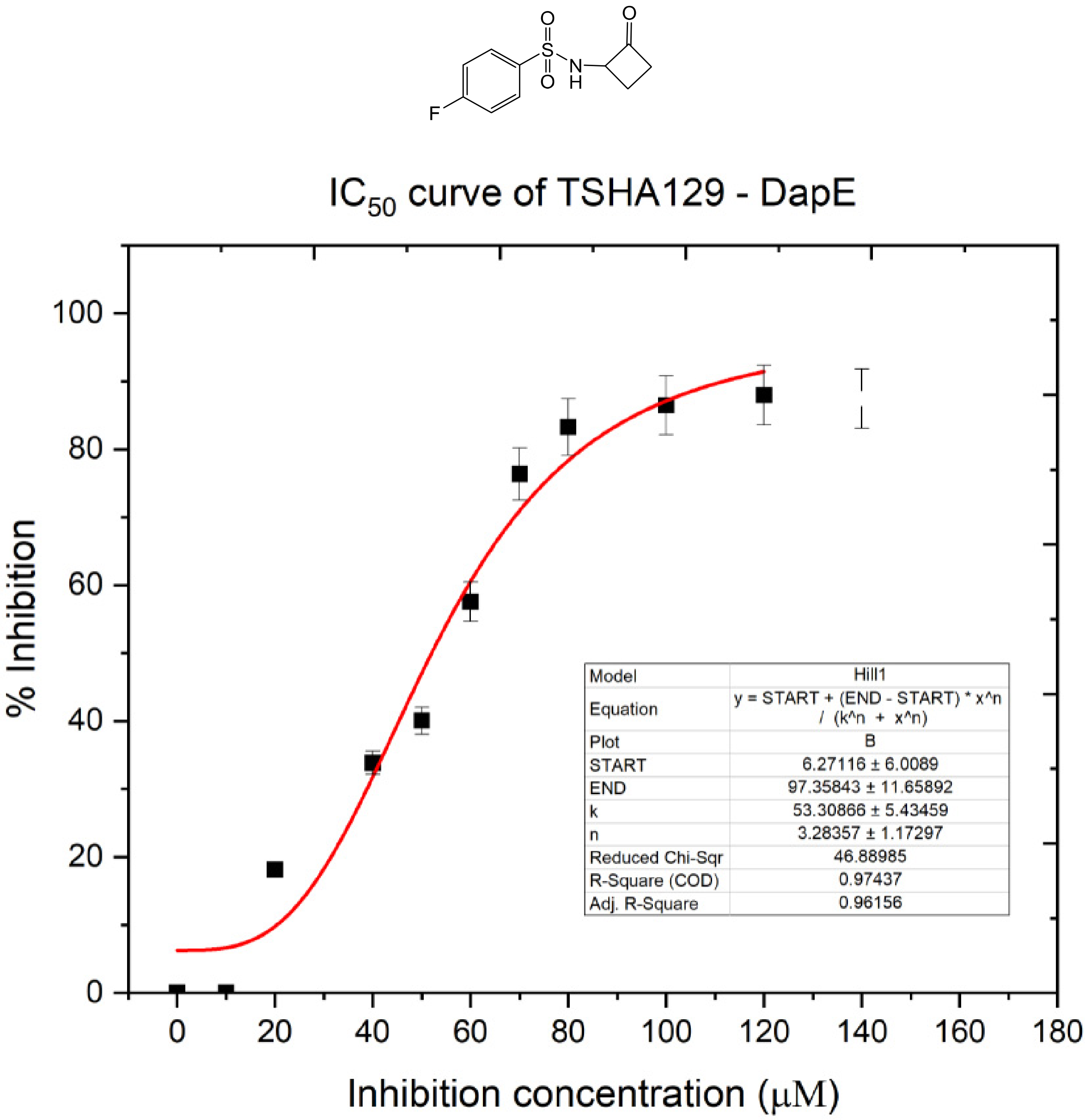

Figure S81. IC_{50} plot of *N*-(2-oxocyclobutyl)-4-(fluoro)benzenesulfonamide (**3ah**).

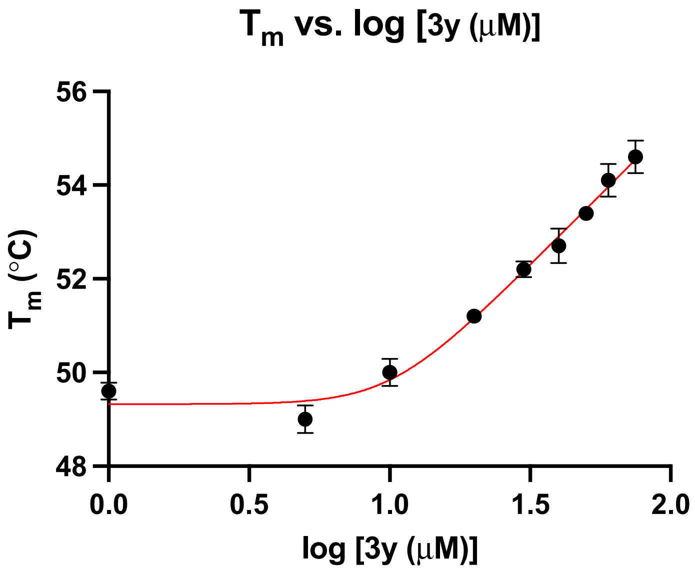

Figure S82. Thermal Shift Assay Graph of T_m vs. log [**3y** (μM)].

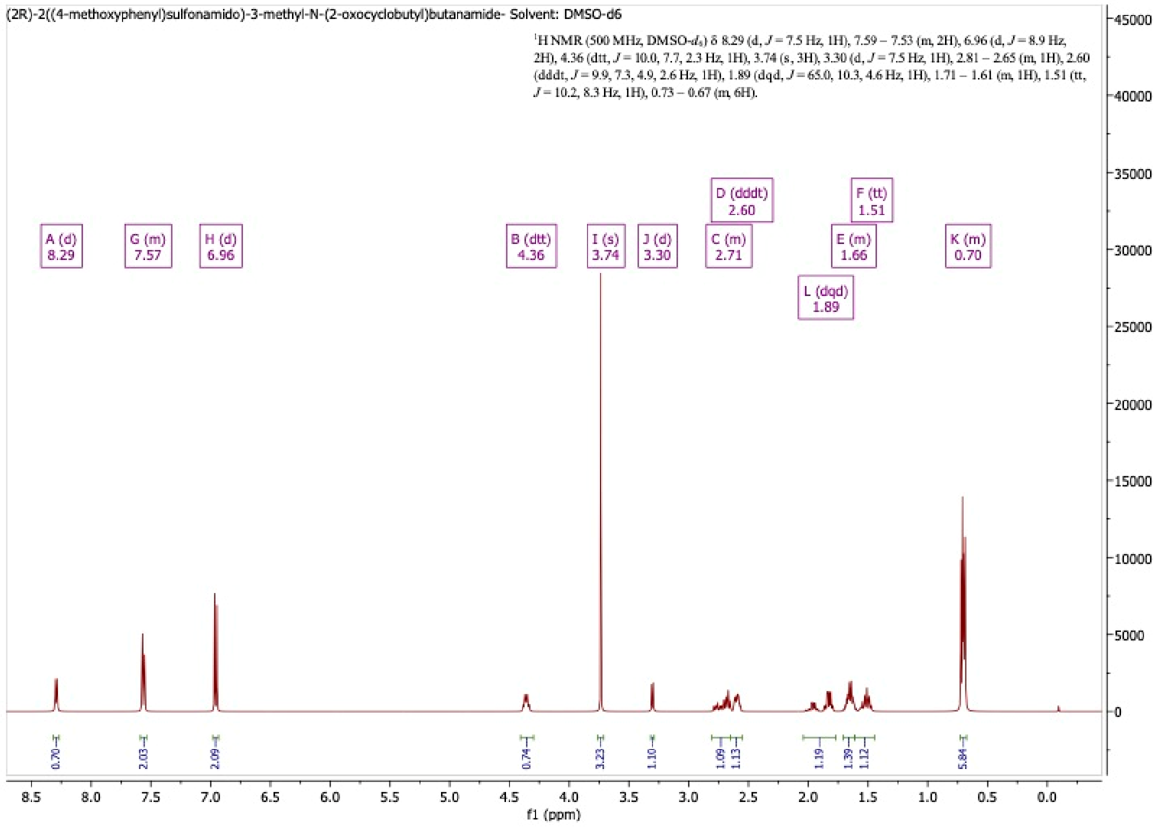

Figure S83. ^{1}H NMR (500 MHz DMSO-d6) of (*2R*)-2-((4-methoxyphenyl)sulfonamido)-3-methyl-*N*-(2-oxocyclobutyl)butanamide (**3y**).

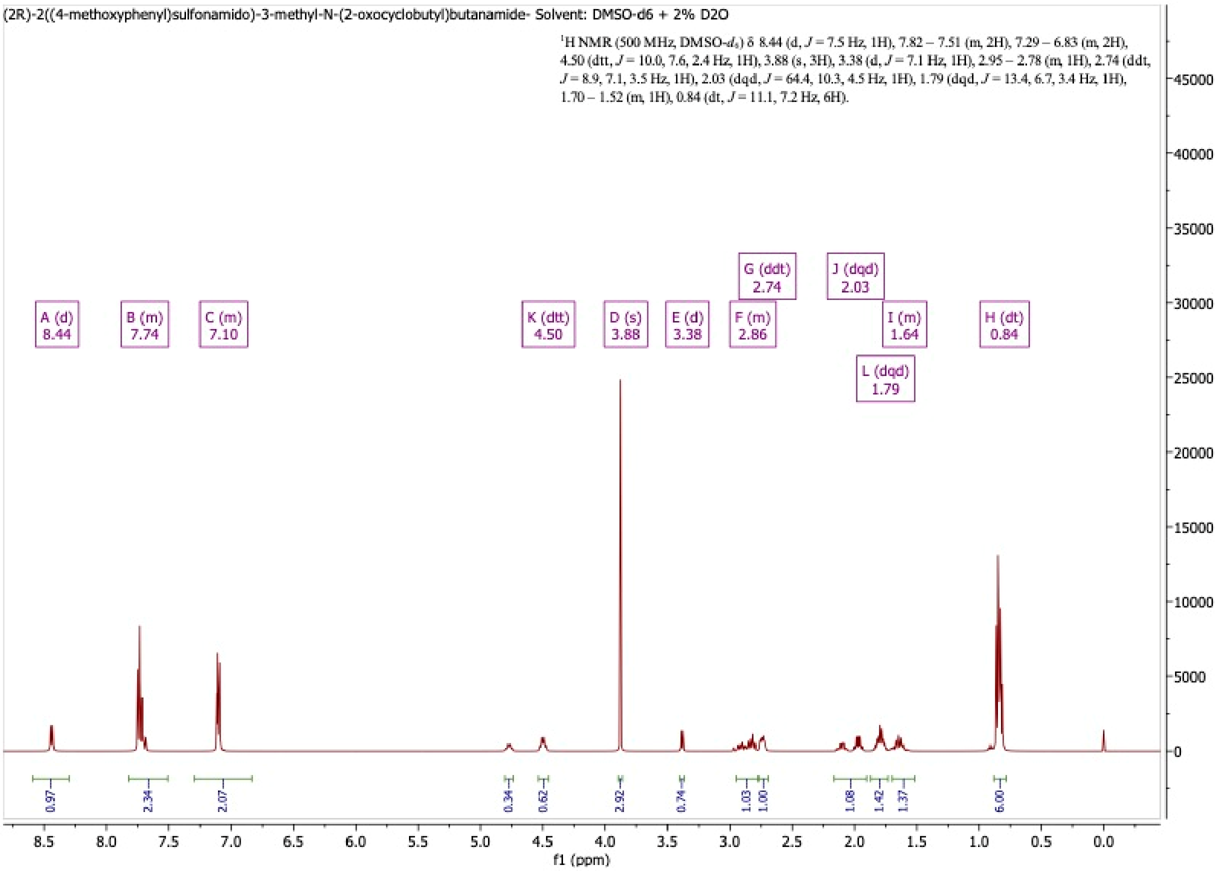

Figure S84. ^{1}H NMR (500 MHz DMSO-d6 + 2% D_2O) of (*2R*)-2-((4-methoxyphenyl)sulfonamido)-3-methyl-*N*-(2-oxocyclobutyl)butanamide (**3y**).

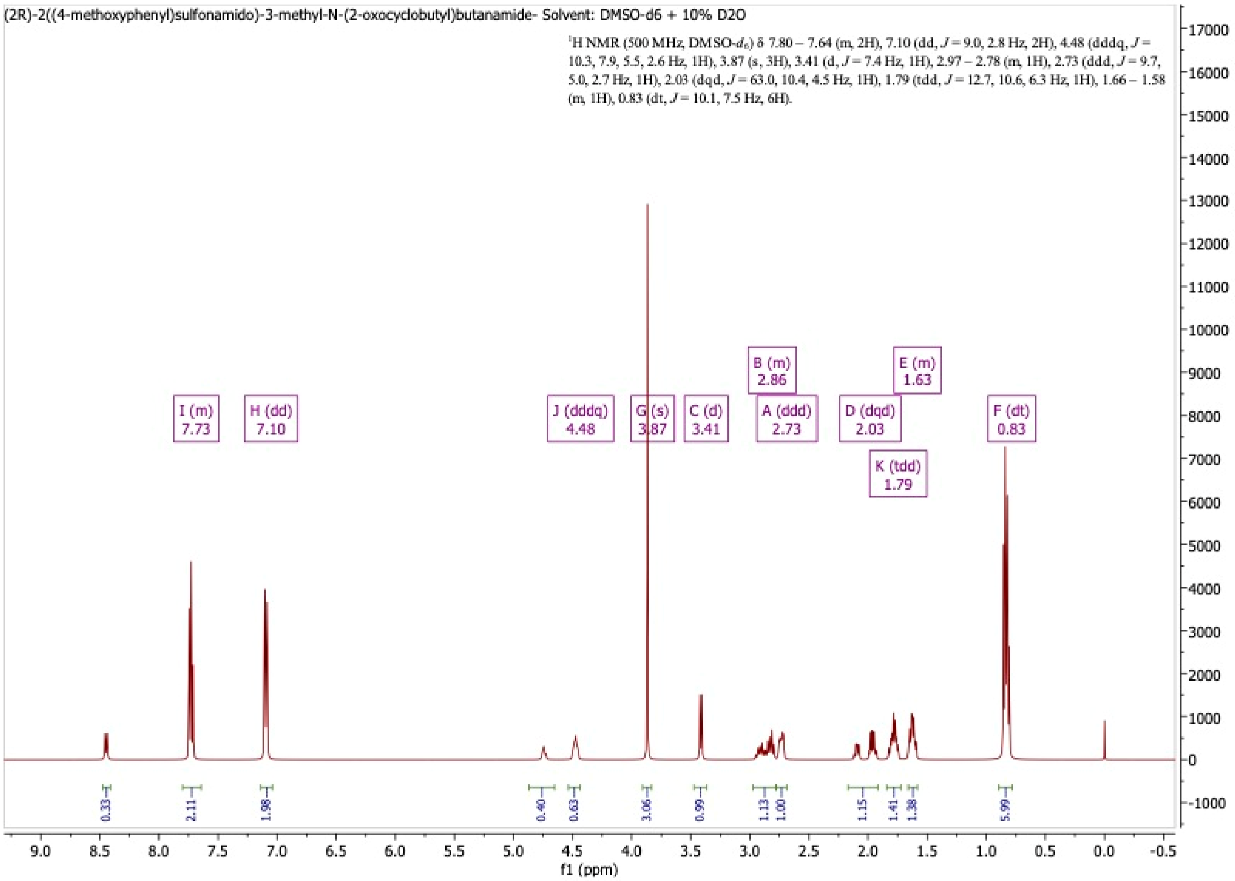

Figure S85. ^{1}H NMR (500 MHz DMSO-d6 + 10% D_2O) of (2*R*)-2-((4-methoxyphenyl)sulfonamido)-3-methyl-*N*-(2-oxocyclobutyl)butanamide (**3y**).

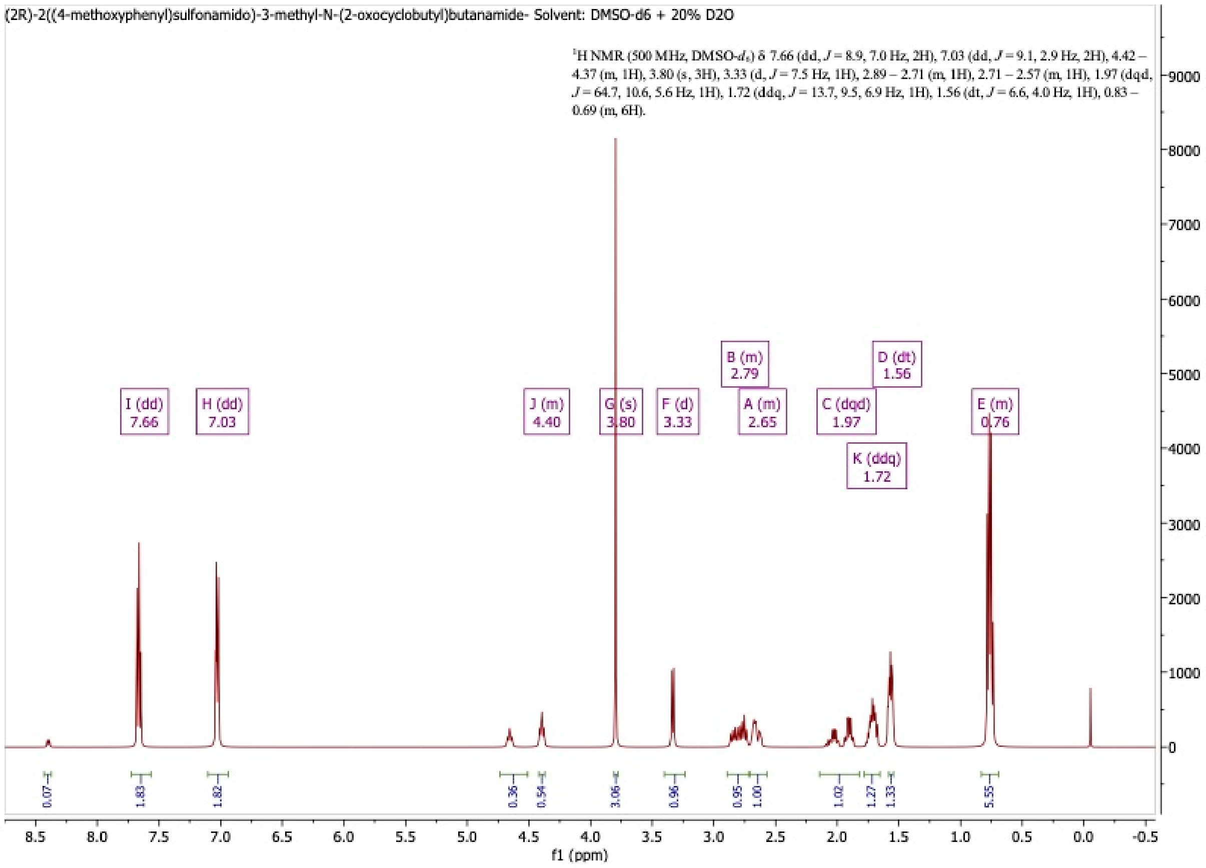

Figure S86. ^{1}H NMR (500 MHz DMSO-d6 + 20% D_2O) of (2*R*)-2-((4-methoxyphenyl)sulfonamido)-3-methyl-*N*-(2-oxocyclobutyl)butanamide (**3y**).

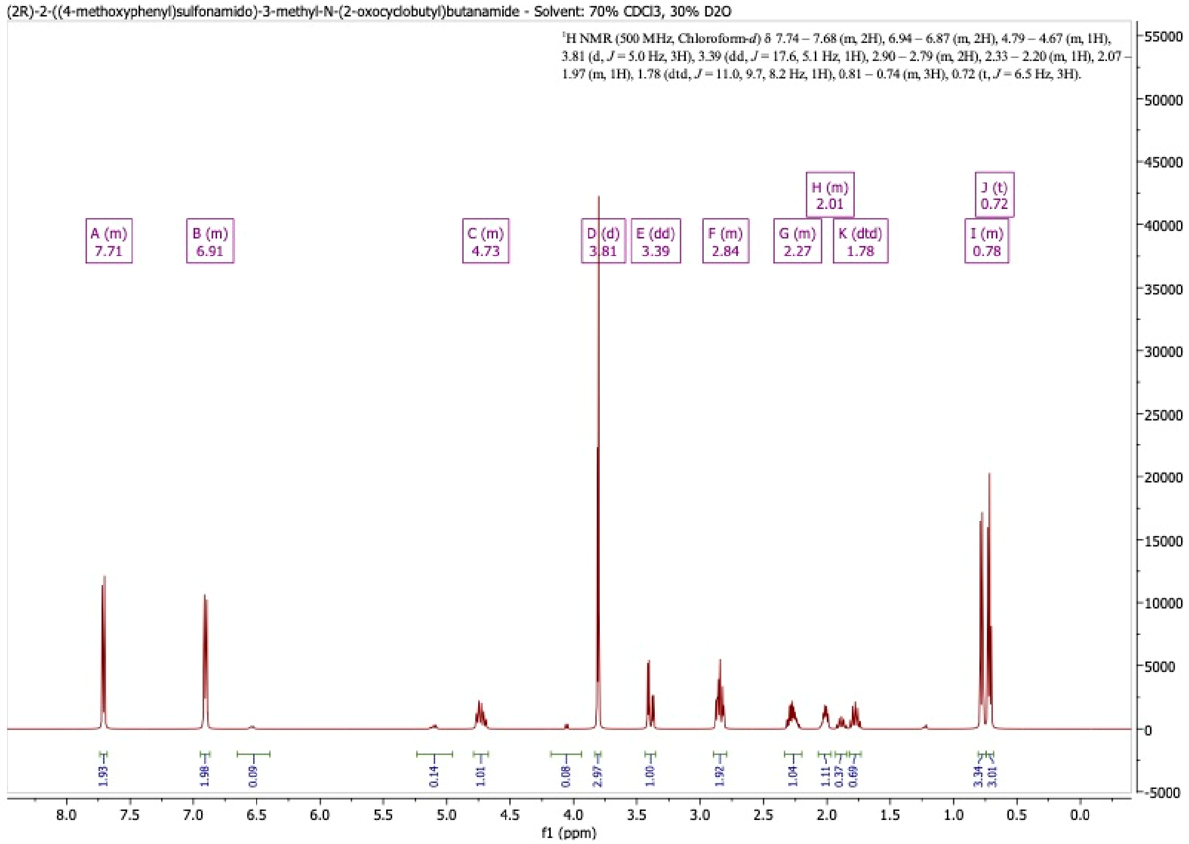

Figure S87. ^{1}H NMR (500 MHz $CDCl_3$ + 30% D_2O) of (*2R*)-2-((4-methoxyphenyl)sulfonamido)-3-methyl-*N*-(2-oxocyclobutyl)butanamide (**3y**).
